# Poultry population dynamics and mortality risks in smallholder farms of the Mekong river delta region

**DOI:** 10.1186/s12917-019-1949-y

**Published:** 2019-06-17

**Authors:** Alexis Delabouglise, Benjamin Nguyen-Van-Yen, Nguyen Thi Le Thanh, Huynh Thi Ai Xuyen, Phung Ngoc Tuyet, Ha Minh Lam, Maciej F. Boni

**Affiliations:** 1 Center for Infectious Disease Dynamics, Department of Biology, Pennsylvania State University, Millenium Sciences Complex, Pollock road, University Park, PA 16802 USA; 2 Oxford University Clinical Research Unit, Wellcome Trust Major Overseas Programme, Ho Chi Minh City, Vietnam; 3 École Normale Supérieure, CNRS UMR 8197, 46 rue d’Ulm, Paris, France; 4 Ca Mau sub-Department of Livestock Production and Animal Health, Ca Mau, Vietnam; 5 Center for Tropical Medicine and Global Health, Nuffield Department of Medicine, University of Oxford, Oxford, UK

**Keywords:** Poultry production, Smallholder farms, Southeast Asia, Vietnam, Veterinary epidemiology, Livestock demography, Population biology, Infectious diseases of poultry, Avian influenza

## Abstract

**Background:**

Poultry farming is widely practiced by rural households in Vietnam and the vast majority of domestic birds are kept on small household farms. However, smallholder poultry production is constrained by several issues such as infectious diseases, including avian influenza viruses whose circulation remains a threat to public health. This observational study describes the demographic structure and dynamics of small-scale poultry farms of the Mekong river delta region.

**Method:**

Fifty three farms were monitored over a 20-month period, with farm sizes, species, age, arrival/departure of poultry, and farm management practices recorded monthly.

**Results:**

Median flock population sizes were 16 for chickens (IQR: 10–40), 32 for ducks (IQR: 18–101) and 11 for Muscovy ducks (IQR: 7–18); farm size distributions for the three species were heavily right-skewed. Muscovy ducks were kept for long periods and outdoors, while chickens and ducks were farmed indoors or in pens. Ducks had a markedly higher removal rate (broilers: 0.14/week; layer/breeders: 0.05/week) than chickens and Muscovy ducks (broilers: 0.07/week; layer/breeders: 0.01–0.02/week) and a higher degree of specialization resulting in a substantially shorter life span. The rate of mortality due to disease did not differ much among species, with birds being less likely to die from disease at older ages, but frequency of disease symptoms differed by species. Time series of disease-associated mortality were correlated with population size for Muscovy ducks (Kendall’s coefficient τ = 0.49, *p*-value < 0.01) and with frequency of outdoor grazing for ducks (τ = 0.33, p-value = 0.05).

**Conclusion:**

The study highlights some challenges to disease control in small-scale multispecies poultry farms. The rate of interspecific contact and overlap between flocks of different ages is high, making small-scale farms a suitable environment for pathogens circulation. Muscovy ducks are farmed outdoors with little investment in biosecurity and few inter-farm movements. Ducks and chickens are more at-risk of introduction of pathogens through movements of birds from one farm to another. Ducks are farmed in large flocks with high turnover and, as a result, are more vulnerable to disease spread and require a higher vaccination coverage to maintain herd immunity.

**Electronic supplementary material:**

The online version of this article (10.1186/s12917-019-1949-y) contains supplementary material, which is available to authorized users.

## Background

Poultry farming is practiced by more than 7 million households in Vietnam as a source of income and protein for consumption [1]. Most of Vietnam’s domestic poultry population (primarily chickens, ducks, and Muscovy ducks) is farmed on a small scale (< 100 bird per cycle) and the overwhelming majority of poultry farmers are smallholders [1–3]. Despite the recent development of a large-scale commercial sector, Vietnamese consumers display a strong preference for local breeds of chickens, which are adapted to small scale farming systems [4–6]. Small-scale poultry farming, by allowing households to produce meat and eggs and obtain an income with limited financial investments in infrastructure and feed, contribute to poverty alleviation, especially in remote rural areas of the country [7]. Following the definition of the Food and Agriculture Organization of the United Nations, smallholder farms are usually categorized as backyard (< 50 birds per cycle) or semi-commercial (> 50 birds per cycle) farms according to their size [8]. Small scale duck farming systems are often closely integrated with other agricultural productions like rice and fish. Their contribution to rice production is significant as duck flocks, when transported on rice fields for foraging, feed on rice crop parasites like the golden apple snail [9]. Duck flocks are usually categorized as stationary or itinerant, depending on the extent of their movements beyond the limits of their farm village [10, 11].

One major concern of smallholder farmers is the occurrence of infectious diseases, second only to market price fluctuations [12]. Among the most dreaded contagious avian diseases are Newcastle disease, infectious bursal disease (Gumboro), fowl cholera, duck viral enteritis, and avian influenza (AI), which are all endemic throughout the country despite the availability of vaccines to control them [13, 14]. Highly pathogenic avian influenza, mostly caused by H5 subtypes of AI viruses, has focused the attention of the international community due to ongoing risk of AI virus evolution and the development of a virus capable of human-to-human transmission with pandemic potential [15, 16]. The Mekong river delta, which hosts about one fifth of Vietnam’s domestic poultry [3] is one of the main high risk areas for AI because of its high density of domestic poultry and its widespread practice of itinerant duck farming and duck scavenging on flooded lands [17–19]. National-level interventions to control the disease have centered on the development of surveillance systems, preventive culling of poultry in outbreak areas, poultry movement controls, and mass poultry vaccination [20, 21]. The role played by small-scale poultry production systems in perpetuating the circulation of the disease has been debated [4, 22–24]. On the one hand, smallholder farms are believed to have very limited biosecurity practices, use little vaccination, often host multiple poultry species (most commonly chickens, ducks and Muscovy ducks), and have higher contact rates with wild birds or foraging areas frequented by wild birds, which increases their susceptibility to AI transmission. On the other hand, their small size and slow turnover (rate of birth/introduction and sale/slaughter of poultry) may limit their capacity to amplify and sustain virus circulation. In addition, these farms are less well connected to live-bird trade networks compared to larger commercial farms, which limits their capacity to spread the virus over long distances [25].

In-depth information on the population structure, demographic dynamics, biosecurity, and vaccination practices of smallholder poultry farms is therefore needed. While poultry trade networks were investigated in northern Vietnam [26] and farming practices of large itinerant duck flocks were studied in the south [11], little attention has been given to the specific management of small-scale farms. The present study aimed to collect descriptive data on the on-farm demographic structure and dynamics of poultry kept in small-scale farms of the Mekong river delta region.

## Methods

### Data collection

A longitudinal observational study of small-scale poultry farms was conducted in Ca Mau province located at the southern tip of Vietnam. Ca Mau province is part of the Mekong river delta. The province reported 78 AI outbreaks in domestic poultry over the period 2006–2015 (communication of the Department of Animal Health of Vietnam). Results of sampling and molecular diagnosis performed in rural live bird markets by the Ca Mau sub-Department of Livestock Production and Animal Health (CM-LPAH) in 2015 “indicated that prevalence of the H5 influenza subtype in individual birds is likely between 4.2% and 6.3%” in the domestic birds of the province (CM-LPAH reports and [24]).

The sampled farms were in two rural communes of the Ca Mau province (25 farms in each commune), selected for “their past history of avian influenza outbreaks, high farm density, expected participation rate, and proximity to the main city in the province” [24] The most common poultry breeds found in these communes are “gà nòi lai” for chickens (a crossbreed between local meat breeds and fighting breeds) and “vịt siêu thịt” for ducks (a crossbreed between the Cherry Valley breed originating from the United Kingdom and local breeds). The study was carried out with the support and collaboration of the CM-LPAH. The collaboration between the investigators (authors) and CM-LPAH was approved by The Hospital for Tropical Diseases in Ho Chi Minh City, Vietnam. The CM-LPAH specifically approved this study; at the province-level in Vietnam, CM-LPAH is the equivalent of an Animal Care and Use Committee that approves studies involving biological sampling from animals.

**Fig. 1:**
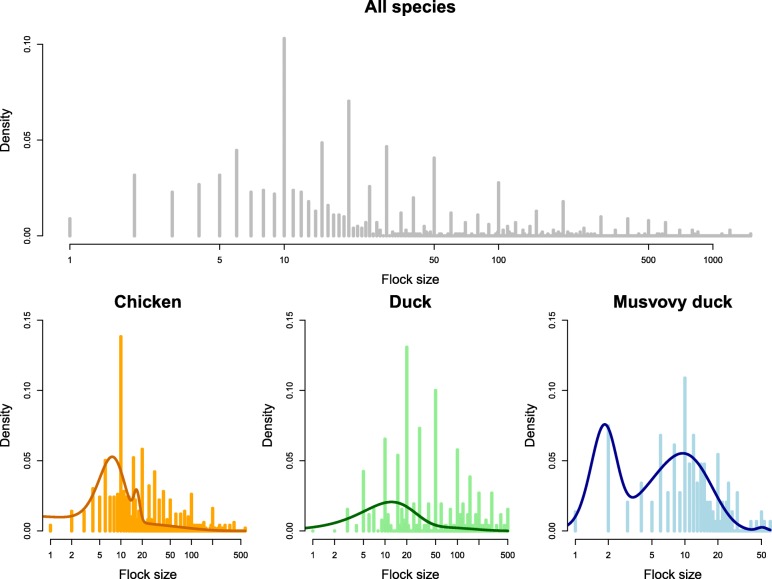
Histograms of the sizes of poultry flocks in the study farms. Top: all species aggregated; bottom: plotted by species. Solid lines represent best-fit distributions (mixture of two gamma components (ducks) or three gamma components (chickens and Muscovy ducks))

As described in a previous publication [24], 50 smallholder poultry farms were initially enrolled. The selection of participating farms followed a convenience sampling scheme and all initially contacted farmers accepted to participate in the study. Three additional farms were enrolled during the course of the study after three farms discontinued participation (one farmer moved to another province and the two others stopped poultry production). These three farms were also selected based on convenience and met the same criteria as the initially enrolled farms. Inclusion criteria for study participation were based on farm size; farms with a total poultry count between 20 and 100 were considered small, and farms holding more than 100 birds were considered large. For chicken farms, farm selection was carried out to enroll 80% small and 20% large farms, while for duck farms the goal was to have an equal split between small and large farms. This sampling objective is based on the known difference in farm size distribution between species in Vietnam, duck farms having a larger average size than chicken farms [27]. During enrollment it was apparent that the vast majority of “duck farms” also housed chickens, therefore many enrolled farms were classified as having both ducks and chickens. Enrollment counts for farm types were 11 chicken, 13 both, and 1 duck farm in Tan Loc commune; and 6 chicken, 15 both, and 4 duck farms in Tan Phu commune. Final flock sizes and flocks per farm can be seen in Fig. 1 and the Additional file 3.

Study duration was 20 months, from June 2015 to January 2017. A Vietnamese-language farm questionnaire specifically developed for this study was collected at the end of each study month. Questionnaires are available in Vietnamese and English language in Additional file 1. The collected information included: number of birds of each species present on the farm and their production type (broiler or layer/breeder); expected age of removal from the farm; number of birds introduced, removed and deceased in the last month with, in case of death, associated cause and/or clinical symptoms; vaccines administered to birds with date of vaccination; type of housing (indoor, outside in pens or free-grazing) and disease prevention practices (disinfection, avoidance of contact with people from outside and change of boots when entering farming facilities) applied in managing the birds. To facilitate data collection, each farm’s poultry were classified into groups (hereafter referred to as “flocks”) with the same age, species, and production type, and data were organized at the flock level rather than at the farm level; the flock is the natural unit of management for a Vietnamese smallholder poultry farmer. Birds were classified into three classes according to their age and production type: young (chicks or ducklings, < 1 month old), broiler (> 1 month old, grown to be slaughtered for meat production), and layer/breeders (> 1 month old, kept for egg production and/or reproduction). Broilers and layers did not have any age limit and broilers could become layer in the course of their production period if farmers decided to use them for producing eggs or for breeding.

### Data processing

The questionnaire data were input and stored in a SQLite relational database. To convert the cross-sectional dataset into a longitudinal dataset, the flocks labeled in monthly questionnaires needed to be identified as being the same flock or not (e.g. flock #3 in March with 40 2-month old chickens and flock #7 in April that had 40 3-month old chickens would be identified as the same flock). To perform this “flock matching”, data on flock age, species, production type, inflows (buying and hatching), and outflows (selling, slaughtering, and other causes of death) were used, ensuring that other flock characteristics were as similar as possible. These matches were performed on a per-farm basis, looking at two consecutive monthly questionnaires at a time.

With approximately 1000 questionnaires to process, a flock-matching algorithm was developed to process the data, and consistency and correctness were checked manually. From one month to the next, each flock must have had at most one match, but flocks can also appear (buying and hatching) or disappear (selling, slaughtering). The algorithm was developed so that the state of a flock would correspond as much as possible to the expected state of its match in the previous month. This problem was a variant of an assignment problem for bipartite graphs, the goal being to minimize an objective function, for any farm at any month:

$$ \sum \limits_{a\epsilon A}\sum \limits_{b\epsilon B}C\left(a,b\right){x}_{a,b}+\sum \limits_{a\epsilon A}N(a){x}_a^N+\sum \limits_{b\epsilon B}D(b){x}_b^D $$

where *A* is the set of flocks at month *i, B* is the set of flocks on the same farm at month *i-1. x*_*a*, *b*_ is 1 if the flock *a* is paired with the flock *b*, and 0 otherwise. *C*(*a*, *b*) is the cost of pairing *a* to *b*, (see Additional file 2). *N(a)* is the cost of making *a* a new flock (${x}_a^N=1$), and *D(b)* the cost of making *b* a removed flock (${x}_b^D=1$). The objective function must be minimized under a set of constraints ensuring that each flock has at most one match:

$$ \sum \limits_{b\epsilon B}{x}_{a,b}+{x}_a^N=1,\forall a\in A $$

$$ \sum \limits_{a\epsilon A}{x}_{a,b}+{x}_b^D=1,\forall b\in B $$

The algorithm is implemented in Python (v3.0), and is available online [28]. More details on the algorithm and the cost functions used are available in the Additional file 2.

### Data analysis

Rates of poultry removal and death were estimated. As the number of deaths and removals were collected on a monthly basis, the exact numbers of birds introduced, removed, and deceased on each day were not available. Thus, the exact day of introduction/removal/death was imputed assuming a uniform probability of this event occurring at any day during the month, and 10,000 imputed data sets (with exact days of introduction/removal/death events) were simulated to provide estimates and ranges of death rates and removal rates in the poultry population. Different probability distributions (exponential and mixtures of one, two, and three gamma distributions) were fit to the distributions of flock sizes. The size of a flock size is defined as its maximum size during its existence, i.e. the total number of birds being present in the flock for at least some time (almost always at the beginning of the flock cycle). Best fits were determined through maximum-likelihood estimation, and in the case of mixtures, using the expectation maximization algorithm of the mixtool R package [29]. Best fits were chosen according to Akaike Information Criterion (AIC). The association between time series of median estimates of disease-associated mortality rate and bird population size, fraction of birds farmed outdoor, fraction of birds farmed without disinfection and fraction of birds being in contact with people from outside the farm were assessed using Kendall’s rank correlation coefficient. All data analyses were performed using R version 3.3 [30].

## Results

From June 2015 to January 2017 (20 months), a total of 53 small scale poultry farms were monitored (26 in Tan Loc commune and 27 in Tan Phu commune). 47 farms were monitored for 20 months and 6 were monitored for 2 to 17 months, returning a total of 976 farm-months. The questionnaire data described 1087 discrete poultry flocks comprising 110,232 birds: 48,356 chickens, 33,570 quails, 25,450 ducks, 2443 Muscovy ducks, 195 geese, 183 pheasants, and 35 pigeons. Chickens, ducks, and Muscovy ducks (MD) were the most common species on the farms, and these are known to be the three common types of poultry raised, sold, and eaten in southern Vietnam [31]; quails were present on four study farms. Flock timelines are shown in Additional file 3 (plots per farm) and Additional file 4 (plots per commune). Only one of the monitored farms implemented all-in-all-out management throughout the study period. There was substantial flock overlap on all other farms. Farmers kept poultry mainly for earning an income and each farm was managed by a single family. Duck farms were stationary, all farmers remained in the same commune across the whole study period while occasionally conducting their ducks on water bodies located at varying distance to the farm. Other farm activities included rice cropping and python farming.

### Distribution of flock sizes and species

The distribution of the number of chickens and ducks per flock was highly right-skewed and over-dispersed (Fig. 1); the mean flock sizes were 40 (chicken), 81 (duck) and 14 (MD) while the median flock sizes were 16 for chickens (inter-quartile range (IQR): 10–40), 32 for ducks (IQR: 18–101) and 11 for Muscovy ducks (IQR: 7–18). A mixture of two or three gamma components gave the best fit for the distribution of flock sizes (three components for chickens and Muscovy ducks, two components for ducks) (Additional file 5).

Only four farmers kept a single species of poultry over the whole study period (two were chicken farmers and two were duck farmers). Out of 976 farm-months 32% had only one species of bird, 33% had two different species, and 35% had at least three different species. The probability that a flock with birds of a given species would be present in the same farm as a flock with birds of each of the other species (with a subsequent risk of interspecific contact) is shown in Table 1. This probability was especially high among the three most common species. In farms combining at least two of the three main species, the correlation between the numbers of birds of each pair of species was assessed using Kendall’s rank correlation coefficient. The number of ducks was positively correlated with the number of MDs (*τ* = 0.14, *p*-value < 0.01) and chickens (*τ* = 0.07, *p*-value =0.042). The correlation between the number of chickens and MDs was not significant (*p*-value =0.62).

**Table 1:** Interspecific interaction matrix on poultry farms. The probability that a flock with birds of one species (row) will be in present in the same farm as a flock with birds of another species (column)

	Chicken	Duck	Muscovy duck	Goose	Pheasant	Quail	Pigeon
Chicken	1	0.45	0.41	0.23	0.06	0.04	0.05
Duck	**0.81**	1	**0.51**	0.27	0.04	0.04	0.06
Muscovy duck	**0.9**	**0.62**	1	0.29	0.04	0.03	0.09
Goose	**0.92**	**0.6**	**0.54**	1	0.14	0.05	0.15
Pheasant	**0.98**	0.35	0.31	**0.55**	1	0	0.09
Quail	**0.89**	**0.57**	0.29	0.31	0	1	0
Pigeon	**0.82**	**0.57**	**0.71**	**0.63**	0.1	0	1

Probabilities higher than 50% are in boldface

### Life cycle of poultry

**Fig. 2:**
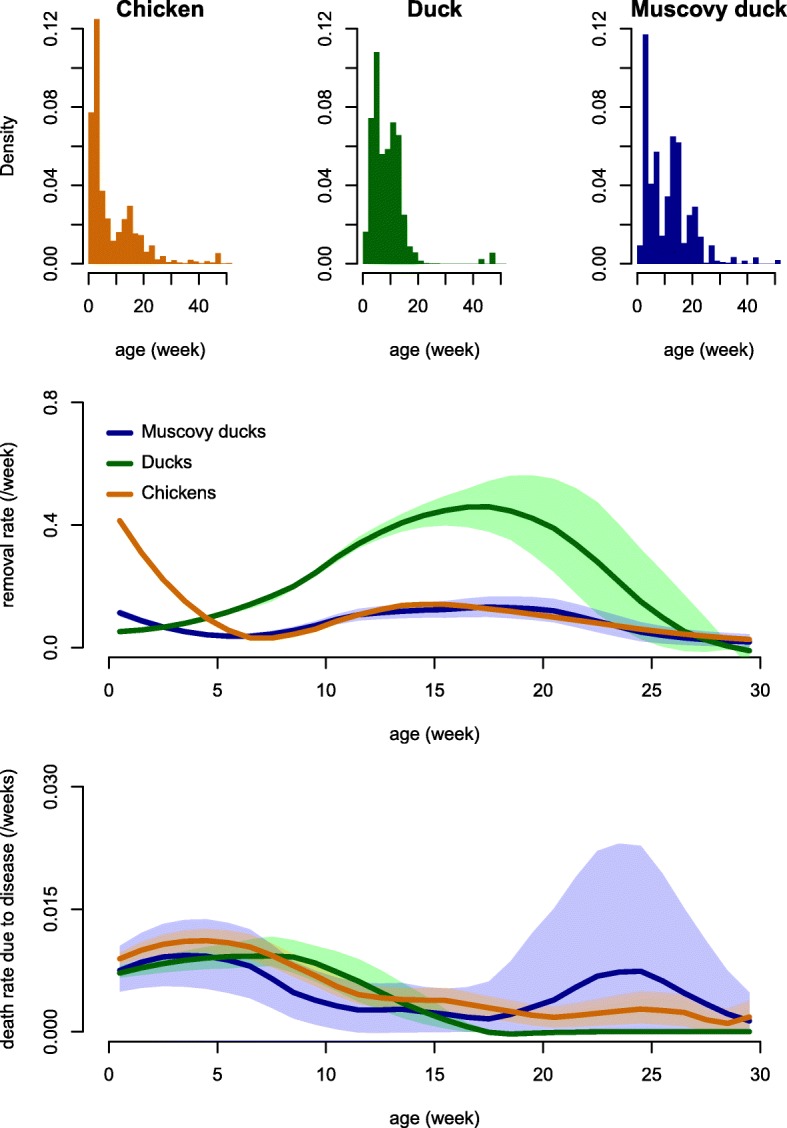
Distribution of age-at-departure and age-specific removal and mortality rates in the populations of chickens, ducks and Muscovy ducks of the study farms during the 20-month study period. Top: distribution of ages at departure (death or removal). Middle: rate of removal (sale, gift, or home slaughter) as a function of age. Bottom: disease-related death rate as a function of age. Shaded regions in the middle and bottom graphs show the 95% range using imputed values; solid lines show the median. The time series of median, 2.5, and 97.5% quantiles were smoothed using local regression (span factor: 0.5)

Distribution of age of departure from the farms and simulated distributions (from imputed data) of rates of removal and death due to disease according to poultry age in the three main poultry species are shown in Fig. 2.

**Fig. 3:**
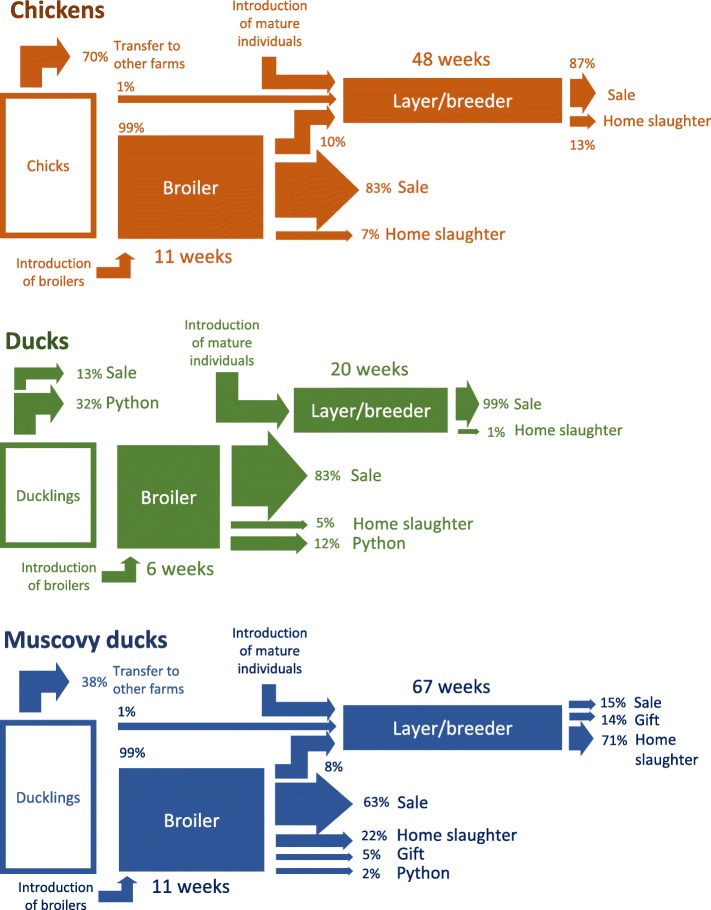
Flow diagram representation of the life history of chickens, ducks, and Muscovy ducks present in the study farms during the 20-month study period. The diagrams show the average duration of the production period in the broiler and layer-breeder classes and the proportion of poultry used for different purposes at the end of their production period

The age-specific removal rate was consistently higher for ducks than for chickens or MDs, except during the first 3 weeks, while the removal rate of MDs was the lowest (Fig. 2, middle). Results showed 70% of young chickens, 45% of young ducks and 38% of young MDs were removed from the farm before reaching their first month. The high removal rate of young chickens, and to a lesser extent of MDs, was partly attributable to the presence of poultry farms with a high breeding activity (breeding and sale of young birds to be grown on other farms), while most sold young ducks were used to feed pythons. This explains the two clusters of age at departure in chickens, one corresponding to sale of newborn chicks, the other to the sale of mature broilers (Fig. 2, top). For all three species, more than 90% of adult broilers came from the young stock of the farm. On average broiler chickens and MDs were kept about twice as long as ducks (Fig. 3): the removal rates were 0.075/week (95% confidence interval (CI): 0.074–0.076), 0.073/week (95% CI: 0.069–0.077), and 0.146/week (95% CI: 0.144–0.148) in chickens, MD, and ducks respectively. The average age at removal (sale, gift, home slaughter, or python feeding) for broilers was 15.5 weeks, 9.8 weeks and 14.7 weeks in chickens, MD, and ducks respectively. The same difference of removal rate was observed in layers: the removal rate was 0.021/week (95% CI: 0.02–0.022), 0.015/week (95% CI: 0.012–0.018), and 0.051/week (95% CI: 0.049–0.054) in chickens, MD, and ducks respectively.

Layer/breeder poultry were much older than broilers (average age above one year in the three main species). The ratio of layers/breeders to broilers was twice as high in chickens and MDs (0.41 in both species) than in ducks (0.20). In addition, the ratio of layer/breeder per introduction (addition of a new bird on a farm) was lower in ducks (0.19) than in chickens (0.37) and MDs (0.66). All layer/breeder ducks were introduced from another farm at a mature age, there was no sourcing of layer ducks from the young or broiler stock of the same farm. 63% of layer/breeder chickens and 68% of layer/breeder MDs were previously raised on the same farm, most of them being first part of a broiler flock and later kept for egg production or breeding instead of being sold or slaughtered (Fig. 3).

The difference in the modality of supply of layer/breeder individuals was translated in the distribution of age at departure. In ducks, most individuals departed before 20 weeks of age (broilers), and all others after 40 weeks (layer/breeders), while in the two other species some birds did depart between 20 and 40 weeks (Fig. 2). The relatively high removal rate of chicks and relatively short production period of adult ducks translated into a higher rate of bird introduction (number of introductions of new individuals - newborn or purchased young and adults - per month divided by the population size) which was twice as high in chickens and ducks (0.66 and 0.62, respectively) as in MDs (0.36).

### Poultry mortality

A total of 8.2% of birds died during the course of the study instead of being sold, offered as gifts, slaughtered, or fed to pythons, and 44% of these deaths were associated with a disease state (observation of clinical symptoms by farmers, diagnosis of a specific disease by farmers, or sudden death of several birds with no obvious cause). Other causes of death were accidents (disappearance of birds during grazing period, injuries from fighting, or attacks by rats or snakes) (15% of all birds), cold temperatures (5% of all birds), and unknown causes (36%). The average mortality proportion per flock was 19.9%, 60% of which was disease-induced. Note that these figures probably underestimate the true amount of loss due to disease since a few farmers explicitly mentioned they fed their sick poultry to pythons instead of letting them die. The average monthly per-farm risk of facing a disease-induced mortality rate higher than 20% was 4.4%. Such events are indicated on the farm timelines in Additional file 3.

**Fig. 4:**
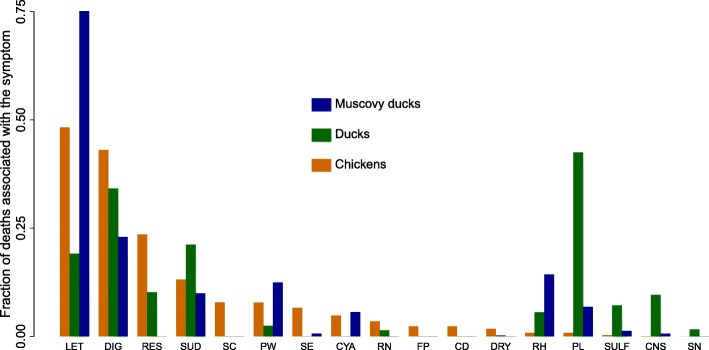
Frequency of association between disease-induced mortality and reported symptoms or reported suspected causes, based on all symptoms or causes listed for each reported death event in the populations of chickens, ducks, and Muscovy ducks of the study farms during the 20-month study period. E.g., for 75% of Muscovy duck disease-induced deaths, lethargy was reported. LET: lethargy, weariness; DIG: digestive symptom (diarrhea, flatulence or abnormal color of feces); RES: symptoms related to the lower respiratory tract (dyspnea or amplified respiratory sounds); SUD: sudden death (birds died before any symptoms could be noticed); SC: swollen crop; PW: paralyzed wing; SE: anorexia; CYA: cyanosis; RN: symptoms related to the upper respiratory tract (runny nose); FP: fowl pox; CD: coccidiosis; DRY: dry legs; RH: retraction of the head; PL: paralyzed leg; SULF: intoxication with sulfate; CNS: nervous-system symptoms; SN: shrinking neck

Frequencies of observation of clinical symptoms when disease-related deaths occurred are shown in Fig. 4. Poultry deaths were often associated with lethargy/weariness and digestive symptoms in the three species. Respiratory symptoms were specifically often reported in chickens while leg paralysis and nervous-system symptoms were quite specific to ducks. The mortality rate attributable to disease appears to be a decreasing function of age (Fig. 2), approximately 1% per week in birds below 5 weeks of age and decreasing progressively afterwards. In MDs, however, an increase in mortality rate attributable to disease was observed around 20 weeks of age.

### Housing and grazing of poultry and infectious disease prevention

**Fig. 5:**
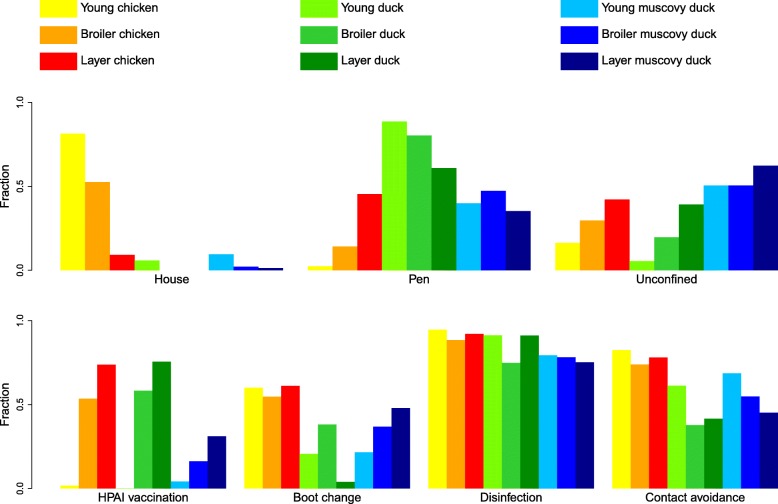
Fraction (proportion of animal-months) of poultry in a particular housing type (top) and undergoing particular prevention practices against infectious diseases (bottom) in the populations of chickens, ducks, and Muscovy ducks of the study farms over the 20-month study period

Approximately half of chickens were housed indoors while the overwhelming majority of ducks and MDs were farmed outdoors, either in pens (i.e. outdoor enclosures), which was most common for ducks, or free-grazing (i.e. unconfined and wandering freely in the farm or in the neighborhood), which was most common for MDs (Fig. 5). Most ducks (69%) grazed in water bodies during a part of the day (some of them being kept in pens at night). The average grazing distance for ducks (kept outdoor or partly penned) was 108 m away from the farm and the maximum distance was 1.5 km. While most young and broiler chickens were housed indoors, layer-breeder chickens were mainly kept outdoors (either unconfined or in pens).

In chickens, the vaccination coverage was 50% for AI and 26% for Newcastle Disease. In ducks the vaccination coverage was 44% for AI and 1.5% for duck plague. In MDs the vaccination coverage was 17% for AI. The other diseases against which vaccination was practiced were Fowl cholera (in chickens and Muscovy ducks) and Gumboro disease (in chickens). Older birds have a higher chance of having received a vaccination during their lifetime, therefore in the three species the proportion of vaccinated birds was logically highest in layer-breeders, who are older than average, slightly lower in broilers, and lowest in newborns (Fig. 5).

In the three species disinfection was the most commonly applied infectious disease prevention practice (> 70% of birds). In comparison, avoidance of contact with visitors was less common and boot changing when entering the farm was even rarer, especially in ducks and MDs (< 40% of birds).

### Temporal dynamics of population and mortality

**Fig. 6:**
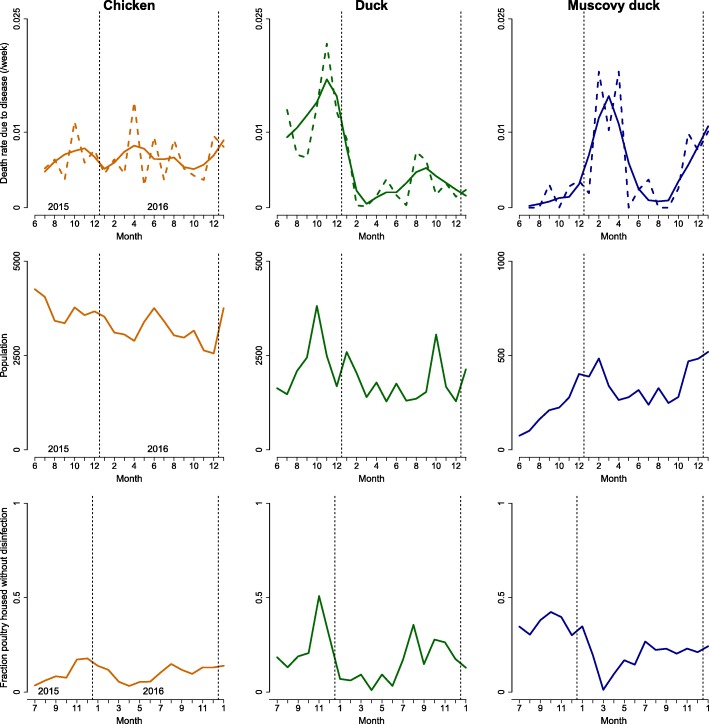
Time series of disease-induced mortality rate, population size, and absence of disinfection over the study period in the populations of chickens, ducks, and Muscovy ducks of the study farms over the 20-month study periods (from June 2015 to January 2017). Top: Dashed lines are median estimates of death rates (from imputation) while solid lines are smoothed rates obtained through local regression (span factor: 0.5)

Time series of population size and mortality rate attributable to disease are shown in Fig. 6. Chicken population size slightly decreased during the study period and was not obviously seasonal. Duck population size peaked in October while the population of MDs increased at the end of each calendar year.

In chickens, the rate of mortality attributable to disease did not vary much across the study period, except for a peak of deaths mostly associated with digestive symptoms in April 2016. In ducks, two peaks of mortality attributable to disease occurred during the study period, the first mostly associated with lethargy and the second with digestive symptoms. In MDs two peaks in disease-related mortality (from less than 0.1%/week to more than 1%/week) were observed in early and late 2016, both mostly associated with lethargy in birds. Both increases seemed to follow a peak in the MDs’ population size. Overall, the reported disease symptoms were too non-specific to link the peaks of mortality to a specific pathogen and no significant correlation was observed between the time series of frequency of disease symptoms and disease mortality rate.

Time series of median estimates of disease-associated mortality rate and population size at the beginning of the month were significantly positively correlated for Muscovy ducks (Kendall’s coefficient τ = 0.49, *p*-value < 0.01). In chickens and ducks the correlation was not significant (*p*-values of 0.63 and 0.53, respectively). In ducks the time series of median estimate of disease-associated mortality rate had a positive significant association with the fraction of unconfined ducks (grazing outdoor) (τ = 0.33, *p*-value = 0.05), the fraction of ducks farmed without disinfection (τ = 0.4, *p*-value = 0.02) and the fraction of ducks in contacts with people from outside the farm (τ = 0.35, *p*-value = 0.04), these three variables being highly correlated and most likely indicative of periods when ducks graze on rice fields, thus increasing their probability of contacts with outsiders. This correlation was not observed in the two other species. No significant correlation was observed between the fraction of birds vaccinated against AI and the mortality rate in the three species. These patterns of association are illustrated in Fig. 6.

## Discussion

Assessments of the demographic structure and dynamics of small-scale poultry farms are made difficult by the lack of systematic accounting of birds’ entries and exits on farms, an absence of all-in-all-out management, simultaneous presence of birds of different species and production stages on the same farm, and combination of self-renewal and outsourcing of young birds from other farms and/or hatcheries. Longitudinal surveys, such as the one conducted in this study, are therefore necessary. One difficulty in such longitudinal surveys is the tracing of each identified birds from one month to the next. This difficulty was overcome through a flock-matching algorithm. The study data combined with the flock-matching algorithm produced an accurate description of smallholder farms’ demographic structure and dynamics in the Mekong river delta region.

All-in-all-out flock management was nearly absent and multispecies farming was widespread. Farmers usually kept at least two different species of birds, and chickens had an almost 50% chance to be farmed together with ducks. The on-farm number of ducks was positively correlated with the number of Muscovy ducks and chickens. In other studies, multispecies poultry farms have been shown to be at higher risk of introduction of AI [22, 32]. However, in the context of small-scale farming, with highly fluctuating input and output market prices and absent or limited risk-mitigation mechanisms (absence of contracts with suppliers or buyers, insurance schemes or vertical integration) [2, 8, 12, 25], diversification of livestock production constitutes a way of limiting income variation over time [33]. In addition, duck farming is deeply integrated in the rice production systems of the Mekong region since scavenging ducks feed on grains left in freshly harvested rice fields and, at the same time, reduce the density of parasitic snails [11, 34].

Farmers lost on average 11.9% of their production to disease, representing 3.6% disease-induced mortality in the whole population. In Muscovy ducks, major peaks of disease-related mortality closely followed peaks in population size. In ducks these peaks were more likely related to variations in farming conditions, with periods of increased outdoor grazing being more at risk. The chicken population size and disease-attributed mortality had less temporal variation and did not appear to correlate temporally with each other. The observations for Muscovy ducks are consistent with the hypothesis that the transmission of infectious diseases in smallholder domestic poultry farms has a density-dependent component and, therefore, that any increase in the domestic bird population puts farms at higher risk of disease-induced losses [35, 36]. These variations in poultry population can be driven by changes in demand for poultry products, as occurs during the Lunar New Year festival [37]. The lack of observed seasonality in the chicken population is surprising, considering the observed increase in demand for chicken meat at Lunar New Year. Nonetheless this increase in demand is reportedly less pronounced in the southernmost provinces of Vietnam [37]. In the case of AI, it was shown that outdoor scavenging of ducks on rice fields or water bodies puts them at risk of disease transmission from other domestic duck flocks or wild birds, as was demonstrated for AI [23]. The temporal fluctuation in the outdoor grazing activity of ducks is related to rice production cycles which are quite specific to each area of the Mekong river delta region [11].

This study highlights some species-specific farm characteristics that may influence the epidemiology of infectious diseases in poultry. Muscovy ducks are kept outdoors and are mostly free-grazing, which favors contacts with infectious pathogens excreted by wild birds and poultry of neighboring farms [23] and their vaccination coverage is very low. MDs may be more at risk of contact with pathogens released in the environment or with infectious birds sharing the same scavenging area and the epidemiology of disease transmission in MDs is likely to be strongly influenced by the individual farm that they are housed on. Chickens and ducks, on the other hand, show a markedly higher rate of removal and introduction of birds. This is due both to the outsourcing of chicks from farms specialized in breeding and, in the case of ducks, to the shorter production period of birds. It is known from other studies that small-scale poultry farmers who do not practice self-renewal usually purchase their chicks and ducklings directly from local breeding farms and hatcheries but also from chick/duckling assemblers and distributors or at the local market. The origin and health status of these purchased young birds is poorly controlled [8, 31]. The majority of chicken and ducks sold by small-scale farms are handled by local traders and wholesalers who gather poultry from a large number of farms in different areas. Birds usually are stored and sold in live-bird markets where contacts with a large number of birds of different origins create suitable conditions for pathogen circulation [8, 25, 26]. Thus, frequent visits to poultry farms by itinerant poultry traders and purchase of chicks and duckling may increase the risk of pathogen introduction in the farm. Chicken and duck farms may be more at risk of introduction of pathogens through bird movements, and the epidemiology of avian influenza transmission in chickens and ducks may be more strongly influenced by the trading network than by individual farm characteristics.

The results highlight different modalities of renewal of the poultry stock in the three species. In chickens, a substantial number of smallholder farms bred and supplied other smallholder farms with chicks, which explains the high removal rate of chickens in their two first weeks of life (> 40%/week). Since the origin of the young birds (bred and hatched in the farm or supplied from other farms or hatcheries) was not informed in the questionnaires, it is unclear to which extent the studied poultry farms practice self-renewal or outsource their young stock from outside. However, it is evident that the sale of ducklings to supply other smallholder farms is more limited. The low ratio of layer/breeder per poultry introduction in ducks tends to suggest that a significant fraction of ducklings are purchased from other types of suppliers (large breeding farms and hatcheries). Conversely, the high ratio of layer/breeders per poultry introduction in MDs tends to suggest MD farms maintain their population essentially through self-renewal (birds are bred, hatched and grown on the same farm).

The three main poultry species have markedly different turnovers. For ducks, the production period of both broiler and layers is short. In addition, no layer ducks are bred and raised on the same farm and broilers are never converted to layer/breeders resulting in a substantially shorter life span of ducks. Consequently, to maintain a given level of herd immunity (a given proportion of immunized birds) against contagious diseases such as AI, a frequency of vaccination at least twice as high would be needed on duck farms as compared with chickens and MD since the rate of removal of immunized birds and introduction of susceptible birds is twice as high. This observation is important, considering the major role played by ducks, and particularly broiler ducks in the epidemiology of AI in the Mekong river delta [32, 38]. The average length of the production period of duck and chicken broilers is consistent with other reports on smallholder poultry systems in Vietnam [31]. The shorter production period of ducks can be explained by a higher production efficiency and specialization of the used breeds, shortening their growth period, and increasing the outsourcing of broilers and layers from breeding farms. According to previous studies, duck farms seem to use a high proportion of exotic breeds (i.e. imported breeds selected for increased production efficiency) while small chicken farms tend to use preferentially indigenous breeds, which are more appealing to Vietnamese consumers [6, 10, 31, 39]. Most produced broiler domestic birds of the three species were traded and a smaller fraction were slaughtered at home or, for Muscovy ducks, given to neighbors and relatives. This shows the commercial orientation of the small-scale poultry farms. About 40% of ducks were fed to pythons (32% of ducklings and 12% of broilers). Python production is common in the Mekong river delta and the protein produced by the poultry industry was mentioned as an important source of feed for the farmed pythons [40].

Our study has a few key limitations. The reported clinical symptoms associated with bird mortality were mostly non-specific and could not be linked with a precise disease. We were therefore unable to link peaks in mortality to a specific poultry pathogen. Although the two selected communes are representative of the agricultural production systems of Ca Mau province, our sample of farms probably does not capture the extent of the diversity of small-scale poultry farming systems of the Mekong river delta region. In particular, demographic characteristics such as bird turnover and mortality may depend on the used poultry breeds (which affects the body growth curve and the susceptibility to infectious disease) which might differ from one area to the other. As this survey focused on intra-farm poultry population dynamics, no information was gathered on the commercial circuits in which poultry were sold and traded, and their implication for pathogen dissemination. Moreover, no individual data were collected on the other sources of income of participating farmers although it can be hypothesized that the diversification of economic activities (agricultural or not) by farmers may affect their farm management and likelihood of adopting certain disease prevention practices.

## Conclusion

Control of avian influenza in Asia will likely continue employing poultry vaccination, responsive depopulation, and basic farm management/biosecurity implementation tools as its main methods. A better understanding of poultry turnover rates, species overlap, flock overlap, species-specific infections risks, and the influence of environmental transmission and trader/market based transmission will be critical for designing control and response policies that can be tailored to regions with known poultry species compositions and known farm management practices. Our results show that the rate of interspecific contact and overlap between flocks of different ages is high in small-scale poultry farms. Some at-risk farm management practices are species-specific. Muscovy ducks are farmed outdoors with little disease prevention but also fewer inter-farm movements. Ducks and chickens are more at-risk of introduction of pathogens through inter-farm movements of birds. Ducks are farmed in large flocks with high turnover and, as a result, are more vulnerable to disease spread and require higher vaccination frequency to maintain herd immunity. Increases in population size (for MDs) and increased outdoor grazing (for ducks) seem to be the main drivers of the dynamics of mortality due to disease.

## Additional files

Additional file 1: Questionnaires in English and Vietnamese version. (PDF 747 kb)

Additional file 2: Description of the flock-matching algorithm. (PDF 1246 kb)

Additional file 3: Timeline of poultry flocks in each study farm. (PDF 559 kb)

Additional file 4: Timeline of poultry flocks in each study commune. (PDF 276 kb)

Additional file 5: Characteristics of fitted probability distributions of flock sizes of the three main poultry species. (PDF 176 kb)

## Data Availability

The raw data collected during the present study are available from the corresponding author upon request.

## Abbreviations

AI: Avian Influenza
AIC: Akaike Information Criterion
CM-LPAH: Ca Mau sub-Department of Livestock Production and Animal Health
MD: Muscovy Ducks

## References

[1] General Statistics Office of Vietnam (2017). Results of the 2016 Rural, Agriculture and Fishery Census.

[2] Burgos S, Hinrichs S, Otte J, Pfeiffer D, Roland-Holst D (2008). Poultry, HPAI and Livelihoods in Viet Nam - A Review.

[3] General Statistics Office of Vietnam (2017). Statistical Yearbook of Vietnam 2016.

[4] Hong Hanh PT, Burgos S, Roland-Holst D (2007). The Poultry Sector in Viet Nam: Prospects for Smallholder Producers in the Aftermath of the HPAI Crisis. Pro-Poor Livestock Policy Initiative Research Report.

[5] Ifft J, Roland-Holst D, Zilberman D (2012). Consumer valuation of safety-labeled free-range chicken: results of a field experiment in Hanoi. Agric Econ.

[6] Ifft J, Roland-Holst D, Zilberman D (2009). Impact of quality characteristics on demand for chicken in Viet Nam. Agr Resour Econ Update.

[7] Epprecht M. Geographic Dimensions of Livestock Holdings in Vietnam. Spatial Relationships among Poverty, Infrastructure and the Environment. Pro-Poor Livestock Policy Initiative, editor. Hanoi, Vietnam: Ministry of Agriculture and Rural Development of Vietnam; 2005.

[8] ACI (2006). Poultry Sector Rehabilitation Project – Phase I: The Impact of Avian Influenza on Poultry Sector Restructuring and its Socio-economic Effects. Prepared for the Food and Agriculture Organization of the United Nations.

[9] VSF - Vétérinaires Sans Frontières (2006). Review of Free-Range Duck Farming Systems in Northern Viet Nam and Assessment of their Implication in the Spreading of the Highly Pathogenic (H5N1) Strain of Avian Influenza (HPAI).

[10] Henning J, Henning KA, Long NT, Ha NT, Vu le T, Meers J (2013). Characteristics of two duck farming systems in the Mekong Delta of Viet Nam: stationary flocks and moving flocks, and their potential relevance to the spread of highly pathogenic avian influenza. Trop Anim Health Prod.

[11] Minh PQ, Stevenson MA, Schauer B, Morris RS, Quy TD (2010). A description of the management of itinerant grazing ducks in the Mekong River Delta of Vietnam. Prev Vet Med.

[12] Delabouglise A, Antoine-Moussiaux N, Phan TD, Dao DC, Nguyen TT, Truong BD (2016). The perceived value of passive Animal health surveillance: the case of highly pathogenic avian influenza in Vietnam. Zoonoses Public Health.

[13] OIE (2018). World Animal Health Information Database (WAHIS Interface).

[14] Choi K-S, Kye S-J, Kim J-Y, Nguyen DT, Lee Y-J, To TL (2013). Molecular epidemiology of Newcastle disease viruses in Vietnam. Trop Anim Health Prod.

[15] Guan Y, Poon LLM, Cheung CY, Ellis TM, Lim W, Lipatov AS (2004). H5N1 influenza: a protean pandemic threat. Proc Natl Acad Sci U S A.

[16] Boni MF, Nguyen TD, de Jong MD, van Doorn HR (2013). Virulence attenuation during an influenza a/H5N1 pandemic. Philos Trans R Soc Lond Ser B Biol Sci.

[17] Gilbert M, Xiao X, Pfeiffer DU, Epprecht M, Boles S, Czarnecki C (2008). Mapping H5N1 highly pathogenic avian influenza risk in Southeast Asia. Proc Natl Acad Sci U S A.

[18] Henning Joerg, Pfeiffer Dirk U., Vu Le Tri (2008). Risk factors and characteristics of H5N1 Highly Pathogenic Avian Influenza (HPAI) post-vaccination outbreaks. Veterinary Research.

[19] Pfeiffer DU, Minh PQ, Martin V, Epprecht M, Otte MJ (2007). An analysis of the spatial and temporal patterns of highly pathogenic avian influenza occurrence in Vietnam using national surveillance data. Vet J.

[20] Sims LD (2007). Lessons learned from Asian H5N1 outbreak control. Avian Dis.

[21] FAO (2011). Approaches to controlling, preventing and eliminating H5N1 highly pathogenic avian influenza in endemic countries.

[22] Desvaux S, Grosbois V, Pham TTH, Fenwick S, Tollis S, Pham NH (2011). Risk factors of highly pathogenic avian influenza H5N1 occurrence at the village and farm levels in the red River Delta region in Vietnam. Transbound Emerg Dis.

[23] Henning KA, Henning J, Morton J, Long NT, Ha NT, Meers J (2009). Farm- and flock-level risk factors associated with highly pathogenic avian influenza outbreaks on small holder duck and chicken farms in the Mekong Delta of Viet Nam. Prev Vet Med..

[24] Thanh NTL, Vy NHT, Xuyen HTA, Phuong HT, Tuyet PN, Huy NT, Nguyen-Van-Yen B, Lam HM, Boni MF. No Evidence of On-farm Circulation of Avian Influenza H5 Subtype in Ca Mau Province, Southern Vietnam, March 2016 – January 2017. PLOS Currents Outbreaks. 2017 May 5. Edition 1. 10.1371/currents.outbreaks.c816d7333370d68f8a0da33f69168986. 10.1371/currents.outbreaks.c816d7333370d68f8a0da33f69168986 PMC5501696 28736677

[25] Tung DX, Costales A (2007). Market participation of smallholder Poultry producers in northern Viet Nam.

[26] Fournie G, Guitian J, Desvaux S, Mangtani P, Ly S, Vu CC (2012). Identifying live bird markets with the potential to act as reservoirs of avian influenza a (H5N1) virus: a survey in northern Viet Nam and Cambodia. PLoS One.

[27] General Statistics Office of Vietnam (2007). Results of the 2006 Rural, Agriculture and Fishery Census.

[28] Nguyen Van Yen B. Flock matching algorithm repository. 2017; Available from: https://gitlab.com/bnguyenvanyen/Poultry-farms/blob/master/poul/flockmatch.py. Accessed 31 May 2018.

[29] Benaglia T, Chauveau D, Hunter DR, Young D (2009). Mixtools: an R package for analyzing finite mixture models. J Stat Softw.

[30] R core team. R: a language and environment for statistical computing. Vienna, Austria: The R foundation; 2014; Available from: http://www.R-project.org/. Accessed 8 Oct 2012.

[31] Desvaux S, Ton VD, Thang PD, Hoa PTT (2008). A general review and a description of poultry production in Vietnam.

[32] Henning J, Henning KA, Morton JM, Long NT, Ha NT, Vu LT (2011). Highly pathogenic avian influenza (H5N1) in ducks and in-contact chickens in backyard and smallholder commercial duck farms in Viet Nam. Prev Vet Med..

[33] Roland-Holst D, Epprecht M, Otte J (2007). External shocks, producer risk, and adjustment in smallholder livestock production: the case of HPAI in Viet Nam.

[34] Liang K, Zhang J, Song C, Luo M, Zhao B, Quan G (2014). Integrated management to control Golden apple snails (Pomacea canaliculata) in direct seeding Rice fields: an approach combining water management and Rice-duck farming. Agroecol Sustain Food Syst.

[35] Boni MF, Galvani AP, Wickelgren AL, Malani A (2013). Economic epidemiology of avian influenza on smallholder poultry farms. Theor Pop Biol.

[36] Otte J, Pfeiffer DU, Soares Magalhaes R, Burgos S, Roland-Holst D (2008). Flock size and HPAI risk in Cambodia, Thailand, and Viet Nam.

[37] Delabouglise A, Choisy M, Phan TD, Antoine-Moussiaux N, Peyre M, Vu TD, et al. Economic factors influencing zoonotic disease dynamics: demand for poultry meat and seasonal transmission of avian influenza in Vietnam. Sci Rep. 2017;7(5905). 10.1038/s41598-017-06244-6. 10.1038/s41598-017-06244-6 PMC5517570 28724978

[38] Nguyen LV, Stevenson M, Schauer B, Nguyen DT, Tran QD, Tien TN (2014). Descriptive results of a prospective cohort study of avian influenza in the Mekong River Delta of Viet Nam. Transbound Emerg Dis.

[39] Duc NV, Long T (2008). Poultry production systems in Viet Nam.

[40] Aust P (2015). An assessment of the commercial production of CITES-listed snake species in Viet Nam and China. 28th Meeting of the Animals Committee of the Convention on International Trade of Endangered Species.

