# Supplementary material for: Poultry population dynamics and mortality risks in smallholder farms of the Mekong river delta region
Source: BMC Vet Res. 2019 Jun 17;15:205. doi: 10.1186/s12917-019-1949-y (PMC6580564; doi:10.1186/s12917-019-1949-y)
Supplement: Supplementary file 1 — Questionnaires in English and Vietnamese version. (PDF 747 kb) [file 12917_2019_1949_MOESM1_ESM.pdf]

Additional file 1

Questionnaires in English and Vietnamese version

Farm Code: district ☐☐ – commune ☐☐☐ – farm ☐☐☐

Date of report (d/m/y): _____ / _____ / _____

The form must be filled during the last 5 days of the month

Farmer's Name: ____________________________________

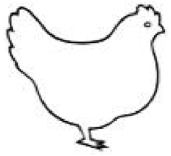

*** Bird Type:** (MC) Meat Chicken (LC) Layer Chicken (BC) Breeding Chicken
(MM) Meat Muscovy Duck (LM) Layer Muscovy Duck (BM) Breeding Muscovy Duck

INFORMATION FROM DATE (D/M/Y): _____ / _____ / _____ TO DATE (D/M/Y): _____ / _____ / _____

		Examples	*Examples*					
Flock Number		*1*	*2*	1	2	3	4	5
Bird Type*		*MC*	*LC, BC*					
Number of Birds		*30*	*10*					
Age (in weeks or months)		*1 – 5 weeks*	*6 months*					
Expected Age of Depop.		*6 – 7 weeks*	*Don't know*					
Managed as all-in all-out?		*No*	*No*					
How many did you buy/hatch this month?		*3*	*0*					
How many did you sell or slaughter?		*10*	*0*					
Commercial Feed?		*No*	*Yes*					
Confine-ment Type	Unconfined	✓						
	House							
	Pen		✓					
Biosecurity	Did you change your boots when managing with your birds?	*N/A*	*No*					
	Did other people (e.g. neighbours, feed delivery) enter the poultry house?	*No*	*Yes, neighbours*					
	Have you used any disinfectants in the past month	*No*	*Yes*					
How many birds died this month?		*7*	*1*					
Dying Symptoms	CNS (e.g. difficult walking, circling)		*circling*					
	Respiratory							
	Diarrhoea	✓						
	Other (e.g. low production)	*anaemia*						
Date of Vaccination	HPAI		*October*					
	Newcastle	*30/11*						
	Other (write name of disease)							

Farm Code: district ☐☐ – commune ☐☐☐ – farm ☐☐☐

Date of report (d/m/y): _____ / _____ / _____

The form must be filled during the last 5 days of the month

Farmer's Name: ______________________________

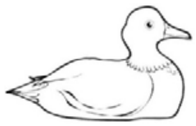

*** Bird Type:** (MD) Meat Duck (LD) Layer Duck (BD) Breeding Duck

INFORMATION FROM DATE (D/M/Y): _____ / _____ / _____ **TO DATE (D/M/Y):** _____ / _____ / _____

		Examples	*Examples*					
Flock Number		*6*	*7*	6	7	8	9	10
Bird Type*		*LD*	*MD*					
Number of Birds		*25*	*35*					
Age (in weeks or months)		*3 months*	*1 – 5 weeks*					
Expected Age of Depop.		*Don't know*	*7 – 8 weeks*					
Managed as all-in all-out?		*No*	*Yes*					
How many did you buy/hatch this month?		*3*	*0*					
How many did you sell or slaughter?		*1*	*0*					
Commercial Feed?		*No*	*Yes*					
Confine -ment	Unconfined		✓					
	Pen	✓						
Grazing	Number of Days	*0*	*30 days*					
	Maximum Distance from home		*200m*					
Biosecurity	Did you change your boots when managing with your birds?	*N/A*	*Yes*					
	Did other people (e.g. neighbours, feed delivery) enter the poultry house?	*N/A*	*Yes, neighbours*					
	Have you used any disinfectants in the past month	*Yes*	*No*					
How many birds have died this month?		*3*	*1*					
Dying Symptoms	CNS (e.g. difficult walking, circling)							
	Respiratory		✓					
	Diarrhoea	✓						
	Other (e.g. anaemia, low production)	*low production*						
Date of Vaccination	HPAI	*October*	*15/11*					
	Duck Plague							
	Other (write name of disease)							

Mã trang trại: huyện [ ][ ] – xã [ ][ ][ ] – trang trại [ ][ ][ ]

Ngày _____ tháng _____ năm _____

Vui lòng thực hiện bản khảo sát này vào 1 trong 5 ngày cuối tháng

Tên chủ hộ: ______________________________

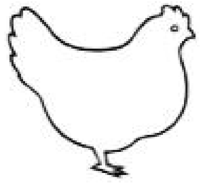

*** Loại gia cầm:** (GT) Gà thịt (GĐ) Gà đẻ (GG) Gà giống (XT) Vịt xiêm thịt (XĐ) Vịt xiêm đẻ (XG) Vịt xiêm giống

**** Ký hiệu viết tắt:** (C) Có (K) Không

THÔNG TIN TỪ NGÀY ______ THÁNG ______ NĂM ______ ĐẾN NGÀY ______ THÁNG ______ NĂM ______

		*Ví dụ***						
Đàn số		*1*	*2*	1	2	3	4	5
Loại gia cầm*		*GT*	*GĐ, GG*					
Số lượng hiện tại		*30*	*10*					
Tuần tuổi / Tháng tuổi		*1 – 5 tuần*	*6 tháng*					
Tuổi xuất chuồng dự kiến		*6 – 7 tuần*						
Quản lý theo hình thức nhập – xuất đồng loạt		*K*	*K*					
Số lượng mua / ấp nở thêm trong tháng rồi		*3*	*0*					
Số lượng bán / giết mổ trong tháng rồi		*10*	*0*					
Dùng thức ăn công nghiệp?		*K*	*C*					
Hình thức nuôi	Thả tự do	✓						
	Nuôi trong nhà		✓					
	Rào (ngoài trời)							
An toàn sinh học	Người nuôi có thay ủng khi vào khu vực nuôi nhốt không?		*K*					
	Ngoài những người trong trang trại, có ai khác vào khu vực nuôi không?	*K*	*C (hàng xóm)*					
	Trong tháng rồi, khu vực nuôi có được khử trùng không?	*K*	*C*					
Số lượng chết trong tháng rồi		*7*	*1*					
Triệu chứng (TC) trước khi chết	TC thần kinh (VD: đi lảo đảo, quay vòng tròn…)		*đi lảo đảo*					
	TC hô hấp (VD: thở khò khè, chảy nước mũi…)							
	Tiêu chảy	✓						
	TC khác (VD: ủ rũ, da tái, đẻ ít, chậm lớn…)	*ủ rũ, xù lông*						
Ngày tiêm chủng	Cúm gia cầm		*tháng 10*					
	Newcastle	*30/11*						
	Bệnh khác (ghi rõ tên)							

Mã trang trại: *huyện* [ ][ ] – *xã* [ ][ ][ ] – *trang trại* [ ][ ][ ]

Ngày _____ tháng _____ năm _____

Vui lòng thực hiện bản khảo sát này vào 1 trong 5 ngày cuối tháng

Tên chủ hộ: ______________________________

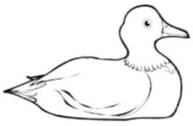

*** Loại gia cầm:** (VT) Vịt thịt (VĐ) Vịt đẻ (VG) Vịt giống

**** Ký hiệu viết tắt:** (C) Có (K) Không

THÔNG TIN TỪ NGÀY ______ THÁNG ______ NĂM ______ ĐẾN NGÀY ______ THÁNG ______ NĂM ______

		*Ví dụ***						
Đàn số		*6*	*7*	6	7	8	9	10
Loại gia cầm*		*VĐ*	*VT*					
Số lượng hiện tại		*25*	*35*					
Tuần tuổi / Tháng tuổi		*3 tháng*	*1 – 5 tuần*					
Tuổi xuất chuồng dự kiến			*7 – 8 tuần*					
Quản lý theo hình thức nhập – xuất đồng loạt		*K*	*C*					
Số lượng mua / ấp nở thêm trong tháng rồi		*3*	*0*					
Số lượng bán / giết mổ trong tháng rồi		*1*	*0*					
Dùng thức ăn công nghiệp?		*K*	*C*					
Hình thức nuôi	Thả tự do		✓					
	Rào (ngoài trời)	✓						
Chăn thả	Số ngày chăn thả	*0*	*30 ngày*					
	Khoảng cách (xa nhất) từ bãi chăn thả đến nơi ở		*200m*					
An toàn sinh học	Người nuôi có thay ủng khi vào khu vực nuôi nhốt không?		*C*					
	Ngoài những người trong trang trại, có ai khác vào khu vực nuôi không?		*C (hàng xóm)*					
	Trong tháng rồi, khu vực nuôi có được khử trùng không?	*C*	*K*					
Số lượng chết trong tháng rồi		*3*	*1*					
Triệu chứng (TC) trước khi chết	TC thần kinh (VD: đi lảo đảo, quay vòng tròn…)							
	TC hô hấp (VD: thở khò khè, chảy nước mũi…)		✓					
	Tiêu chảy	✓						
	TC khác (VD: ủ rũ, da tái, đẻ ít, chậm lớn…)	*đẻ ít*						
Ngày tiêm chủng	Cúm gia cầm	*tháng 10*	*15/11*					
	Dịch tả vịt							
	Bệnh khác (ghi rõ tên)							
