# Supplementary material for: Poultry population dynamics and mortality risks in smallholder farms of the Mekong river delta region
Source: BMC Vet Res. 2019 Jun 17;15:205. doi: 10.1186/s12917-019-1949-y (PMC6580564; doi:10.1186/s12917-019-1949-y)
Supplement: Supplementary file 2 — Description of the flock-matching algorithm. (PDF 1246 kb) [file 12917_2019_1949_MOESM2_ESM.pdf]

Additional file 2

Description of the flock-matching algorithm

1 Flock matching

The implementation is available on a public repository.

Consider the data we have for a farm at month i. It has got n_i flocks, numeroted $1 \leq j \leq n_i$. We write F_j for the state of flock j, and $\mathcal{F}$ the set of flock states.

From that full state, we will extract the more relevant data for flock matching. A (simplified) flock will then be described by

- Its species (chicken, duck, etc.), written $species(F)$
- Its age, written $age(F)$
- Its population size, written $size(F)$
- Its use (meat, layer/breeding, young), written $use(F)$
- whether it is dead (its population has reached 0) or not, written $dead(F)$

To compare flocks at one-month intervals, we need to predict the state the flock was in one month before. Let p_{rev} be the following function which aims to do that.

$$p_{rev}(F) = \begin{pmatrix} species(F), \\ use(F), \\ size(F) - n_{in}(F) + n_{sold}(F) + n_{dead}(F), \\ age(F) - 1month, \\ 0 \end{pmatrix}$$

Let us now define a (pseudo-)distance between two flocks as

$$d(F, F') = \sum_{i \in \{species, use, size, age, dead\}} \alpha_i * d_i(F_i, F_i^{'})$$

where we take d_i to be the discrete distance for the discrete variables $species$, use, and $dead$, and the Manhattan (taxicab) distance for the continuous variables $size$ and age, with

- $\alpha_{species} = 100$
- $\alpha_{use} = 10$
- $\alpha_{size} = 1$
- $\alpha_{age} = 2$
- $\alpha_{dead} = 200$

Because of the presence of missing data (NA), each distance component d is modified in the following way :

$$d \diamond (x, y) = \begin{cases} d(x, y) & \text{if } x, y \neq \text{NA} \\ 0 & \text{if } x = \text{NA} = y \\ \alpha_{NA} & \text{if } x = \text{NA and } y \neq \text{NA or } y = \text{NA and } x \neq \text{NA} \end{cases}$$

Those functions are distances only if NA is more penalized than any other value. This is not what we want, and we choose $\alpha_{NA} = 10$. The algorithm doesn't rely on d being an actual distance however, so this is not a problem.

The coefficients were chosen to reflect the relative magnitude of errors for the different input fields, and we confirmed that they seemed to select the right solution.

We also define two additional special states, x^{new} and $x^{removed}$, corresponding to a flock present at month i disappearing at month $i+1$ – we will say that the flock is *removed* at month $i+1$; and to a flock appearing at month $i+1$ – we will say that the flock is *new* at month i.

$$x^{new} = \begin{pmatrix} \text{NA} \\ \text{NA} \\ 0 \\ 0 \\ 0 \end{pmatrix}$$

$$x^{removed} = \begin{pmatrix} \text{NA} \\ \text{NA} \\ 0 \\ \text{NA} \\ 1 \end{pmatrix}$$

Those states are special in that any number of flocks can appear or disappear in a month.

We can now rephrase the problem somewhat. For two flocks a from month $i-1$ and b from month i, we define the cost C of pairing the flocks

$$C(a, b) = d \diamond (p_{rev}(a), b)$$

We can also define the cost of a being new at month i by

$$N(a) = d \diamond (p_{rev}(a), x^{new})$$

And the cost of b being removed at month i by

$$D(b) = d \diamond (b, x^{removed})$$

With flocks $a \in A$ at month i and flocks $b \in B$ at month $i-1$, the problem

can be phrased as

$$\text{minimize} \sum_{a\in A}\sum_{b\in B} C(a,b)x_{a,b} + \sum_{a\in A} N(a)x_a^N + \sum_{b\in B} D(b)x_b^D \tag{1}$$

$$\text{subject to} \sum_{b\in B} x_{a,b} + x_a^N = 1, \forall a \in A \tag{2}$$

$$\text{and} \sum_{a\in A} x_{a,b} + x_b^D = 1, \forall b \in B \tag{3}$$

As we noted earlier, it is actually quite easy for a human to match flocks by hand. This is due to the fact that simply assigning to each flock the flock that most resembles it the next month often works. In that case we say that there are no conflicts. This works if no two flocks share the same best-matching flock, (or that best matching flock is the *new* or *removed* flock.

Therefore, our algorithm first checks for conflicts, and if there are conflicts, then proceeds to do an exhaustive search. This takes reasonable time, as the number of flocks to match in one go is always reasonable (around 10). For bigger problems, one would have to use a more efficient algorithm.

Here is a description of the algorithm in pseudo-code.

1. *FOR* each farm
   (a) *FOR* each month $i \geq 1$
      i. compute x_i, the list $x_{i,j} = p(F_{i,j})$, with the special state *new* at the end. and $x_i^{'}$, the list $x_{i,j}^{'} = p_{rev}(F_{i+1,j})$, with the special state *removed* at the end.
      ii. compute A_i the $n \times m$ matrix of distances $a_{j,j'}$ between x_i and $x_i^{'}$.
      iii. for each row/column of A but the last, find the column/row with the minimum cost.
      iv. check for *conflicts*, which happen if the min column/row is the same for more than one row/column, or if the minimum column $j_2^{'}$ for the minimum row j for a column $j_1^{'}$ isn't the starting column, $j_1^{'} \neq j_2^{'}$. Those conditions don't need to be verified for the last row and column : they can be the minimum for several row/column.
      v. If there are no *conflicts*, then the best matching is constituted by those minimas.
      vi. If there are *conflicts*, we proceed to an exhaustive search among all valid assignments to find the best one. This is doable as the size of the graph is always small (around 10 flocks on each side).
      vii. New flocks on the right (those matching *new*) are assigned a new flock identifier, other flocks are assigned the same identifier as the flock they were matched to.

A description of the Python implementation (as well as the implementation itself), is directly in the code.

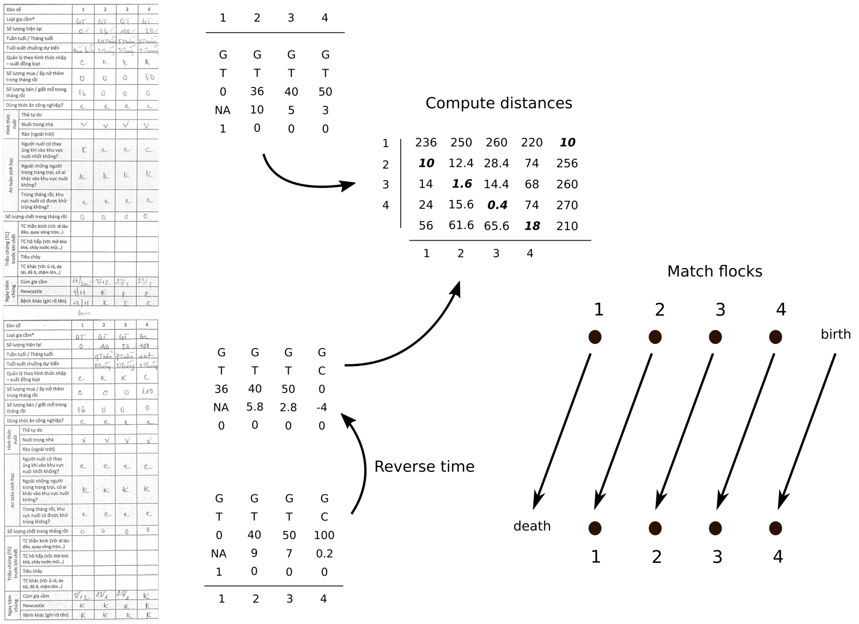

Figure 1: Example of flock matching on data from our dataset, when an exhaustive search is not necessary
