# Supplementary material for: Poultry population dynamics and mortality risks in smallholder farms of the Mekong river delta region
Source: BMC Vet Res. 2019 Jun 17;15:205. doi: 10.1186/s12917-019-1949-y (PMC6580564; doi:10.1186/s12917-019-1949-y)
Supplement: Supplementary file 3 — Timeline of poultry flocks in each study farm. (PDF 559 kb) [file 12917_2019_1949_MOESM3_ESM.pdf]

Additional file 3

Timeline of poultry flocks in each study farm

Farms are numbered from 1 to 26 (Tan Loc commune) or from 1 to 27 (Tan Phu commune).

Flocks are represented by straight lines starting on the month the first birds are introduced and finishing on the month the last birds are sold. Specific events (introduction, removal and deaths attributable to diseases) are displayed on each flock on the month they occurred. Vertical dashed lines indicate months during which more than 10% of the poultry initially present in the farm died due to disease.

Code of poultry type and specie

LD: layer duck

BD: broiler duck

YD: duckling

PH: pheasant

Q: quail

P: pigeon

LMD: layer Muscovy duck

NMD: broiler Muscovy duck

YMD: young Muscovy duck

LG: layer geese

BG: broiler geese

LC: layer chicken

BC: broiler chicken

YC: chicks

Timeline Tan Loc 1

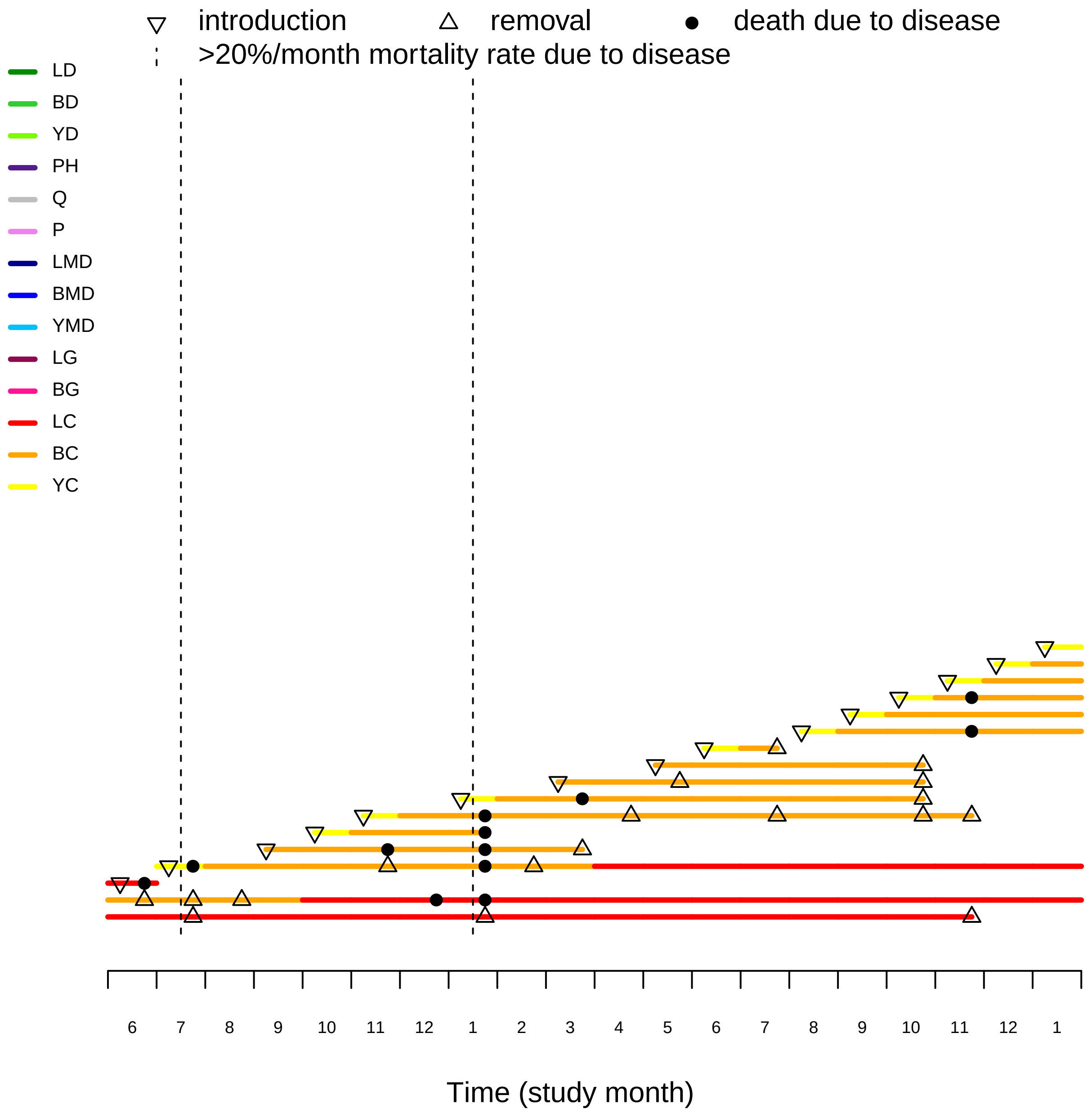

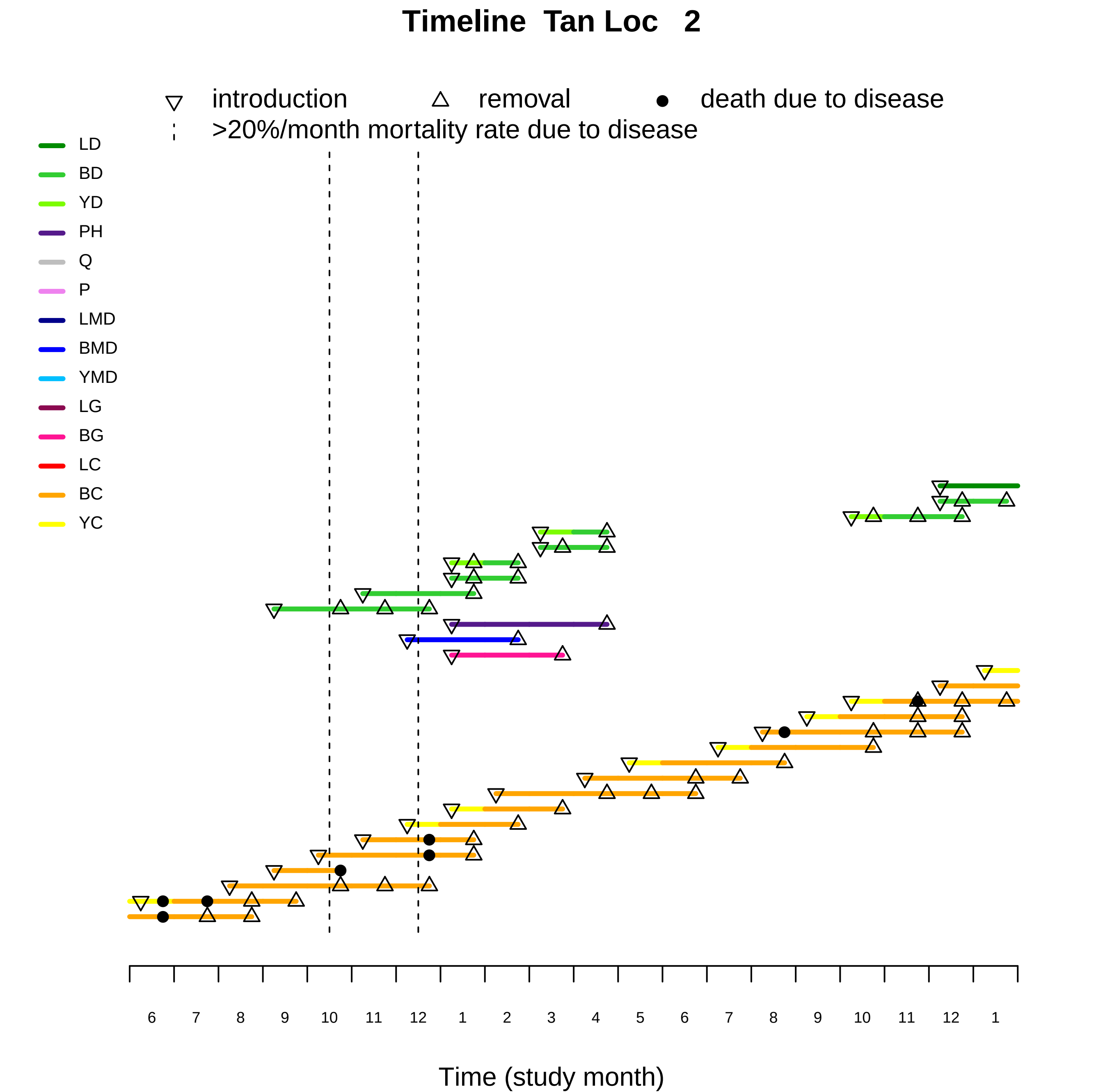
Timeline Tan Loc 2
introduction
removal
death due to disease
>20%/month mortality rate due to disease
LD
BD
YD
PH
Q
P
LMD
BMD
YMD
LG
BG
LC
BC
YC
6 7 8 9 10 11 12 1 2 3 4 5 6 7 8 9 10 11 12 1
Time (study month)

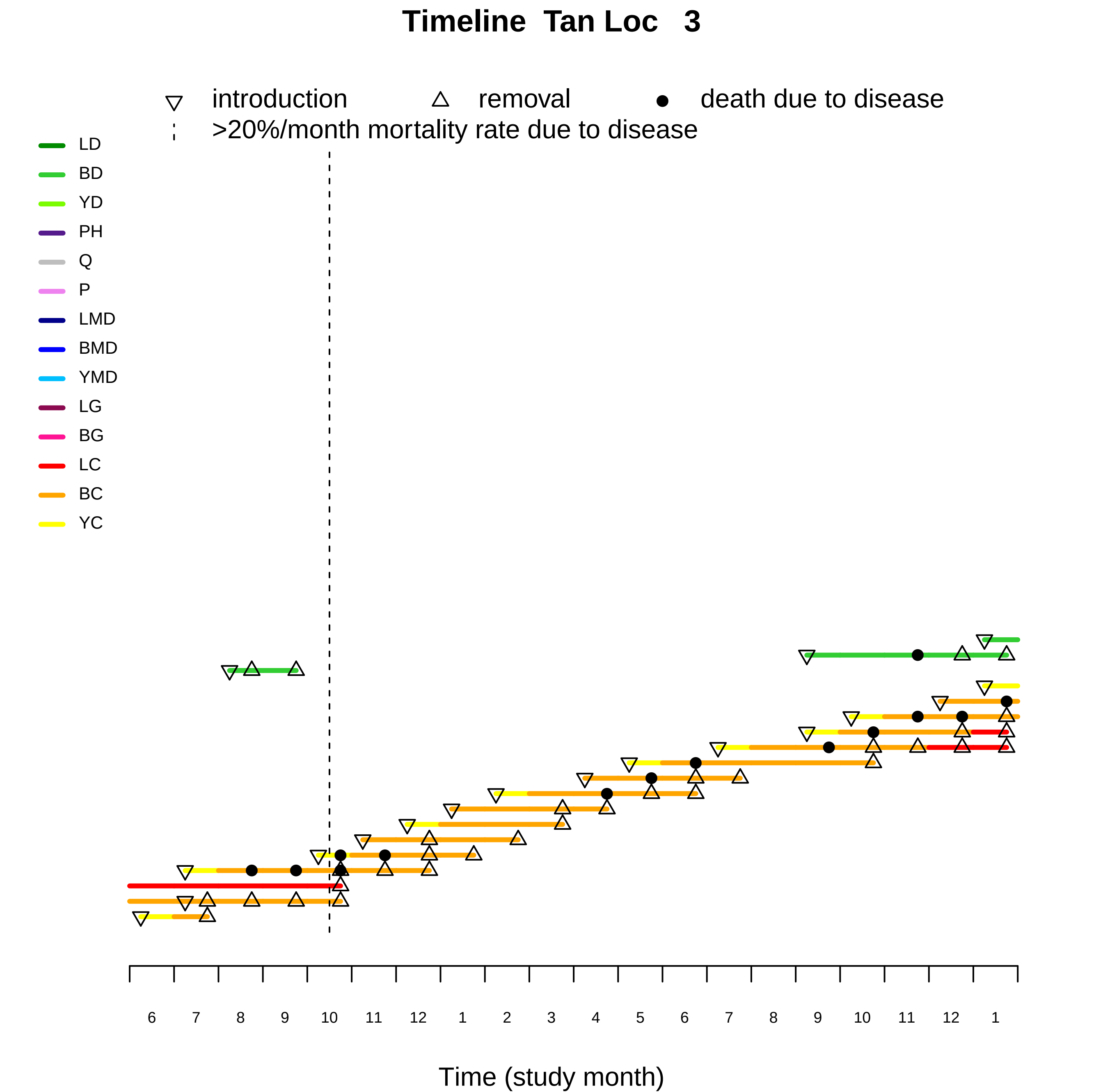
Timeline Tan Loc 3
introduction
removal
death due to disease
>20%/month mortality rate due to disease
LD
BD
YD
PH
Q
P
LMD
BMD
YMD
LG
BG
LC
BC
YC
6 7 8 9 10 11 12 1 2 3 4 5 6 7 8 9 10 11 12 1
Time (study month)

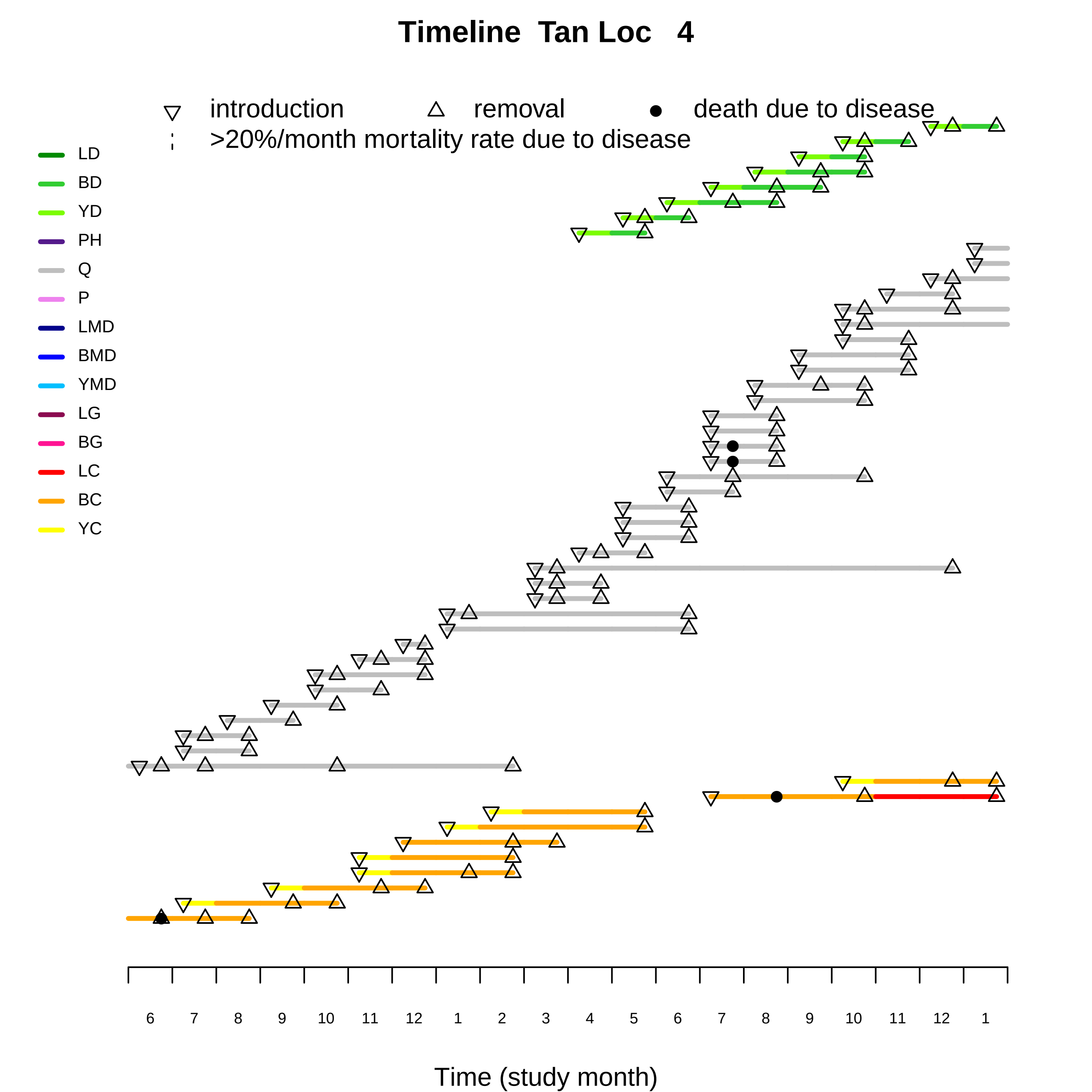

Timeline Tan Loc 4
introduction
removal
death due to disease
>20%/month mortality rate due to disease
LD
BD
YD
PH
Q
P
LMD
BMD
YMD
LG
BG
LC
BC
YC
6 7 8 9 10 11 12 1 2 3 4 5 6 7 8 9 10 11 12 1
Time (study month)

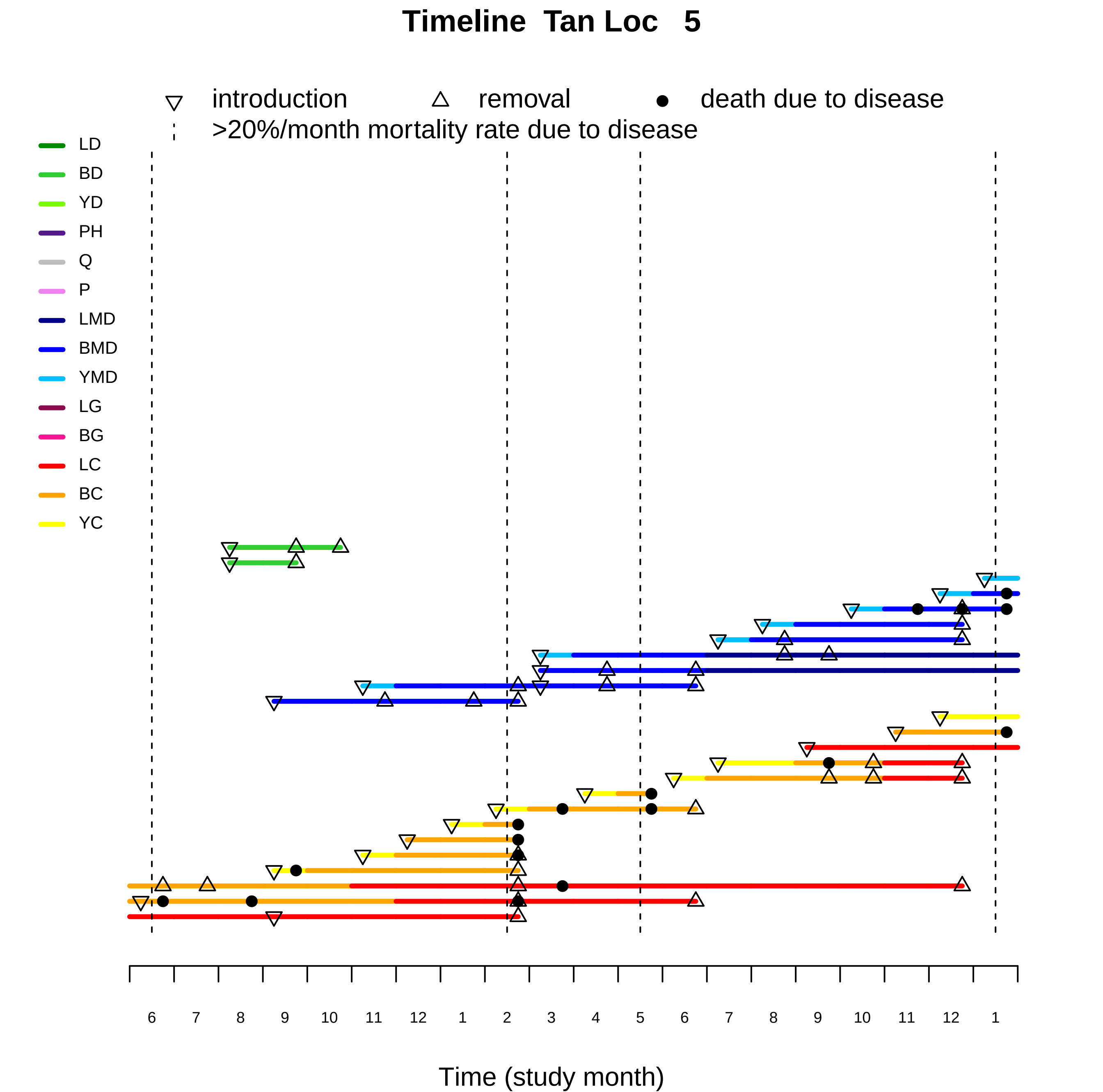

Timeline Tan Loc 5
introduction
removal
death due to disease
>20%/month mortality rate due to disease
LD
BD
YD
PH
Q
P
LMD
BMD
YMD
LG
BG
LC
BC
YC
6
7
8
9
10
11
12
1
2
3
4
5
6
7
8
9
10
11
12
1
Time (study month)

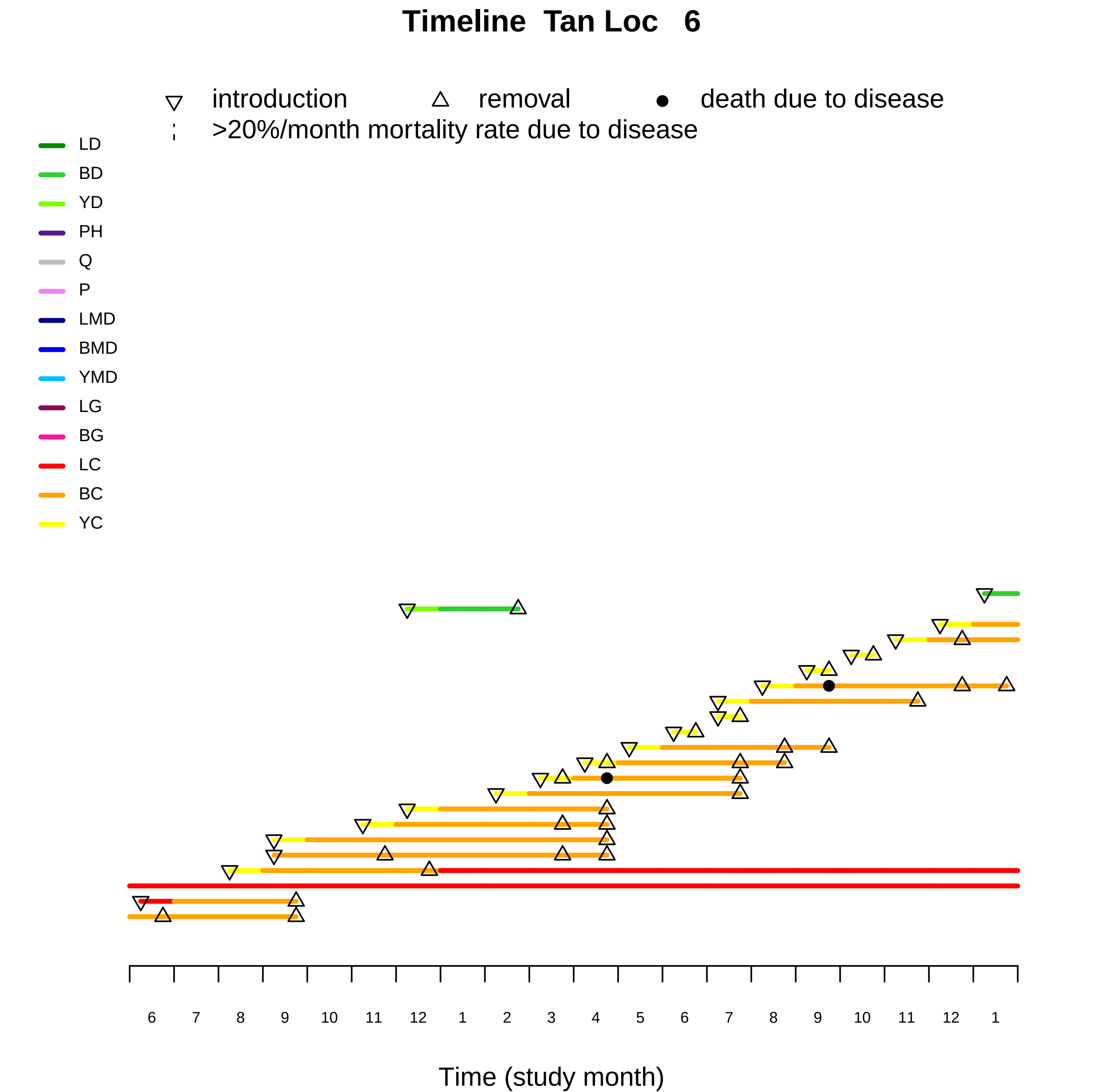
Timeline Tan Loc 6
introduction
removal
death due to disease
>20%/month mortality rate due to disease
LD
BD
YD
PH
Q
P
LMD
BMD
YMD
LG
BG
LC
BC
YC
6 7 8 9 10 11 12 1 2 3 4 5 6 7 8 9 10 11 12 1
Time (study month)

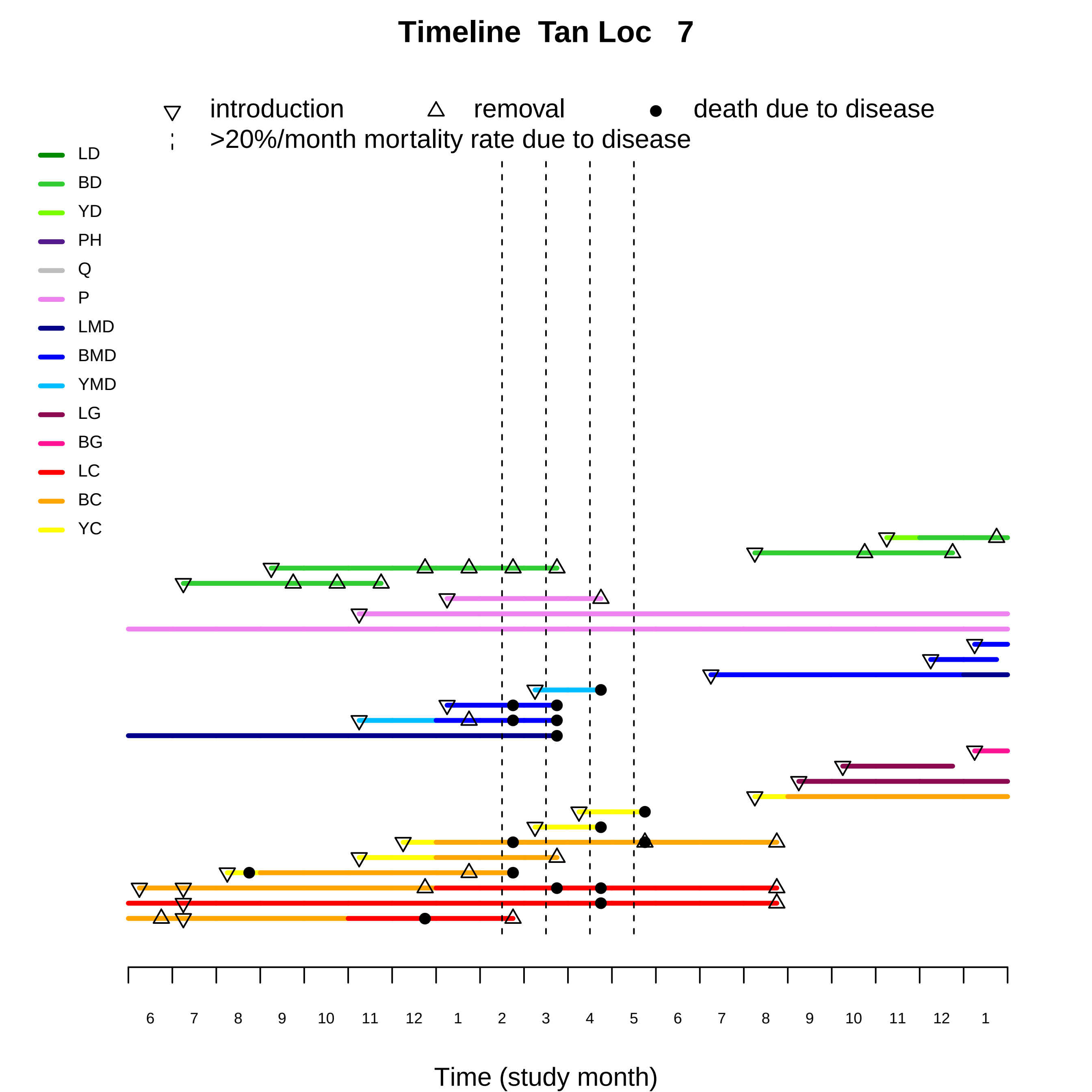

Timeline Tan Loc 7
introduction
removal
death due to disease
>20%/month mortality rate due to disease
LD
BD
YD
PH
Q
P
LMD
BMD
YMD
LG
BG
LC
BC
YC
6
7
8
9
10
11
12
1
2
3
4
5
6
7
8
9
10
11
12
1
Time (study month)

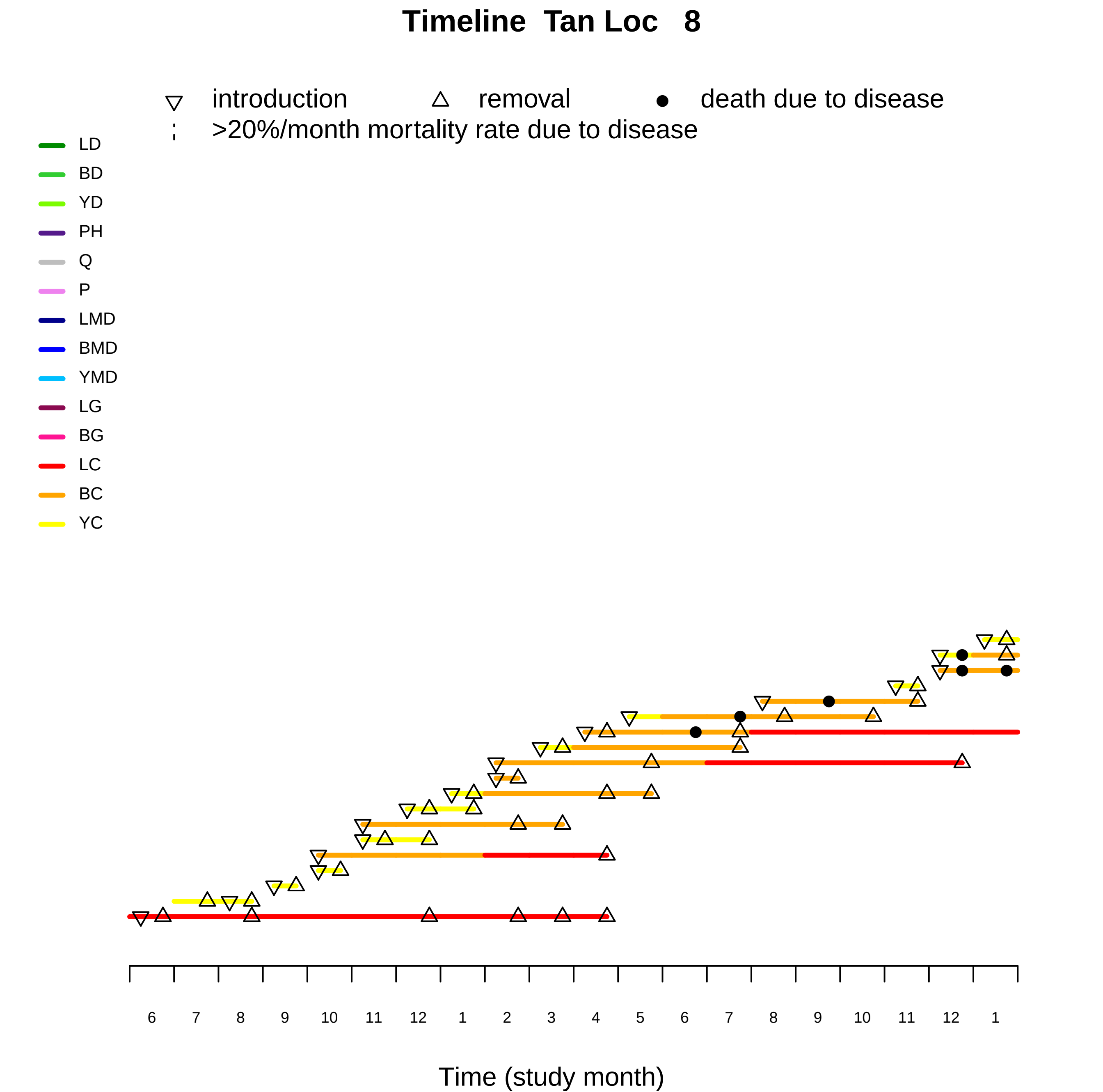
Timeline Tan Loc 8
introduction
removal
death due to disease
>20%/month mortality rate due to disease
LD
BD
YD
PH
Q
P
LMD
BMD
YMD
LG
BG
LC
BC
YC
6 7 8 9 10 11 12 1 2 3 4 5 6 7 8 9 10 11 12 1
Time (study month)

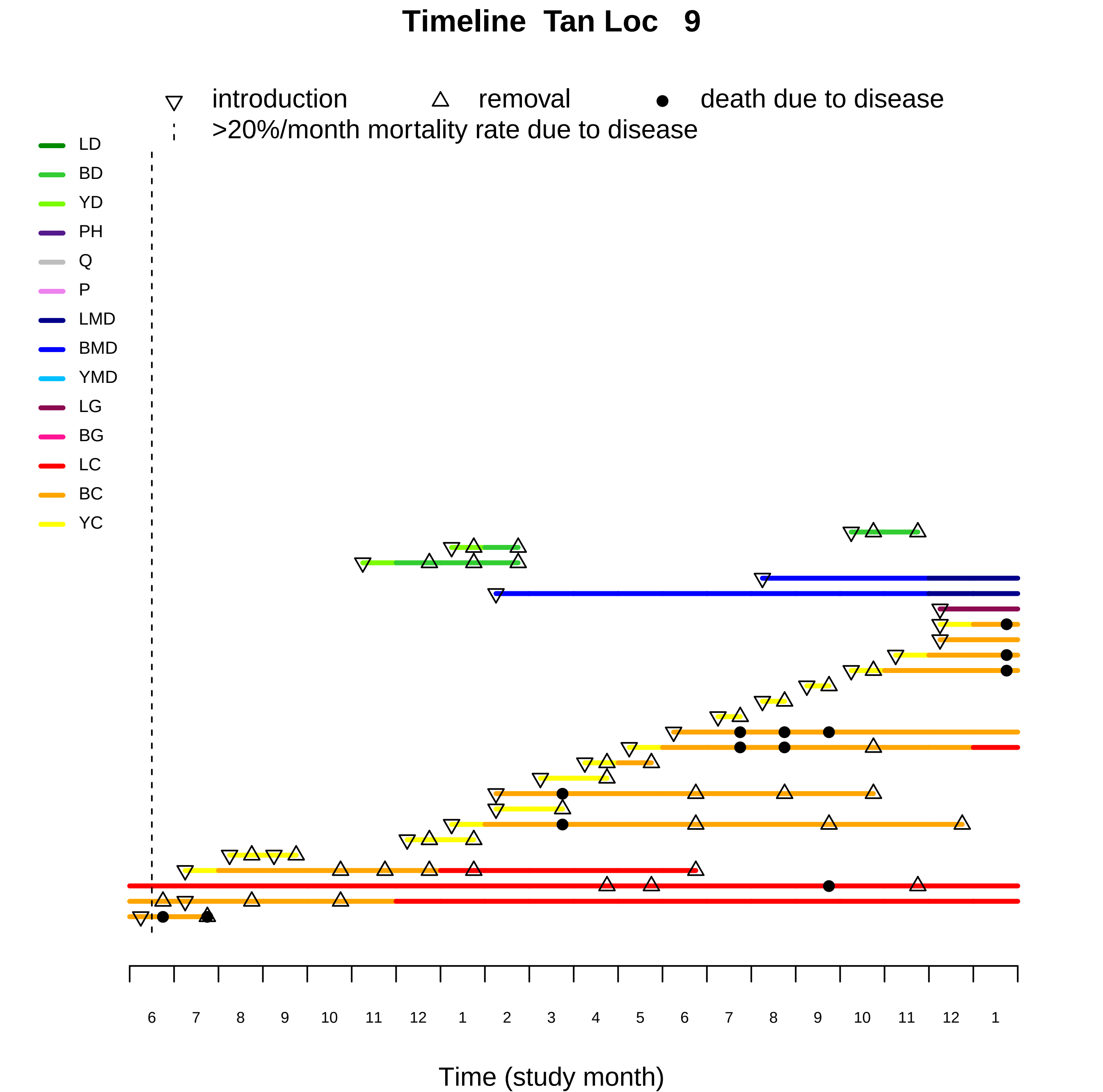
Timeline Tan Loc 9
introduction
removal
death due to disease
>20%/month mortality rate due to disease
LD
BD
YD
PH
Q
P
LMD
BMD
YMD
LG
BG
LC
BC
YC
6
7
8
9
10
11
12
1
2
3
4
5
6
7
8
9
10
11
12
1
Time (study month)

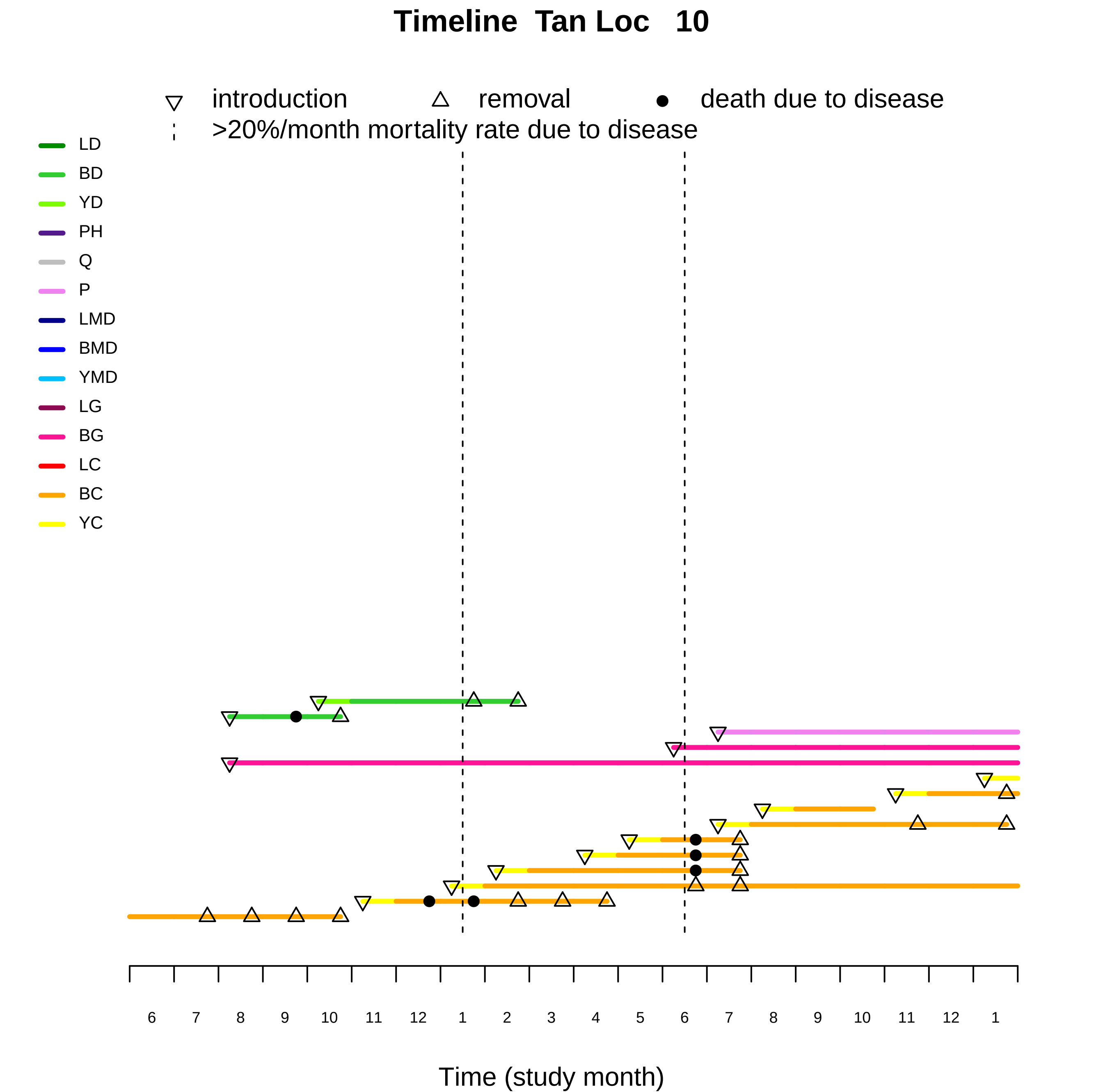
Timeline Tan Loc 10
introduction
removal
death due to disease
>20%/month mortality rate due to disease
LD
BD
YD
PH
Q
P
LMD
BMD
YMD
LG
BG
LC
BC
YC
6
7
8
9
10
11
12
1
2
3
4
5
6
7
8
9
10
11
12
1
Time (study month)

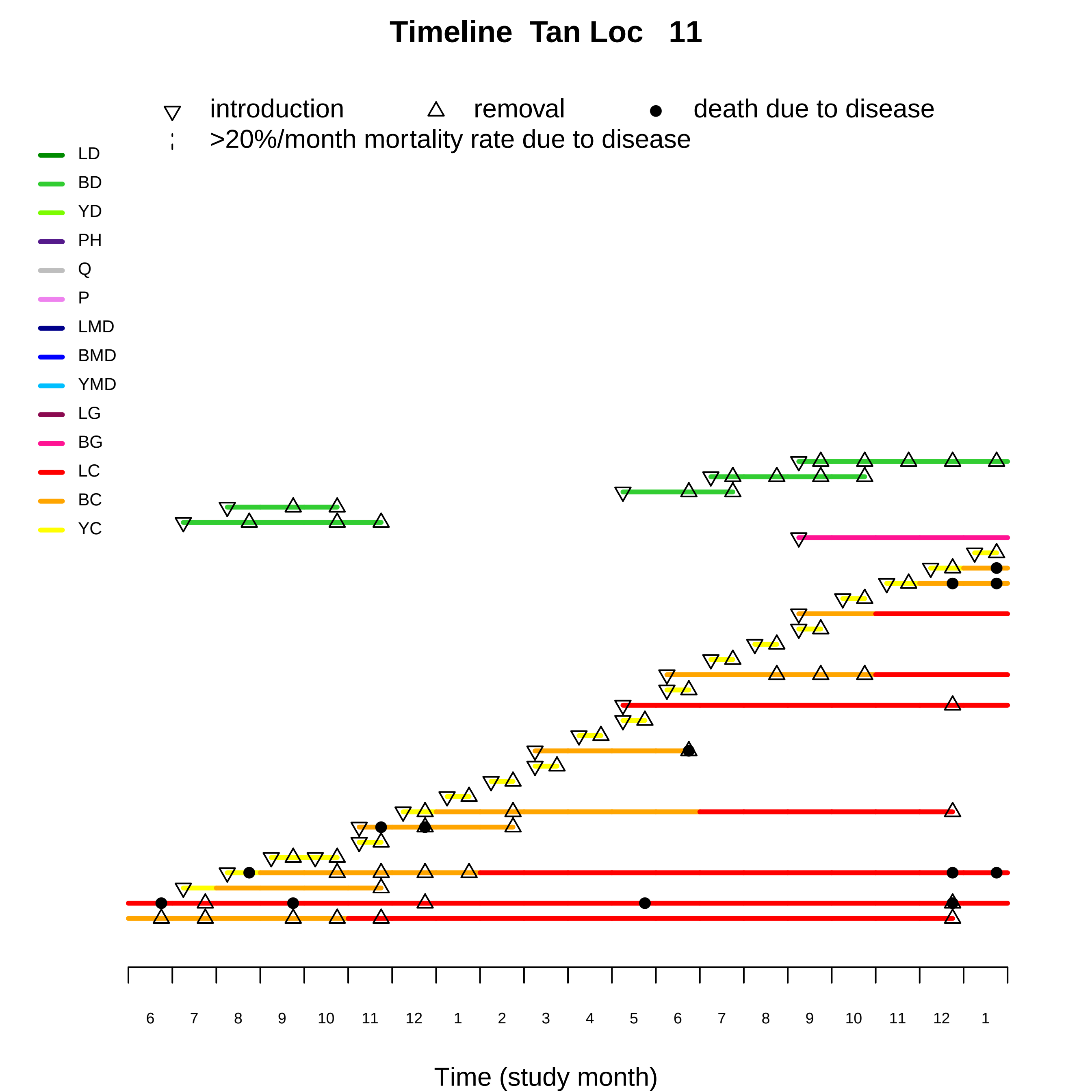

Timeline Tan Loc 11
introduction
removal
death due to disease
>20%/month mortality rate due to disease
LD
BD
YD
PH
Q
P
LMD
BMD
YMD
LG
BG
LC
BC
YC
6
7
8
9
10
11
12
1
2
3
4
5
6
7
8
9
10
11
12
1
Time (study month)

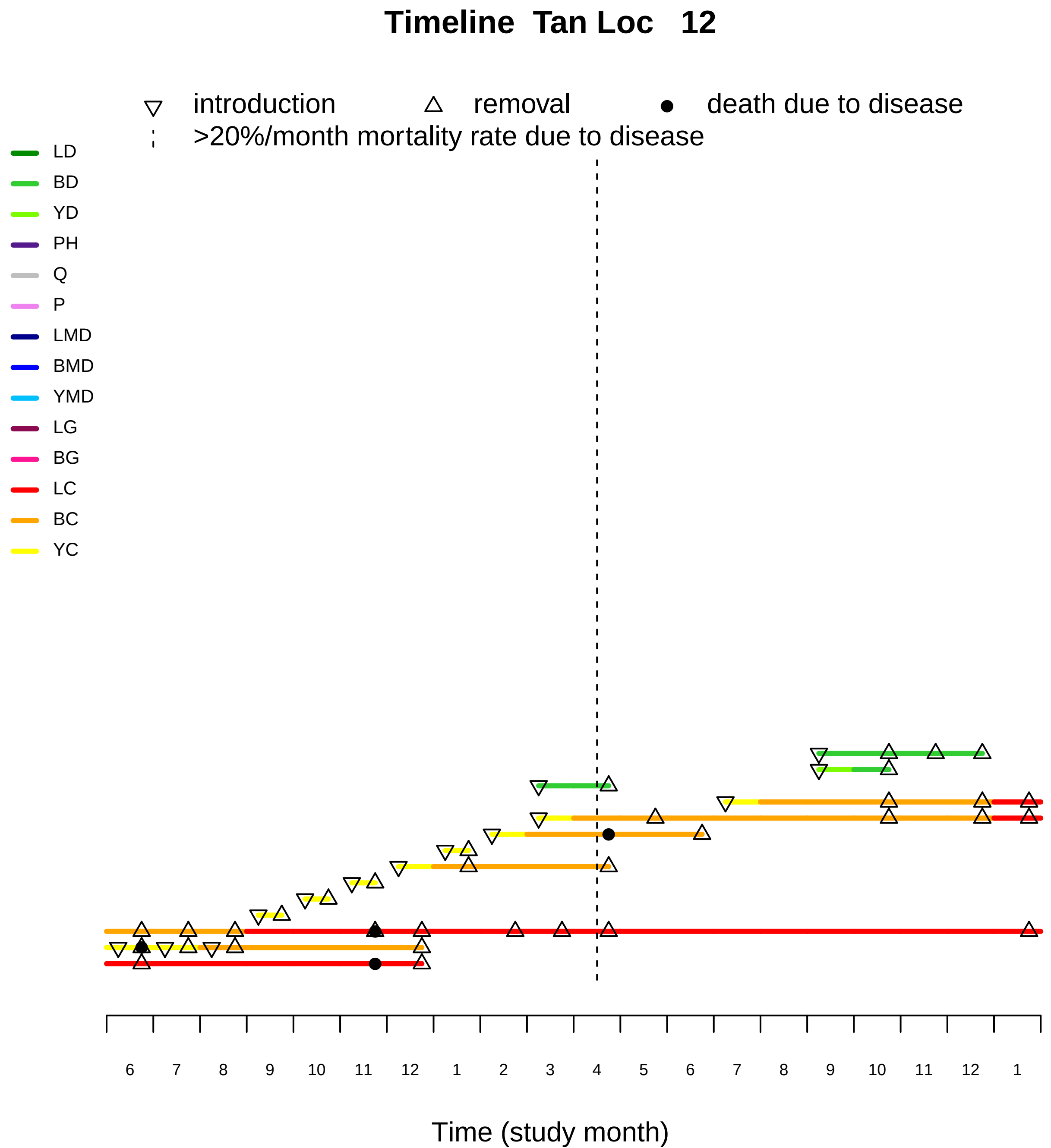
Timeline Tan Loc 12
introduction
removal
death due to disease
>20%/month mortality rate due to disease
LD
BD
YD
PH
Q
P
LMD
BMD
YMD
LG
BG
LC
BC
YC
6
7
8
9
10
11
12
1
2
3
4
5
6
7
8
9
10
11
12
1
Time (study month)

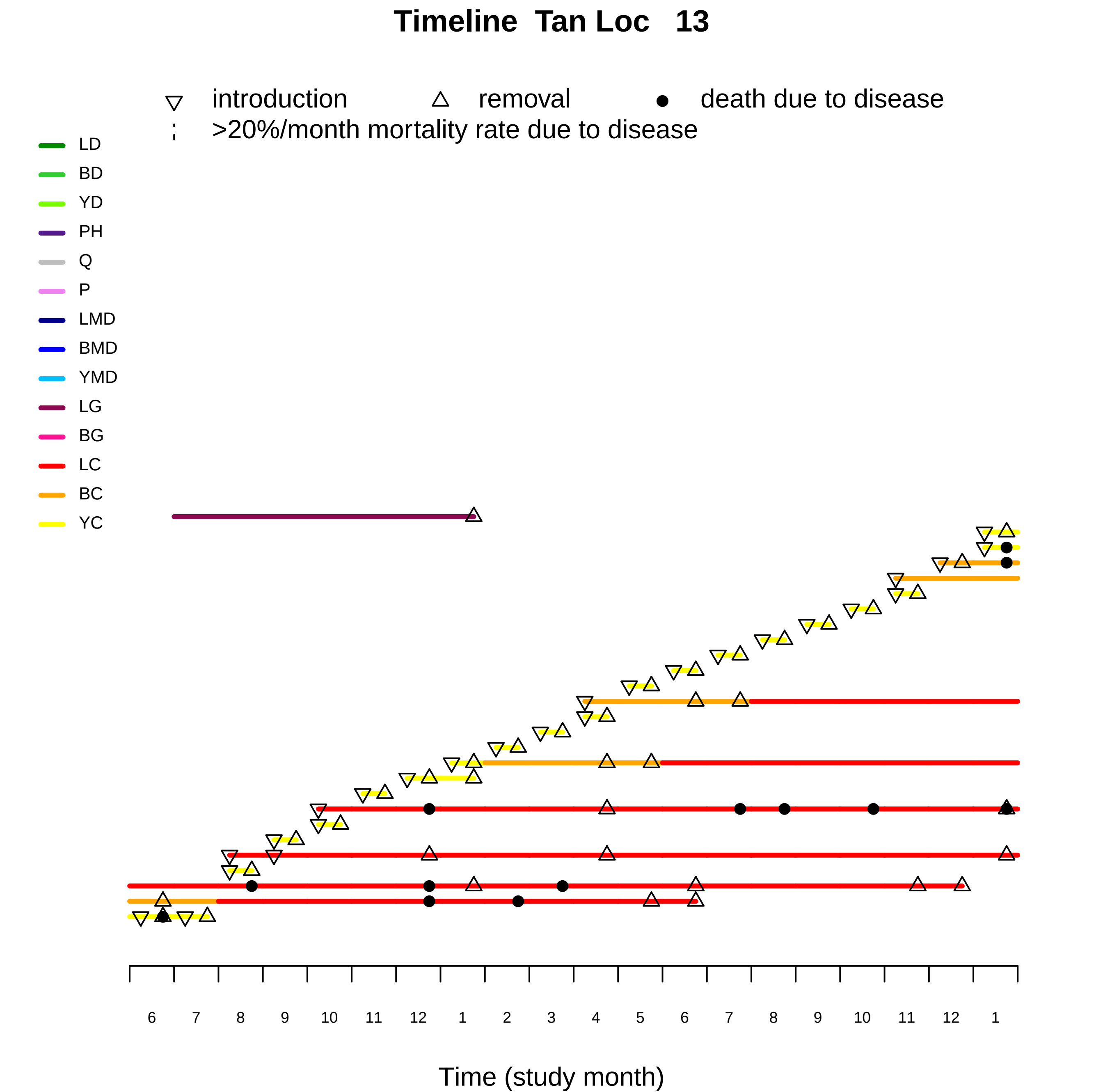
Timeline Tan Loc 13
introduction
removal
death due to disease
>20%/month mortality rate due to disease
LD
BD
YD
PH
Q
P
LMD
BMD
YMD
LG
BG
LC
BC
YC
6 7 8 9 10 11 12 1 2 3 4 5 6 7 8 9 10 11 12 1
Time (study month)

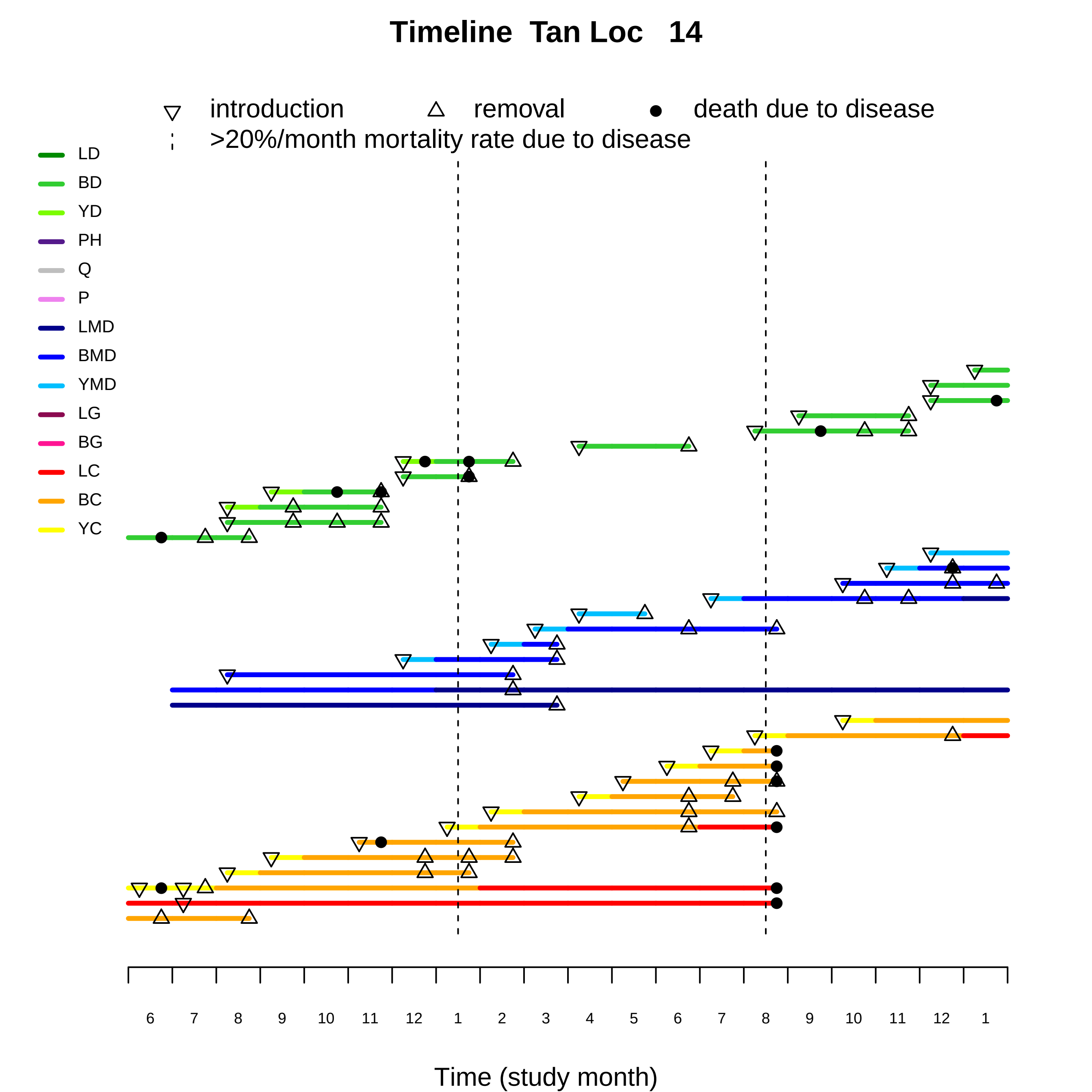

Timeline Tan Loc 14
introduction
removal
death due to disease
>20%/month mortality rate due to disease
LD
BD
YD
PH
Q
P
LMD
BMD
YMD
LG
BG
LC
BC
YC
6
7
8
9
10
11
12
1
2
3
4
5
6
7
8
9
10
11
12
1
Time (study month)

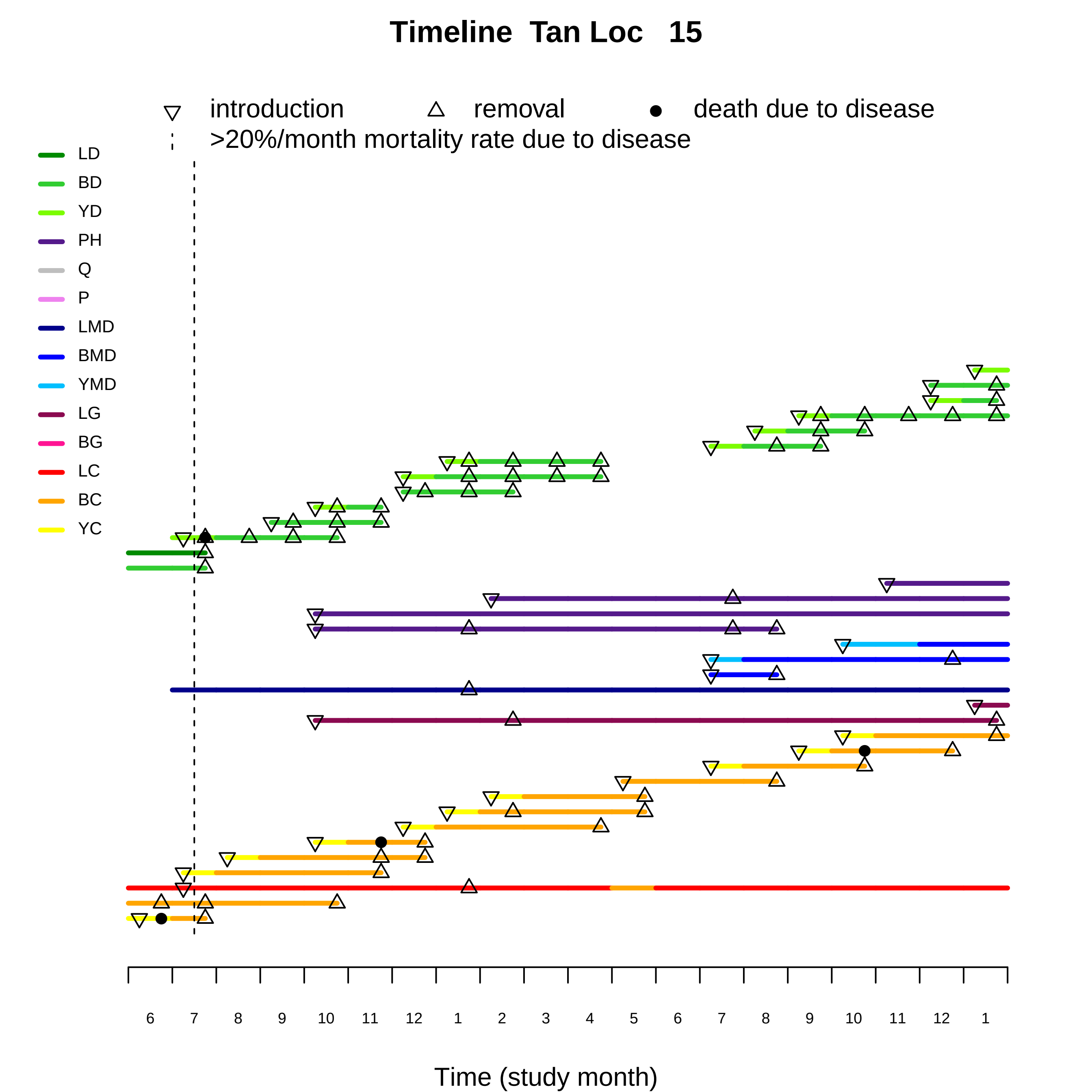
Timeline Tan Loc 15
introduction
removal
death due to disease
>20%/month mortality rate due to disease
LD
BD
YD
PH
Q
P
LMD
BMD
YMD
LG
BG
LC
BC
YC
6 7 8 9 10 11 12 1 2 3 4 5 6 7 8 9 10 11 12 1
Time (study month)

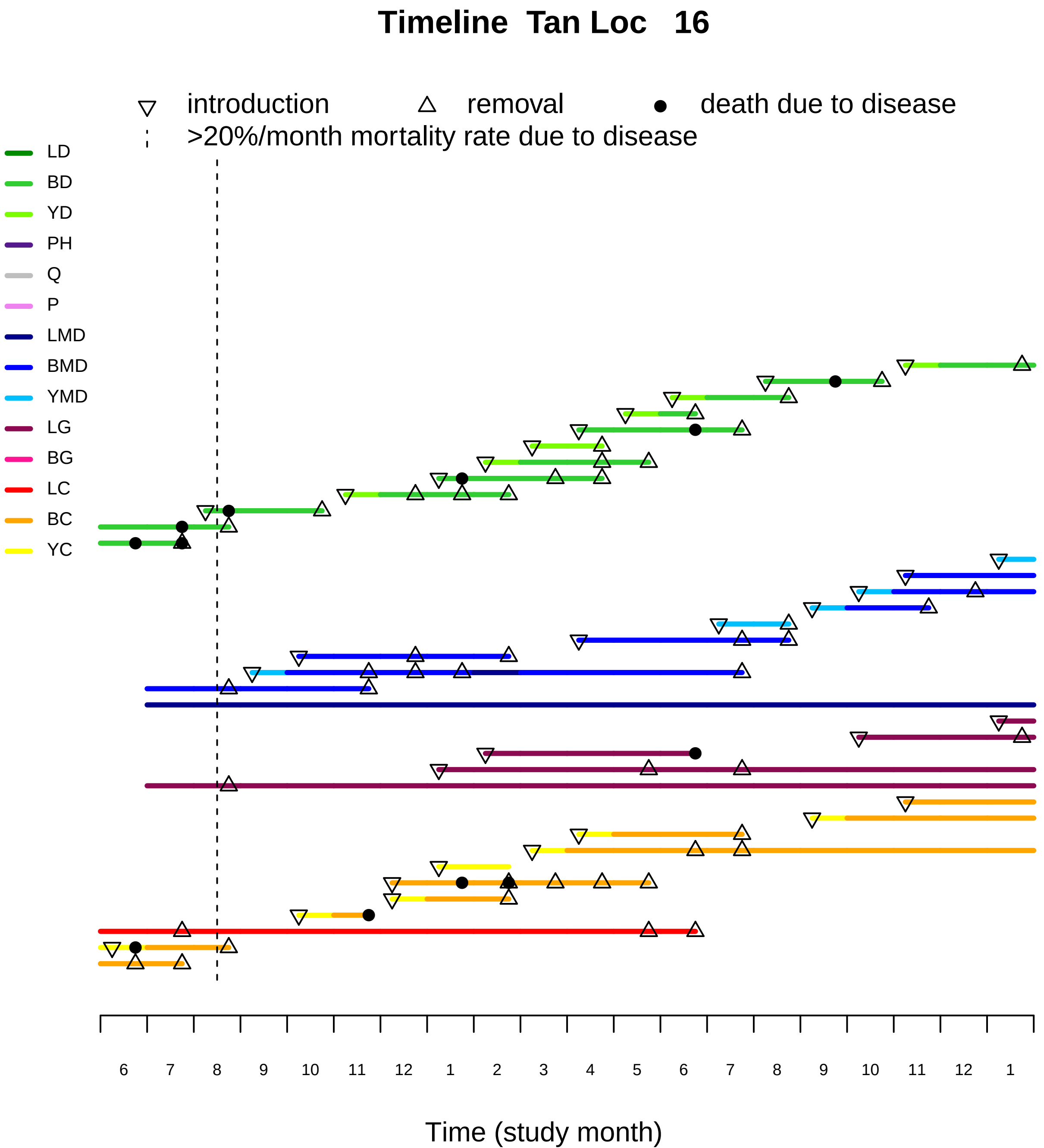

Timeline Tan Loc 16
introduction
removal
death due to disease
>20%/month mortality rate due to disease
LD
BD
YD
PH
Q
P
LMD
BMD
YMD
LG
BG
LC
BC
YC
6
7
8
9
10
11
12
1
2
3
4
5
6
7
8
9
10
11
12
1
Time (study month)

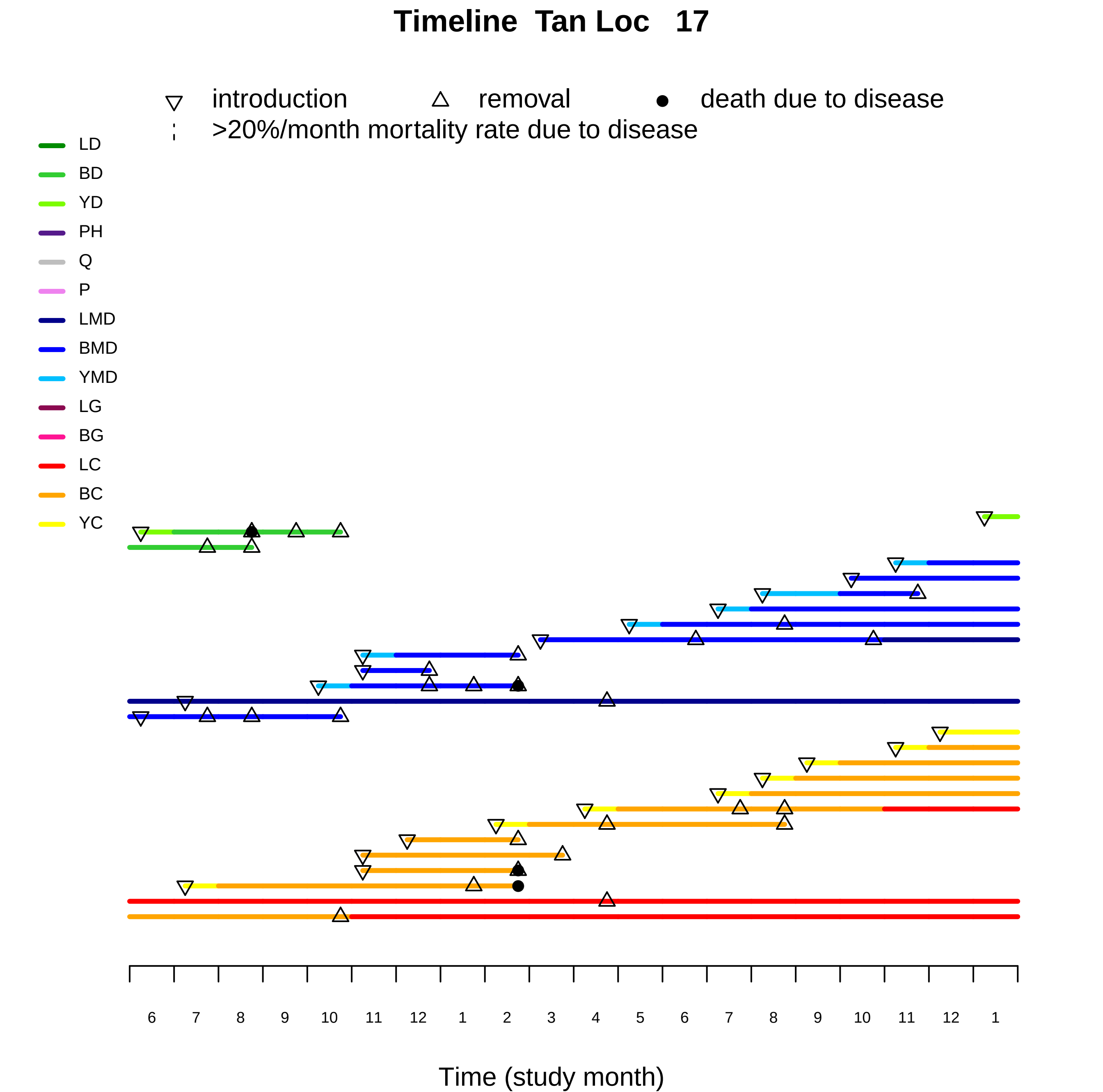

Timeline Tan Loc 17
introduction
removal
death due to disease
>20%/month mortality rate due to disease
LD
BD
YD
PH
Q
P
LMD
BMD
YMD
LG
BG
LC
BC
YC
6
7
8
9
10
11
12
1
2
3
4
5
6
7
8
9
10
11
12
1
Time (study month)

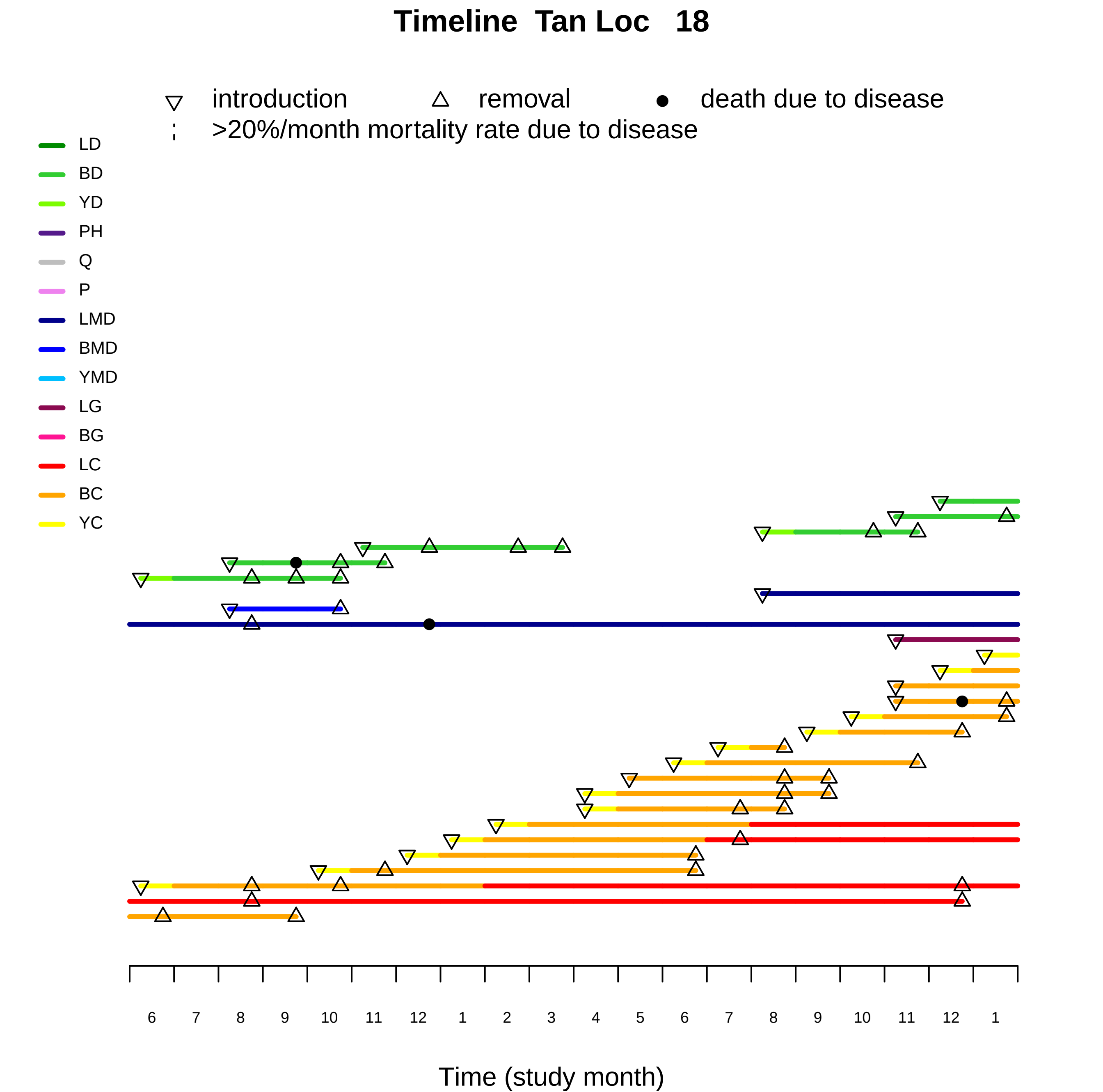

Timeline Tan Loc 18
introduction
removal
death due to disease
>20%/month mortality rate due to disease
LD
BD
YD
PH
Q
P
LMD
BMD
YMD
LG
BG
LC
BC
YC
6
7
8
9
10
11
12
1
2
3
4
5
6
7
8
9
10
11
12
1
Time (study month)

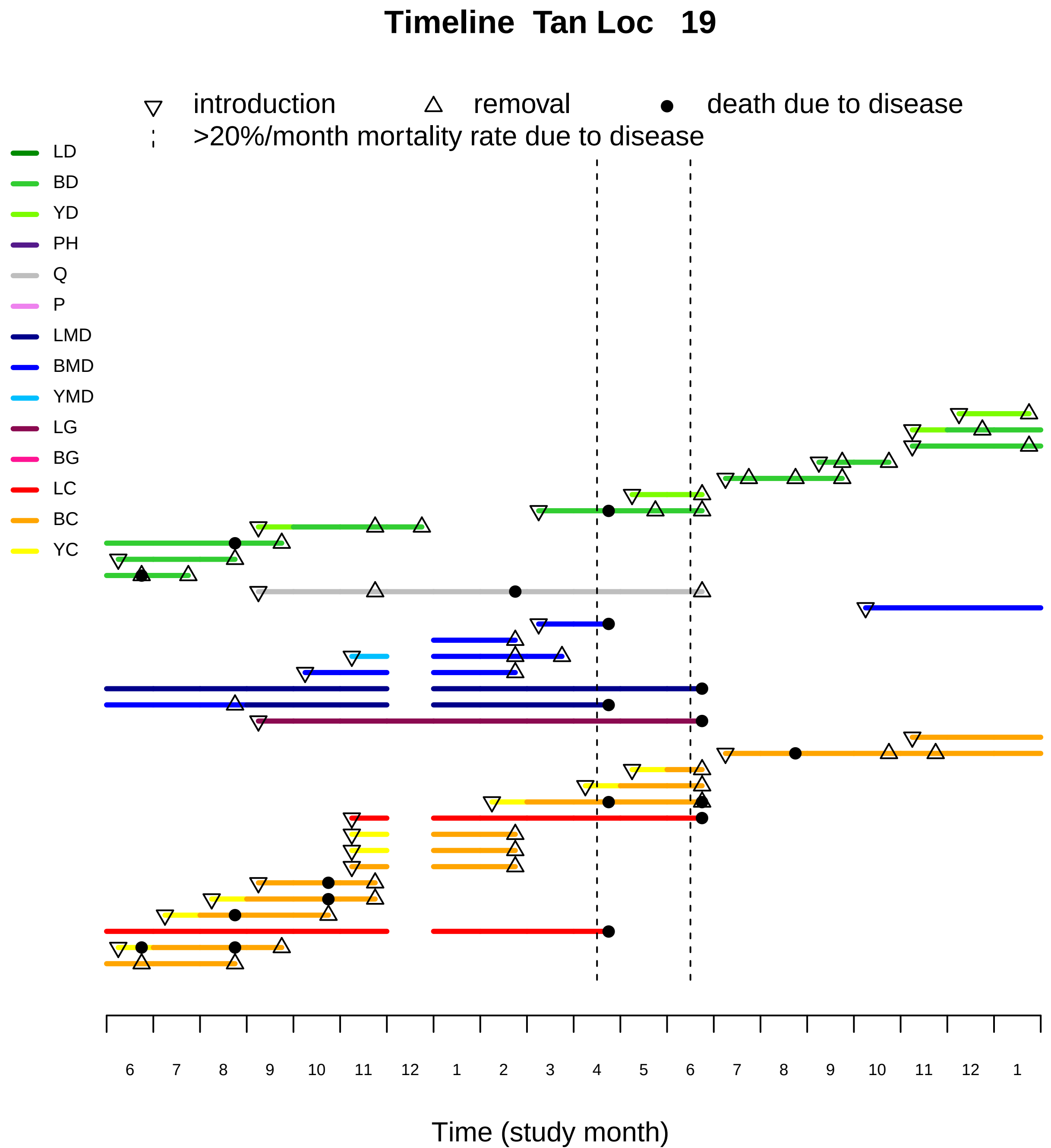
Timeline Tan Loc 19
introduction
removal
death due to disease
>20%/month mortality rate due to disease
LD
BD
YD
PH
Q
P
LMD
BMD
YMD
LG
BG
LC
BC
YC
6 7 8 9 10 11 12 1 2 3 4 5 6 7 8 9 10 11 12 1
Time (study month)

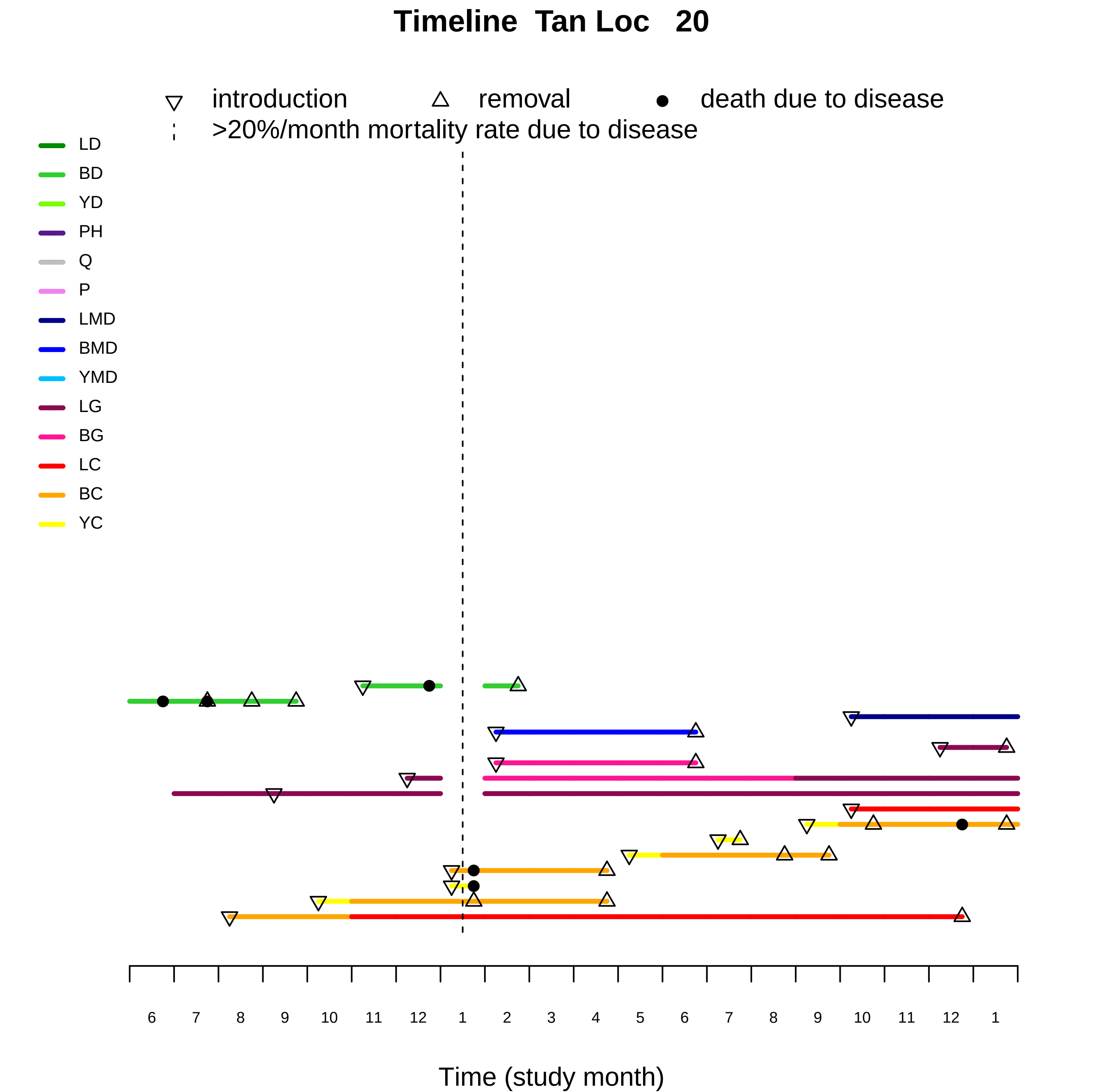
Timeline Tan Loc 20
introduction
removal
death due to disease
>20%/month mortality rate due to disease
LD
BD
YD
PH
Q
P
LMD
BMD
YMD
LG
BG
LC
BC
YC
6
7
8
9
10
11
12
1
2
3
4
5
6
7
8
9
10
11
12
1
Time (study month)

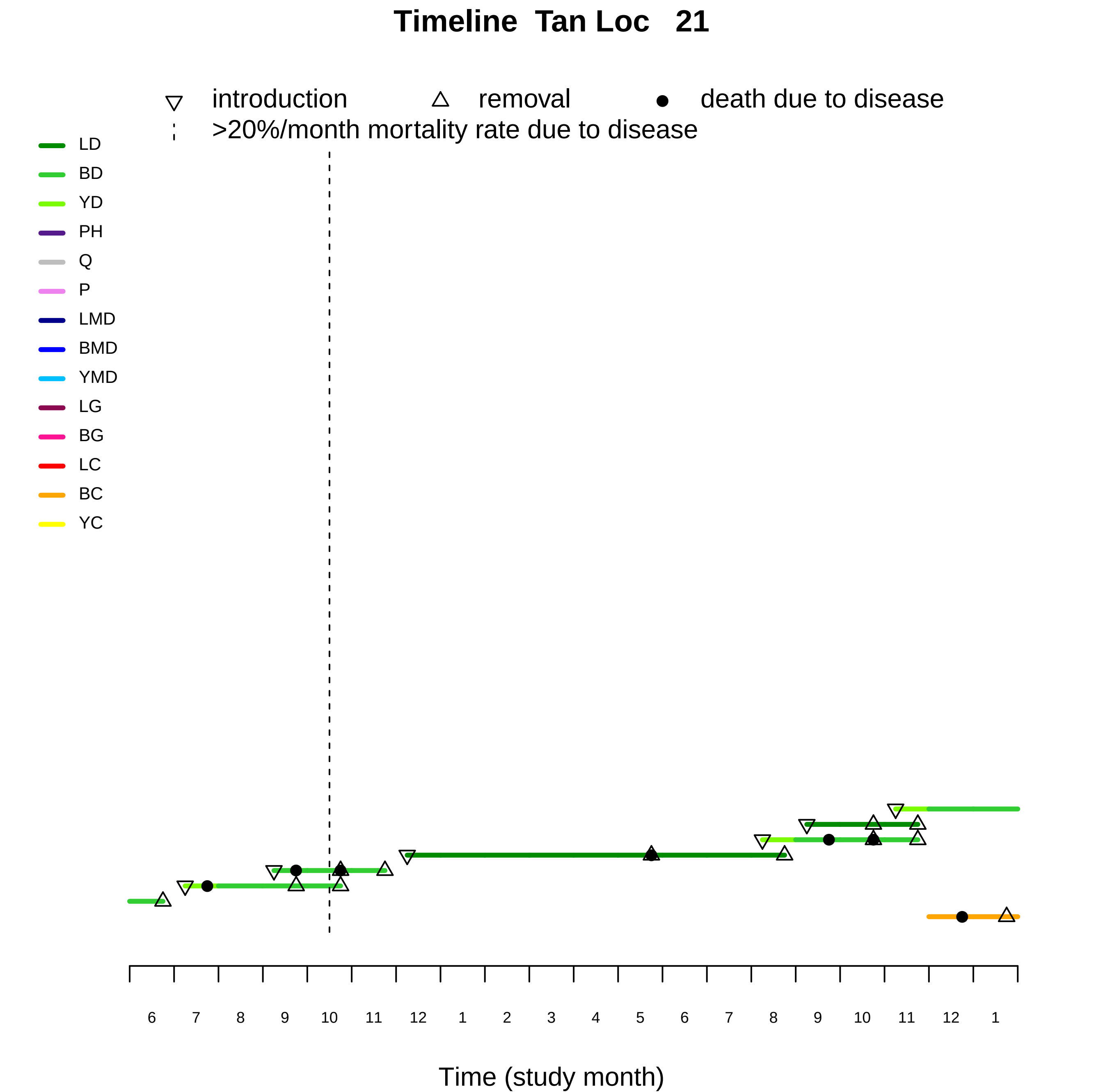

Timeline Tan Loc 21
introduction
removal
death due to disease
>20%/month mortality rate due to disease
LD
BD
YD
PH
Q
P
LMD
BMD
YMD
LG
BG
LC
BC
YC
6
7
8
9
10
11
12
1
2
3
4
5
6
7
8
9
10
11
12
1
Time (study month)

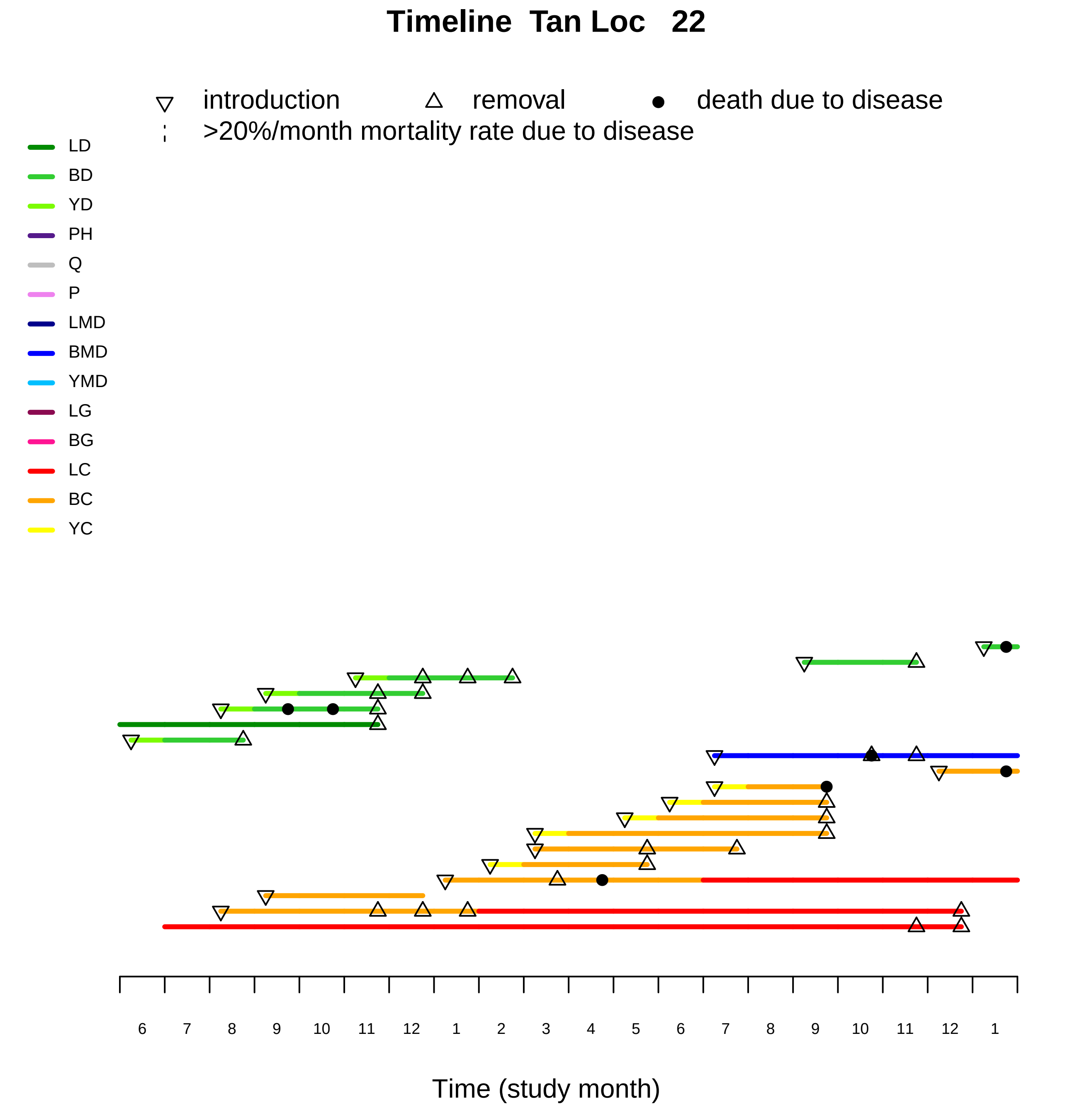

Timeline Tan Loc 22
introduction
removal
death due to disease
>20%/month mortality rate due to disease
LD
BD
YD
PH
Q
P
LMD
BMD
YMD
LG
BG
LC
BC
YC
6
7
8
9
10
11
12
1
2
3
4
5
6
7
8
9
10
11
12
1
Time (study month)

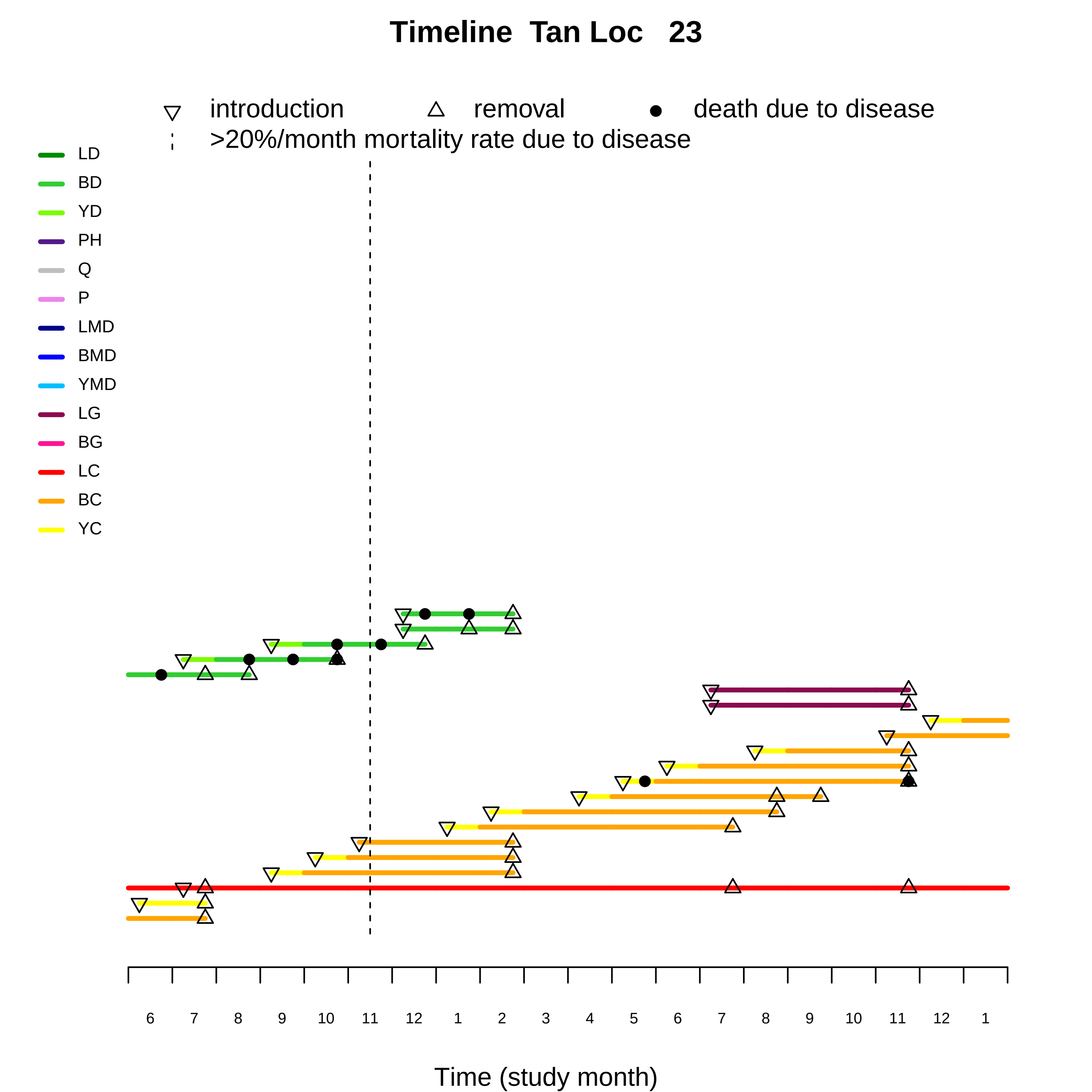
Timeline Tan Loc 23
introduction
removal
death due to disease
>20%/month mortality rate due to disease
LD
BD
YD
PH
Q
P
LMD
BMD
YMD
LG
BG
LC
BC
YC
6
7
8
9
10
11
12
1
2
3
4
5
6
7
8
9
10
11
12
1
Time (study month)

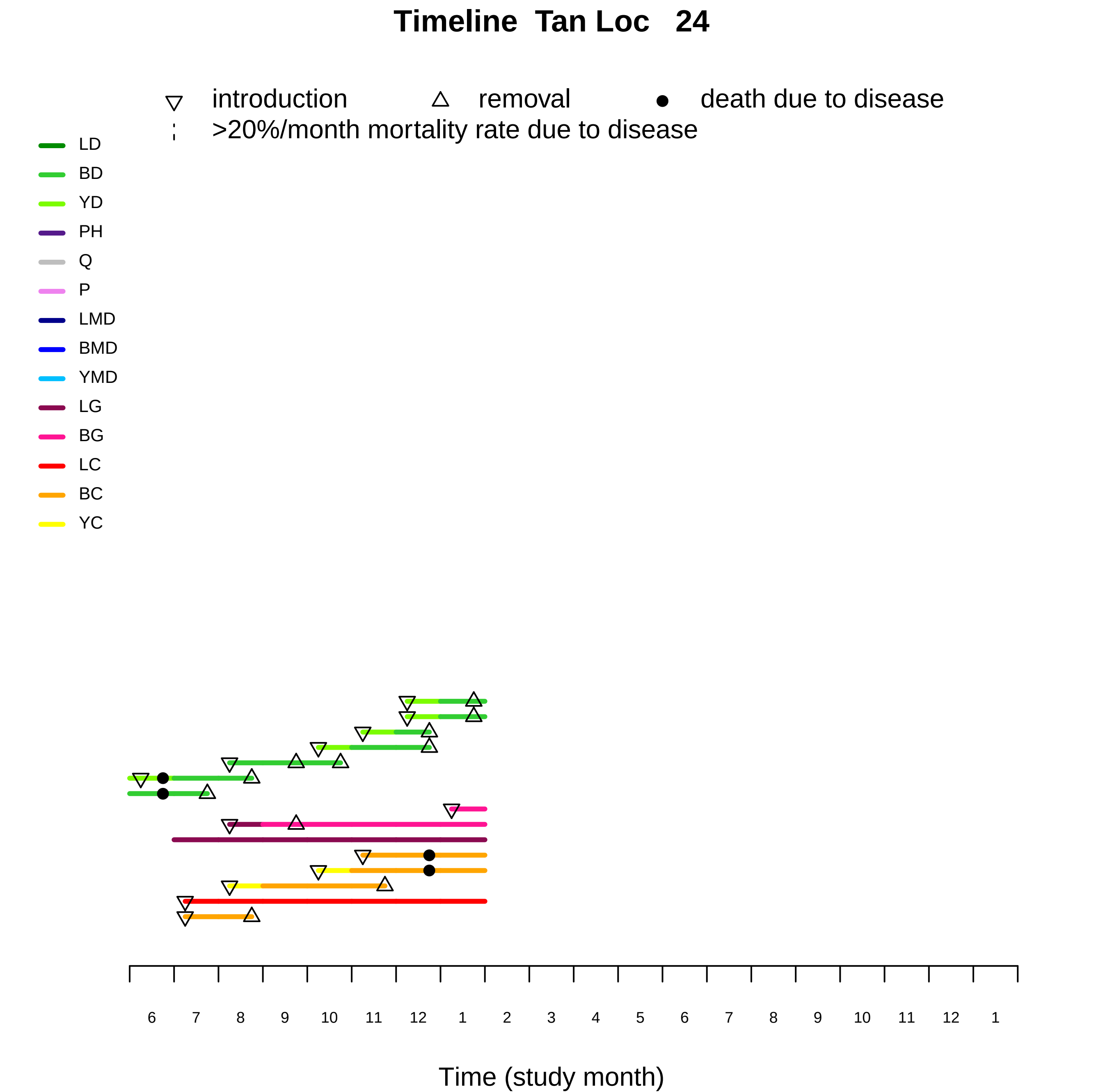
Timeline Tan Loc 24
introduction
removal
death due to disease
>20%/month mortality rate due to disease
LD
BD
YD
PH
Q
P
LMD
BMD
YMD
LG
BG
LC
BC
YC
6
7
8
9
10
11
12
1
2
3
4
5
6
7
8
9
10
11
12
1
Time (study month)

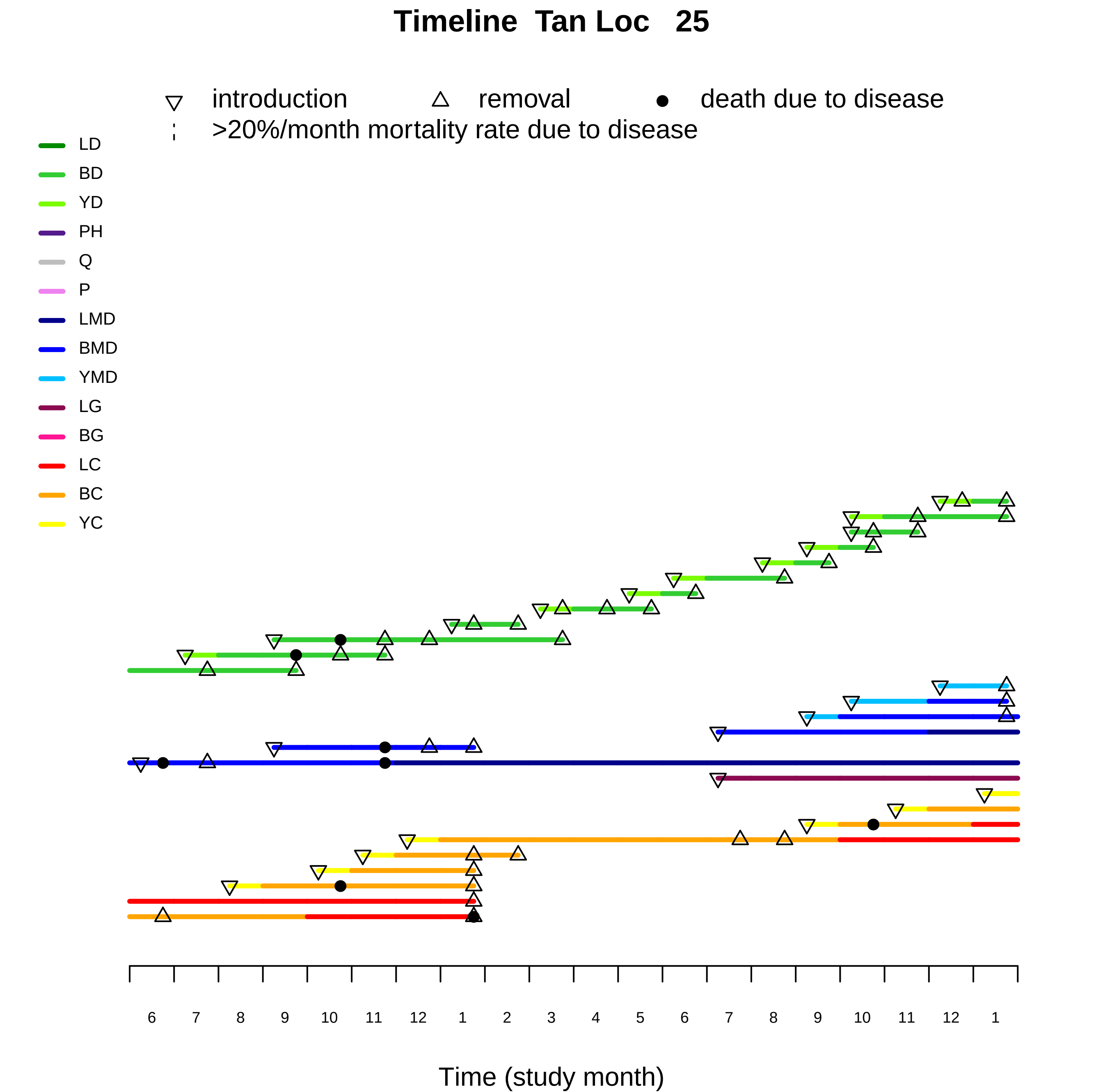
Timeline Tan Loc 25
introduction
removal
death due to disease
>20%/month mortality rate due to disease
LD
BD
YD
PH
Q
P
LMD
BMD
YMD
LG
BG
LC
BC
YC
6
7
8
9
10
11
12
1
2
3
4
5
6
7
8
9
10
11
12
1
Time (study month)

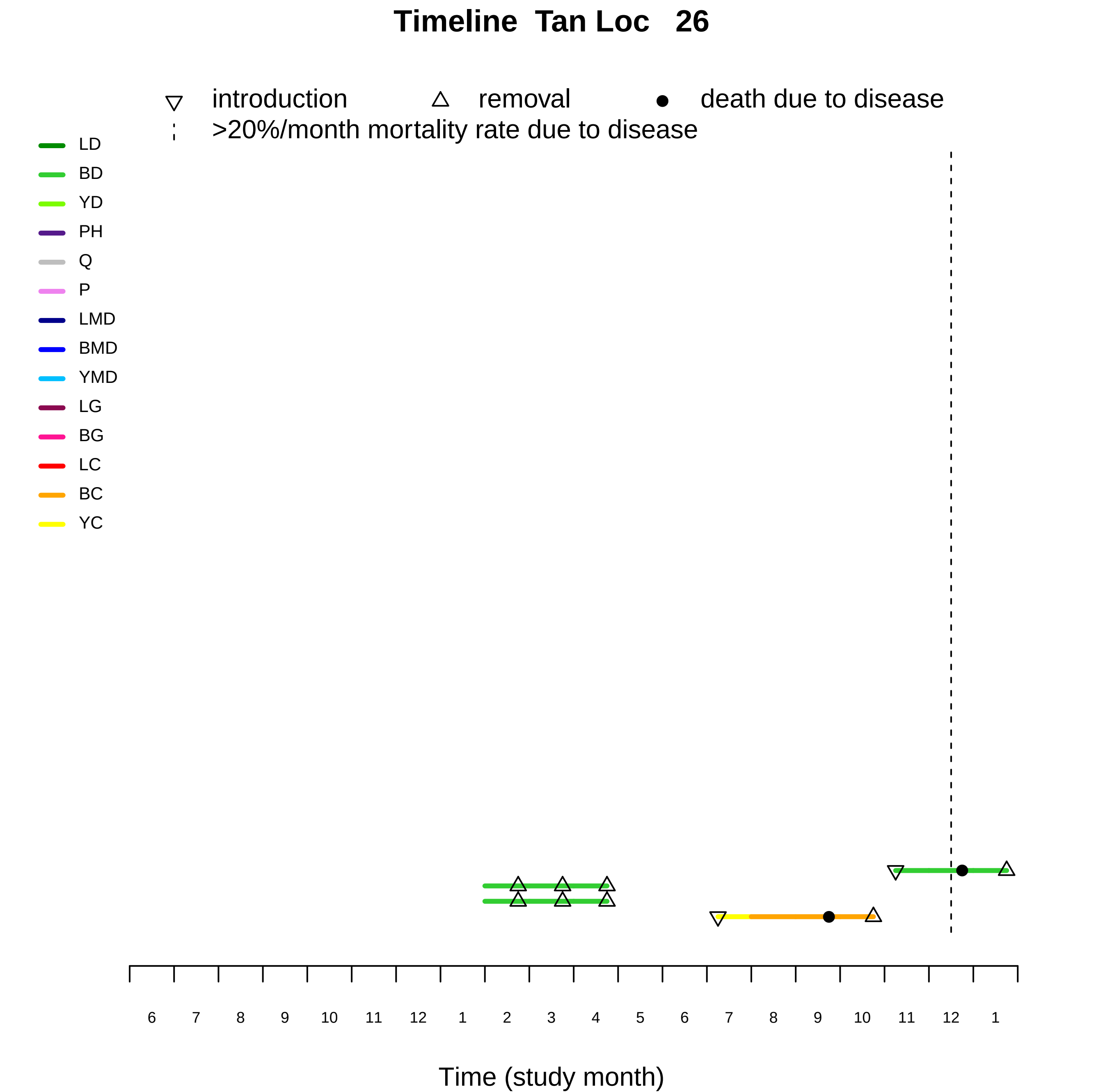

Timeline Tan Loc 26
introduction
removal
death due to disease
>20%/month mortality rate due to disease
LD
BD
YD
PH
Q
P
LMD
BMD
YMD
LG
BG
LC
BC
YC
6
7
8
9
10
11
12
1
2
3
4
5
6
7
8
9
10
11
12
1
Time (study month)

Timeline Tan Phu 1

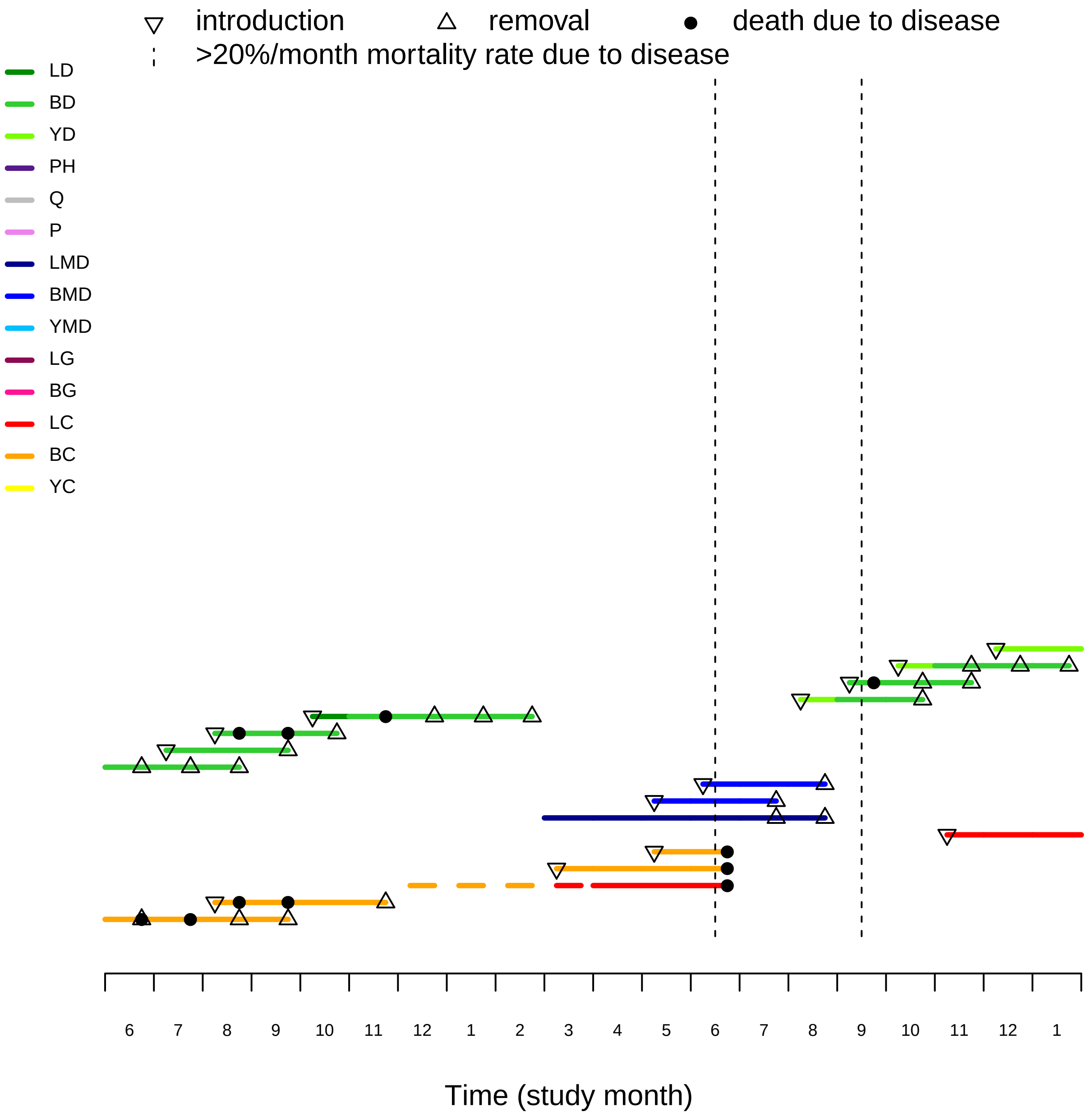

Timeline Tan Phu   2

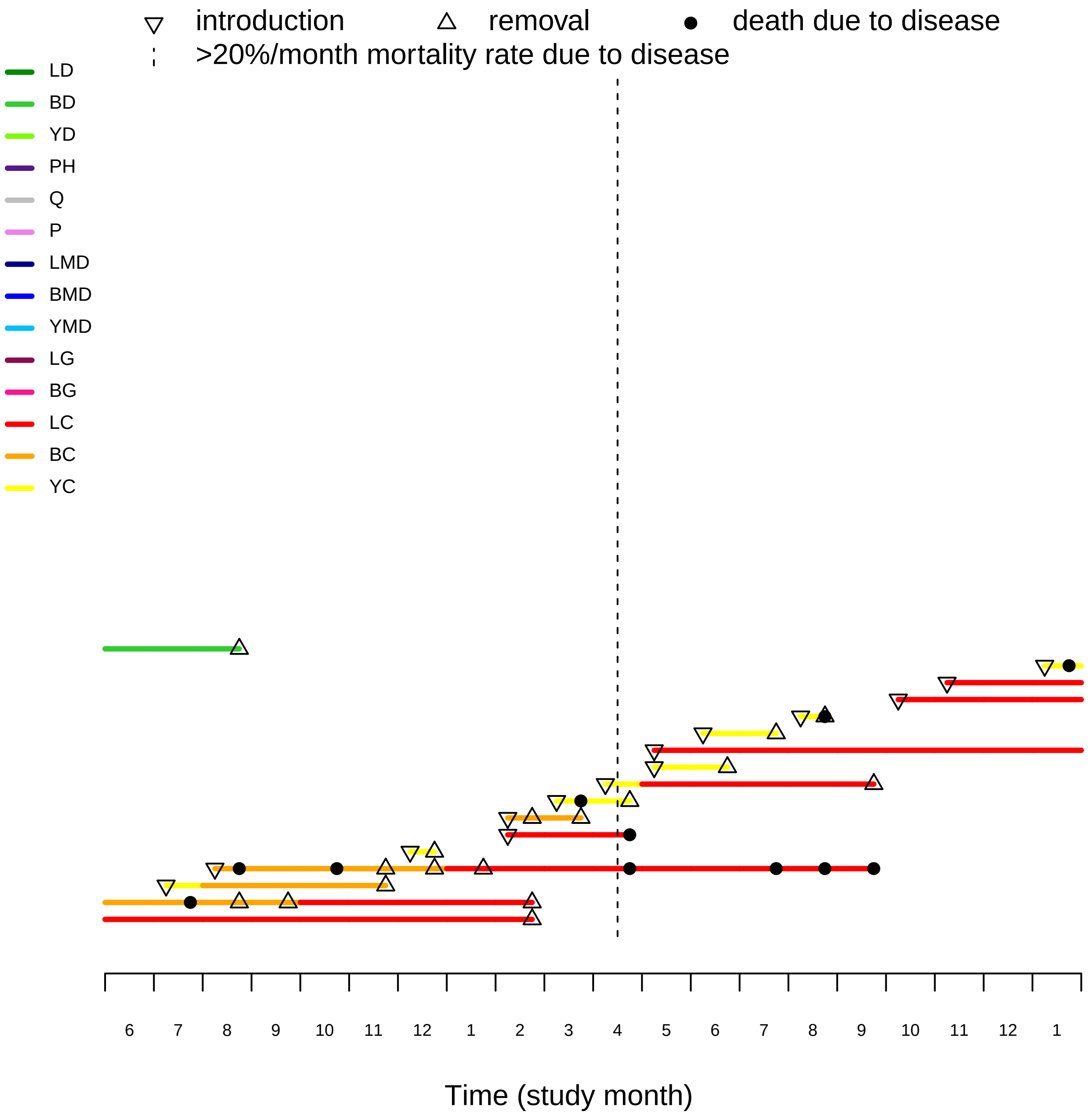

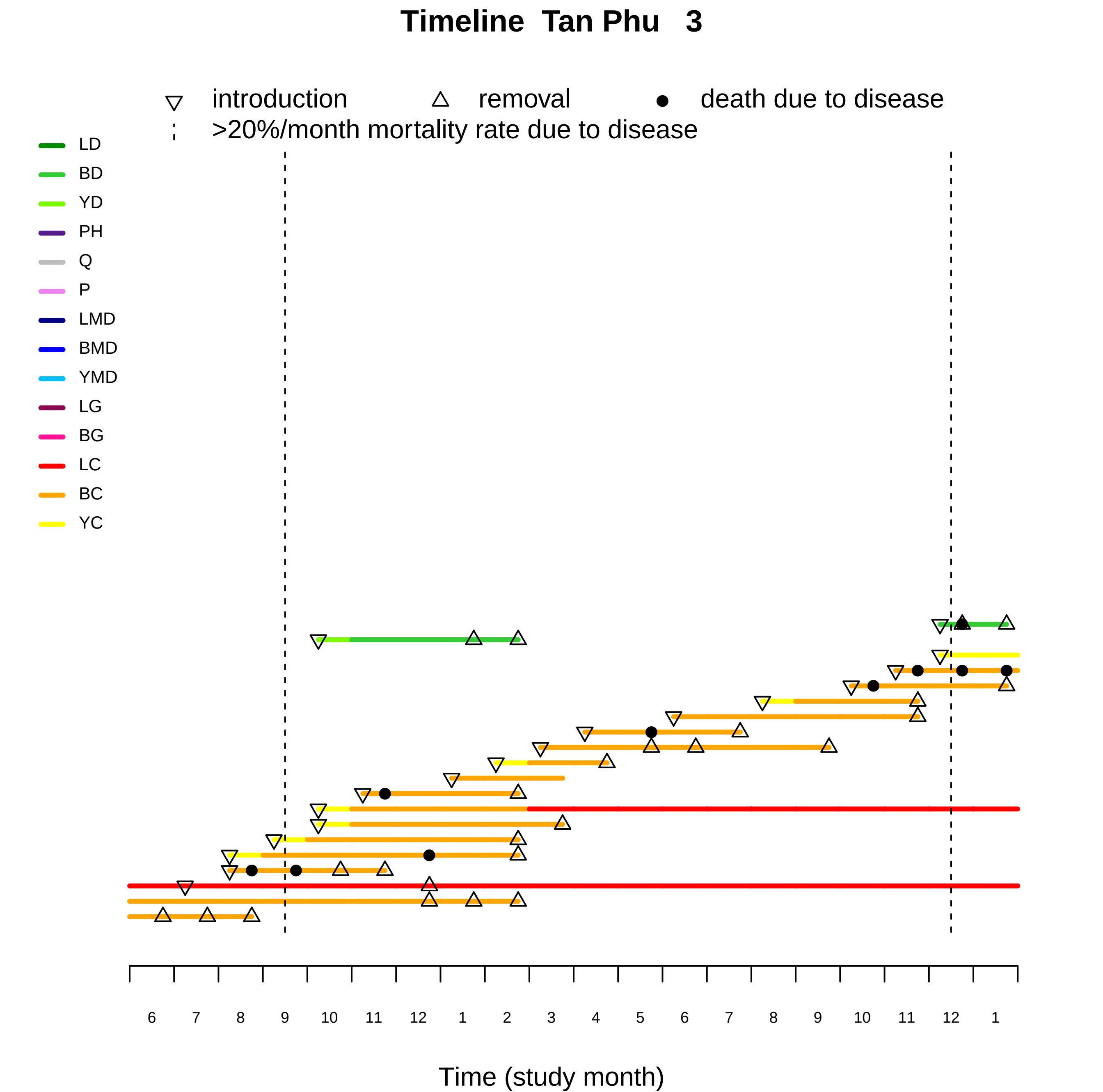
Timeline Tan Phu 3
introduction
removal
death due to disease
>20%/month mortality rate due to disease
LD
BD
YD
PH
Q
P
LMD
BMD
YMD
LG
BG
LC
BC
YC
6
7
8
9
10
11
12
1
2
3
4
5
6
7
8
9
10
11
12
1
Time (study month)

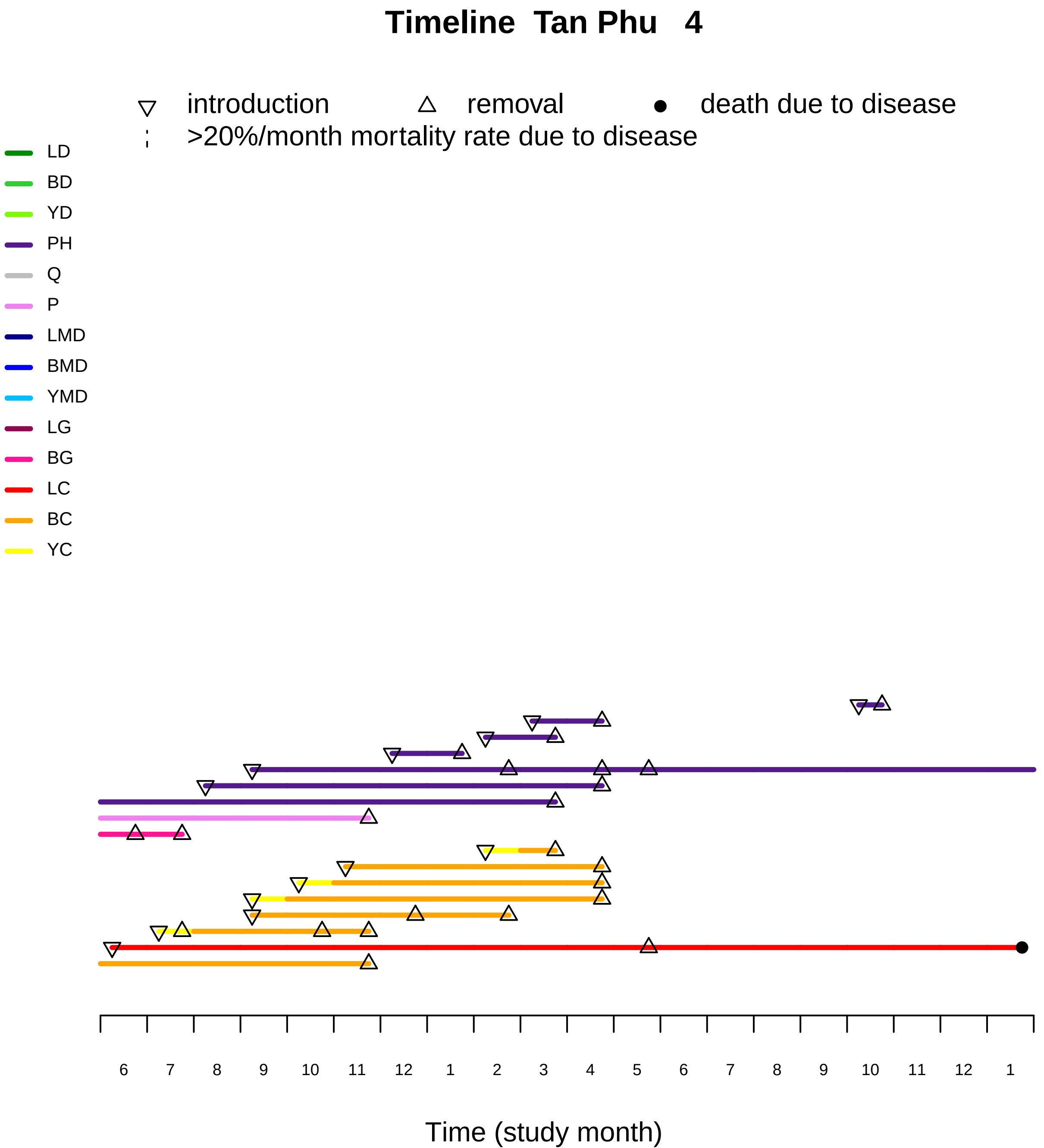
Timeline Tan Phu 4
introduction
removal
death due to disease
>20%/month mortality rate due to disease
LD
BD
YD
PH
Q
P
LMD
BMD
YMD
LG
BG
LC
BC
YC
6
7
8
9
10
11
12
1
2
3
4
5
6
7
8
9
10
11
12
1
Time (study month)

Timeline Tan Phu 5

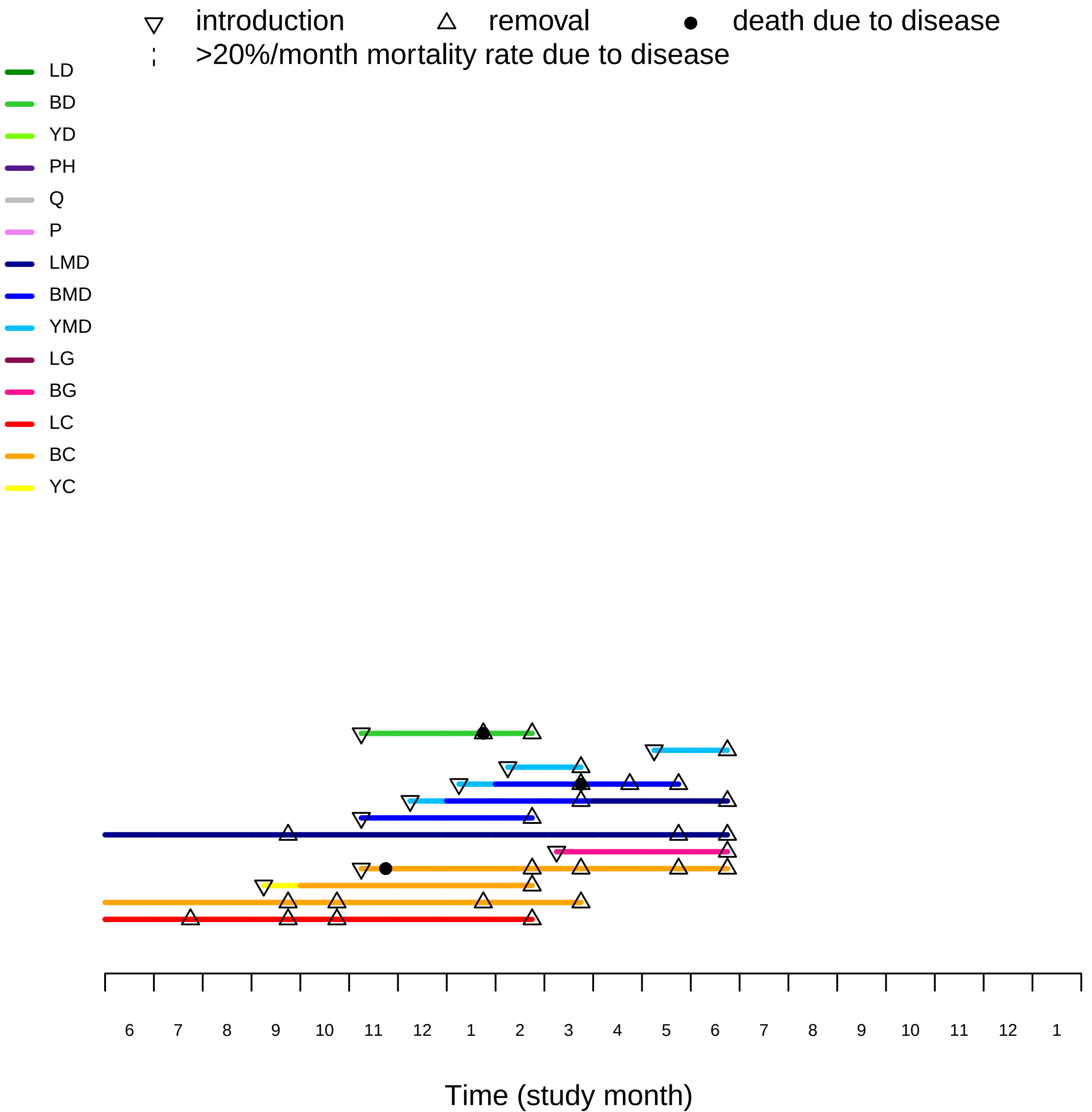

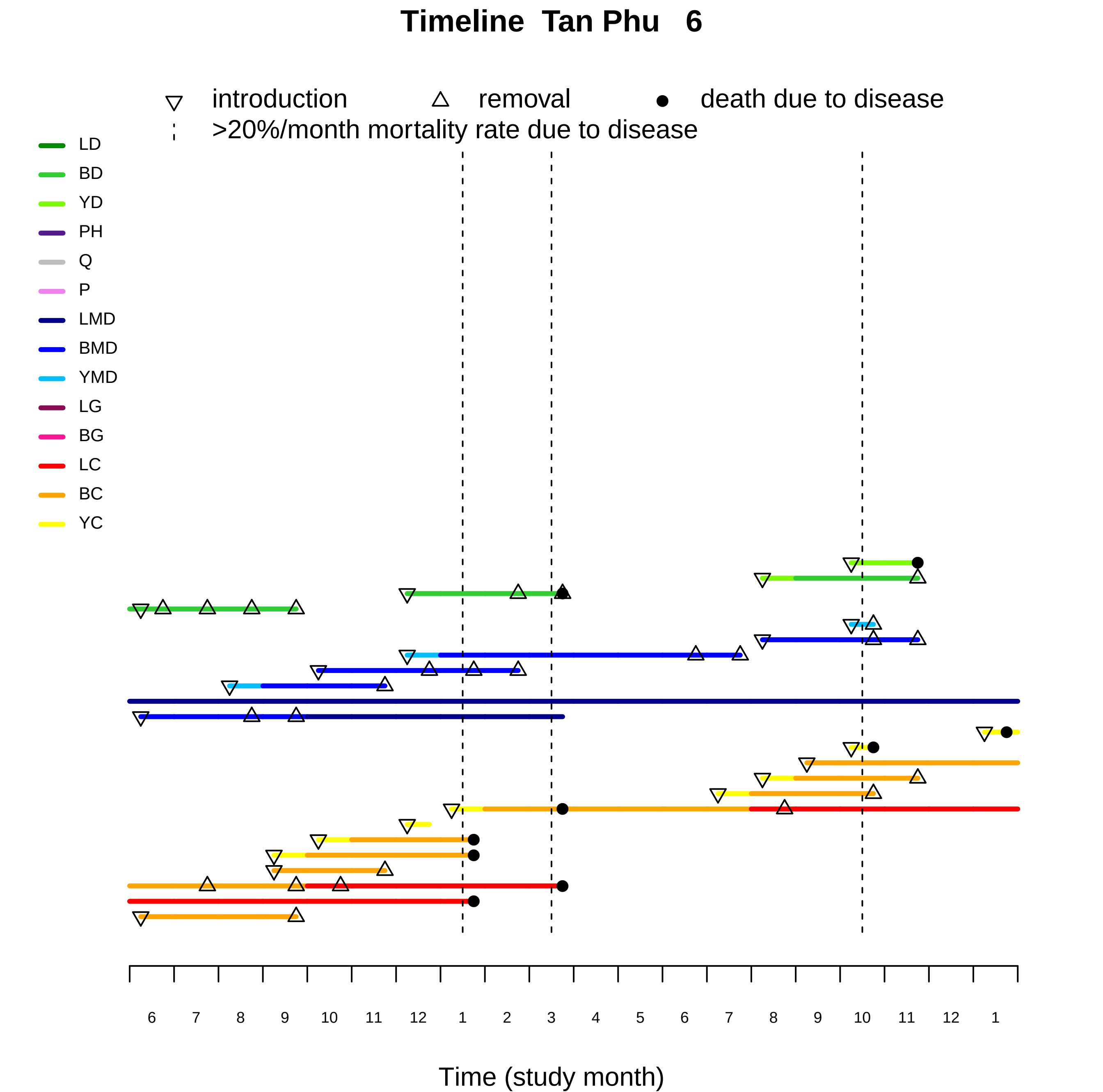

Timeline Tan Phu 6
introduction
removal
death due to disease
>20%/month mortality rate due to disease
LD
BD
YD
PH
Q
P
LMD
BMD
YMD
LG
BG
LC
BC
YC
6
7
8
9
10
11
12
1
2
3
4
5
6
7
8
9
10
11
12
1
Time (study month)

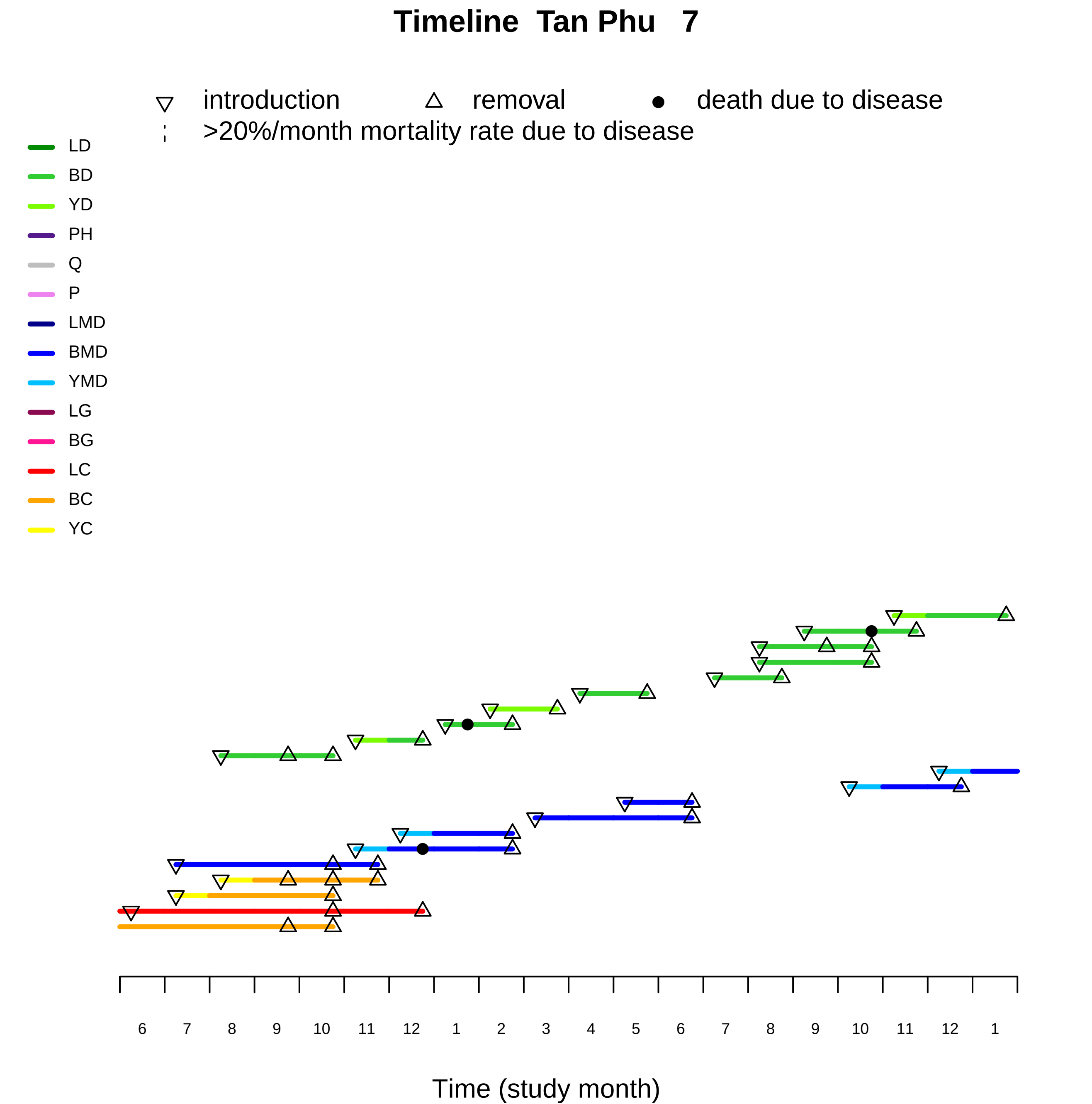

Timeline Tan Phu 7
introduction
removal
death due to disease
>20%/month mortality rate due to disease
LD
BD
YD
PH
Q
P
LMD
BMD
YMD
LG
BG
LC
BC
YC
6
7
8
9
10
11
12
1
2
3
4
5
6
7
8
9
10
11
12
1
Time (study month)

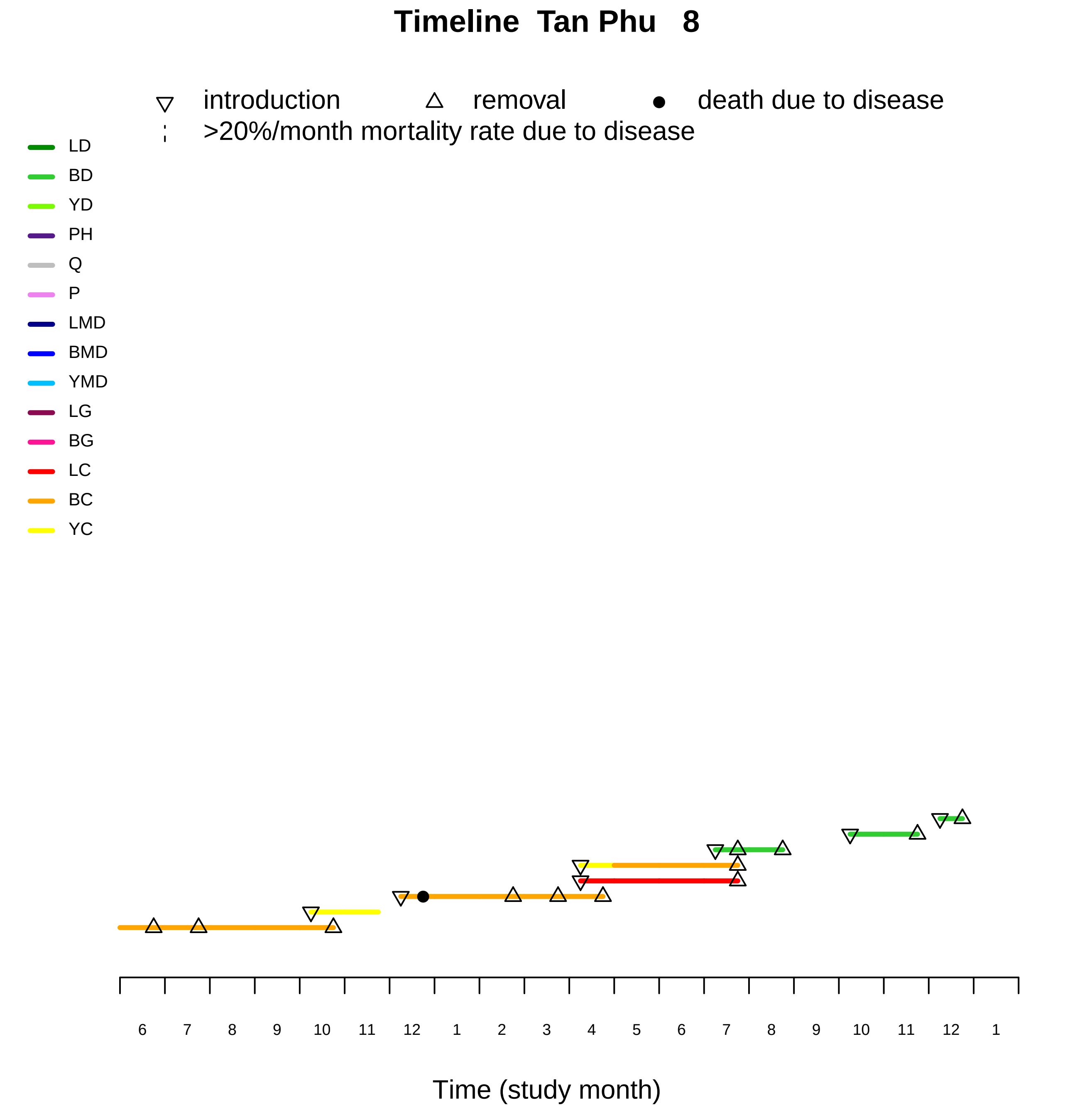
Timeline Tan Phu 8
introduction
removal
death due to disease
>20%/month mortality rate due to disease
LD
BD
YD
PH
Q
P
LMD
BMD
YMD
LG
BG
LC
BC
YC
6
7
8
9
10
11
12
1
2
3
4
5
6
7
8
9
10
11
12
1
Time (study month)

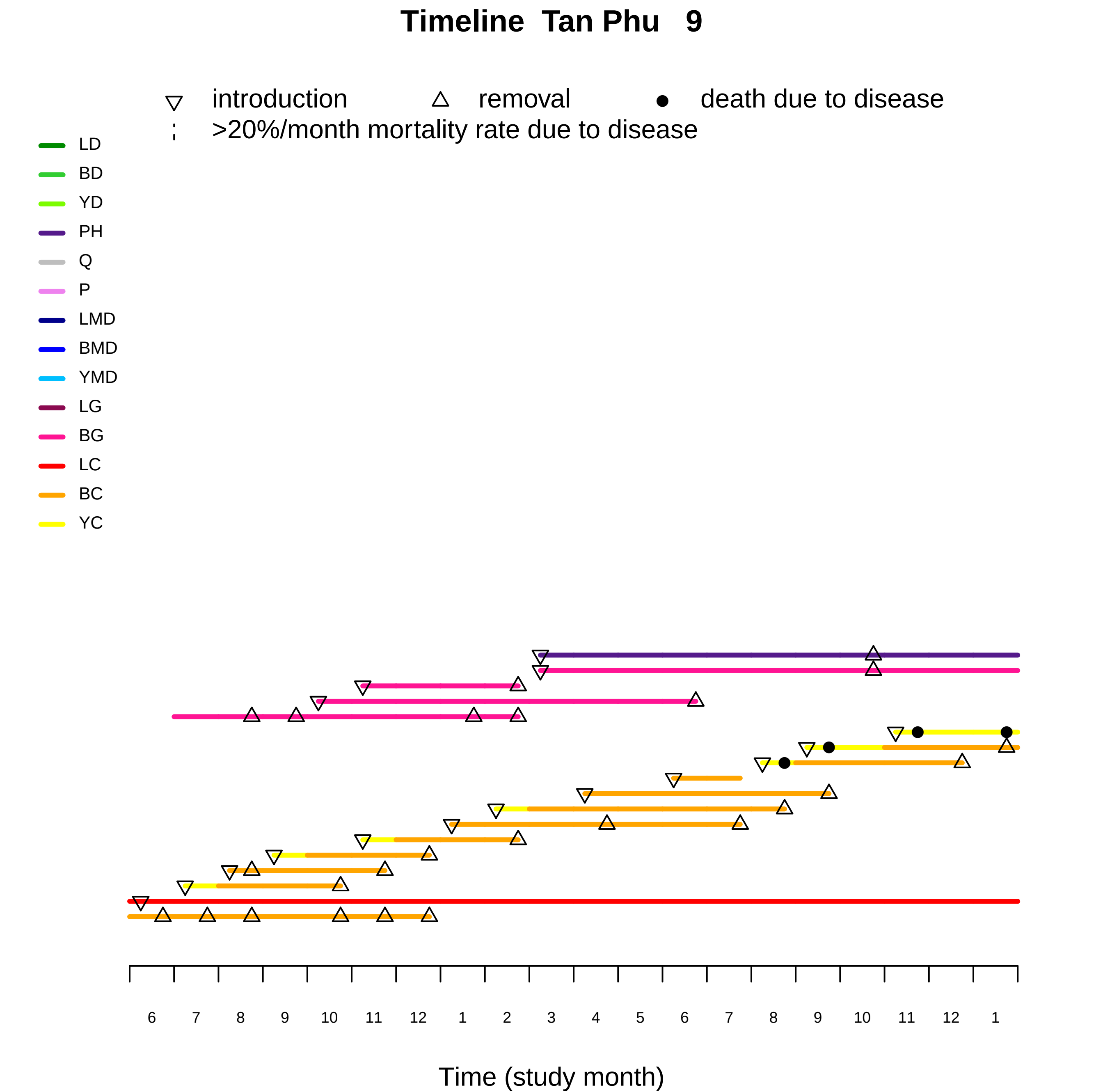
Timeline Tan Phu 9
introduction
removal
death due to disease
>20%/month mortality rate due to disease
LD
BD
YD
PH
Q
P
LMD
BMD
YMD
LG
BG
LC
BC
YC
6
7
8
9
10
11
12
1
2
3
4
5
6
7
8
9
10
11
12
1
Time (study month)

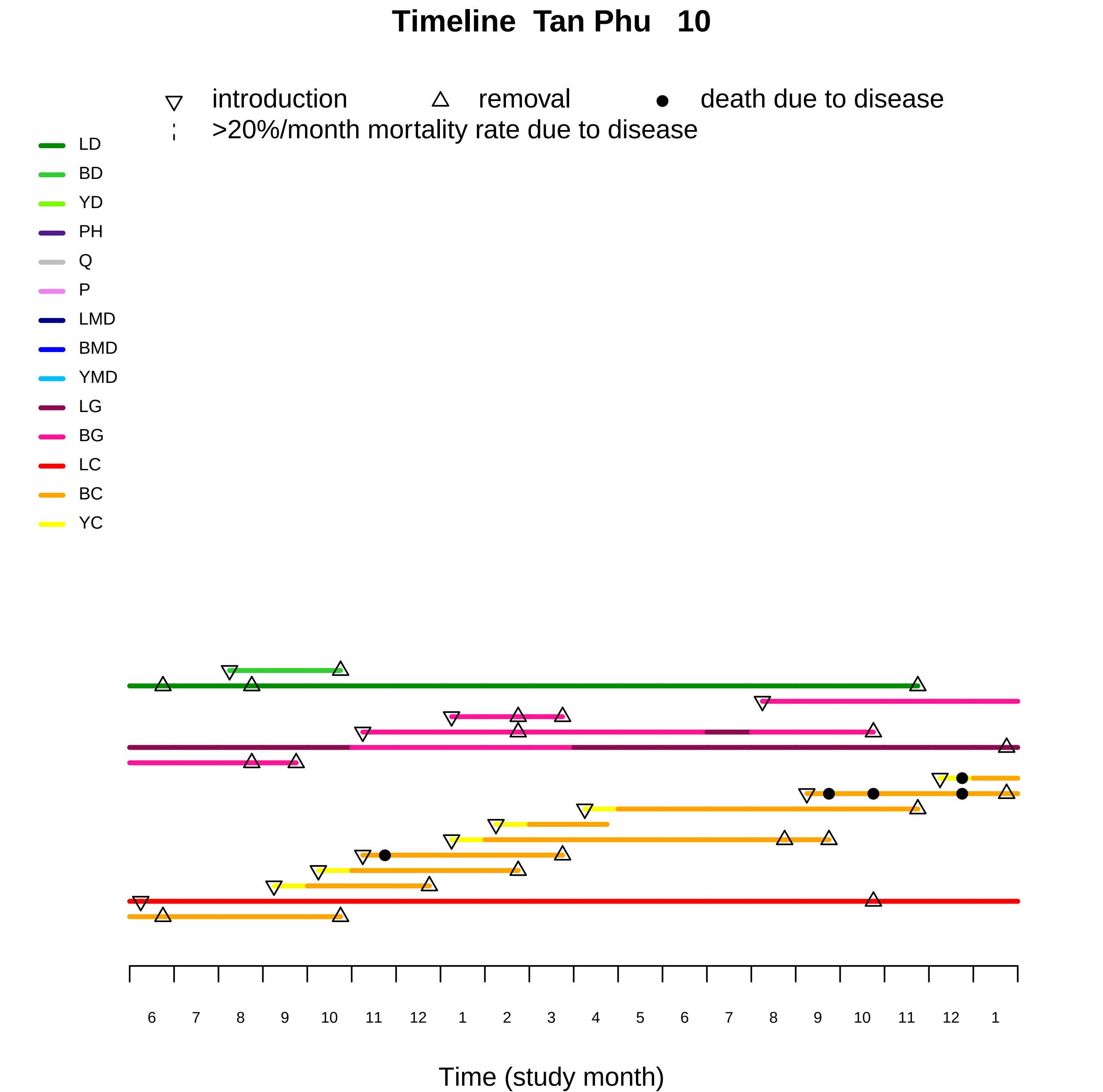

Timeline Tan Phu 10
introduction
removal
death due to disease
>20%/month mortality rate due to disease
LD
BD
YD
PH
Q
P
LMD
BMD
YMD
LG
BG
LC
BC
YC
6 7 8 9 10 11 12 1 2 3 4 5 6 7 8 9 10 11 12 1
Time (study month)

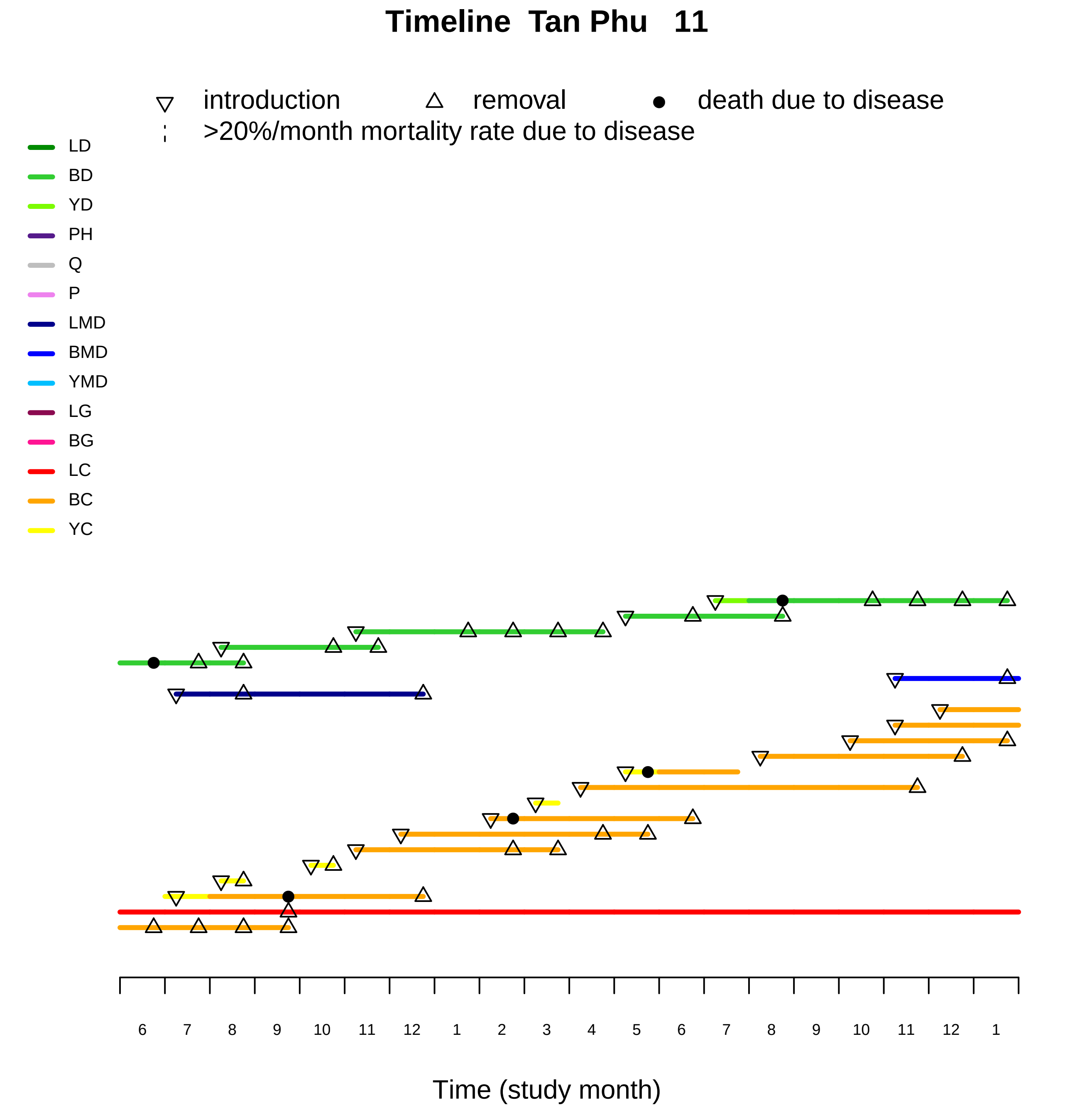

Timeline Tan Phu 11
introduction
removal
death due to disease
>20%/month mortality rate due to disease
LD
BD
YD
PH
Q
P
LMD
BMD
YMD
LG
BG
LC
BC
YC
6
7
8
9
10
11
12
1
2
3
4
5
6
7
8
9
10
11
12
1
Time (study month)

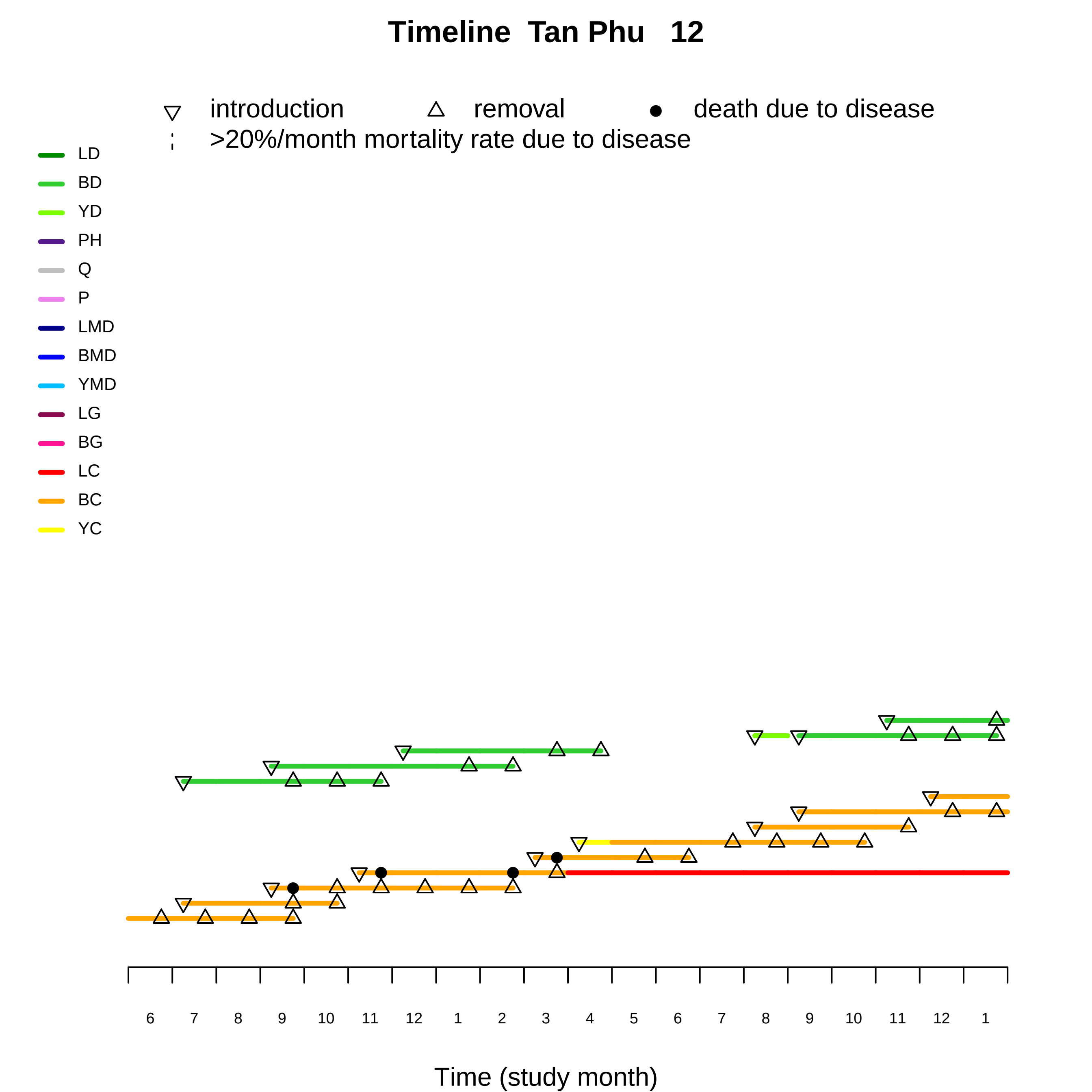

Timeline Tan Phu 12
introduction
removal
death due to disease
>20%/month mortality rate due to disease
LD
BD
YD
PH
Q
P
LMD
BMD
YMD
LG
BG
LC
BC
YC
6
7
8
9
10
11
12
1
2
3
4
5
6
7
8
9
10
11
12
1
Time (study month)

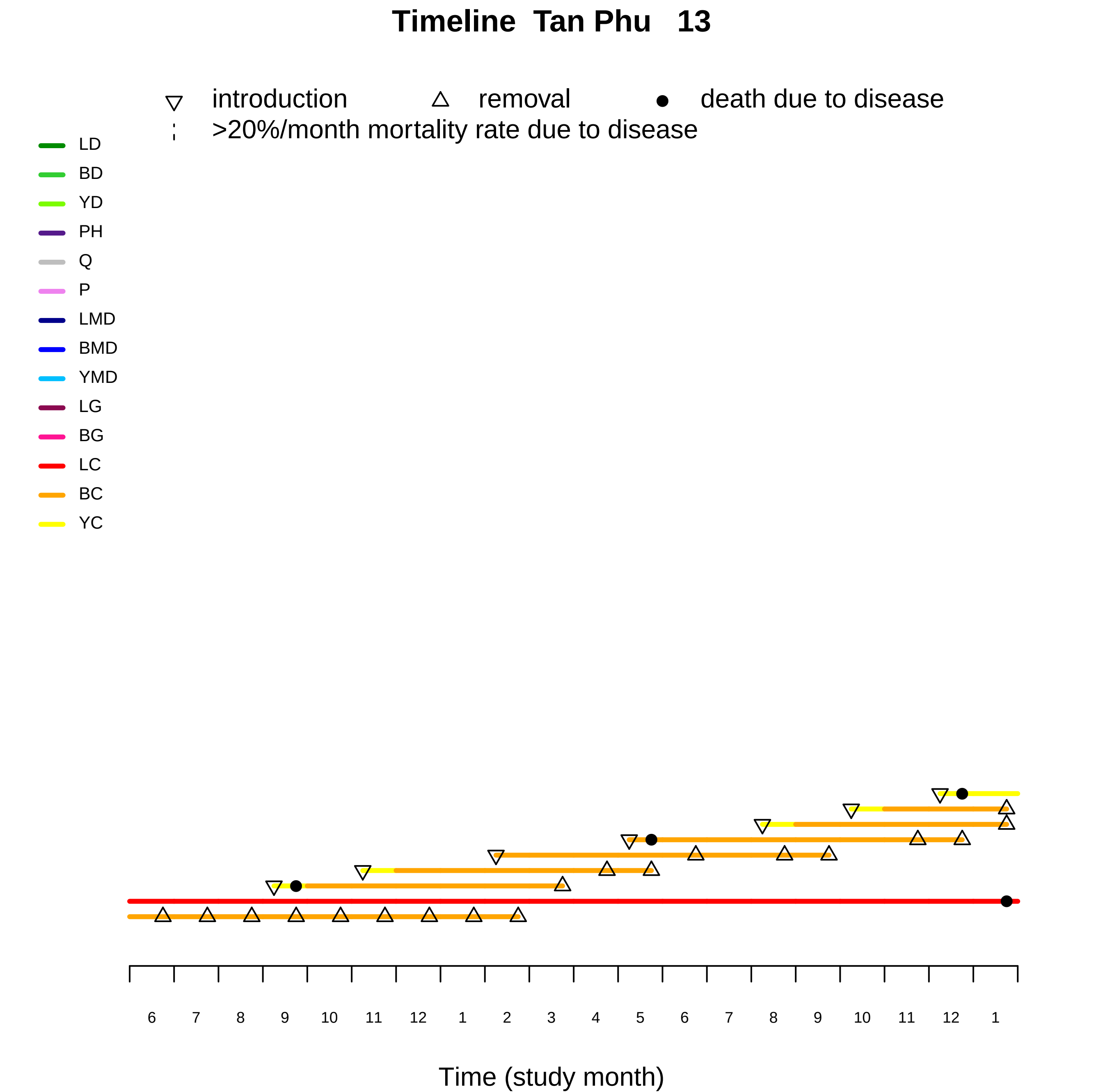
Timeline Tan Phu 13
introduction
removal
death due to disease
>20%/month mortality rate due to disease
LD
BD
YD
PH
Q
P
LMD
BMD
YMD
LG
BG
LC
BC
YC
6 7 8 9 10 11 12 1 2 3 4 5 6 7 8 9 10 11 12 1
Time (study month)

Timeline Tan Phu 14

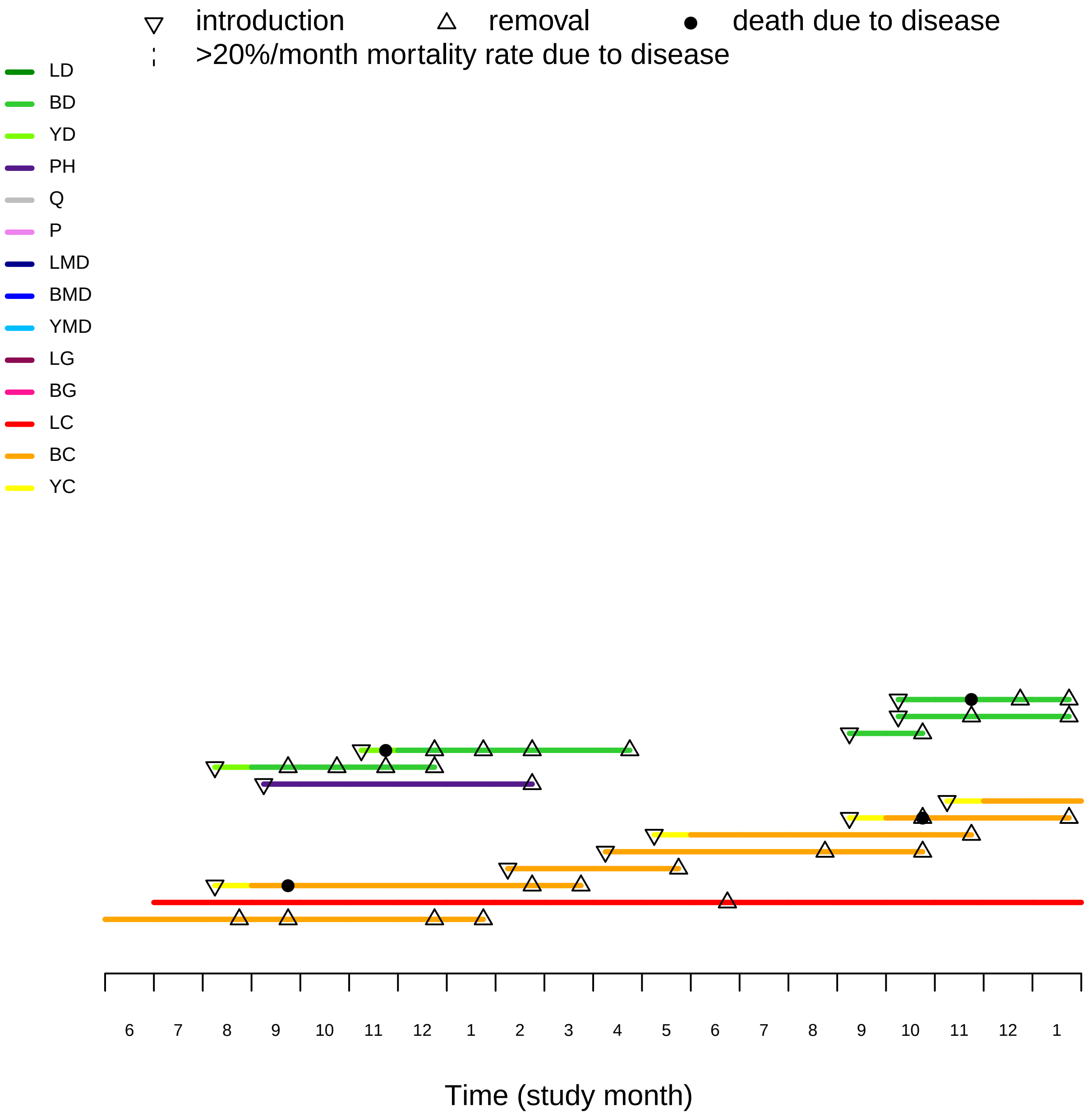

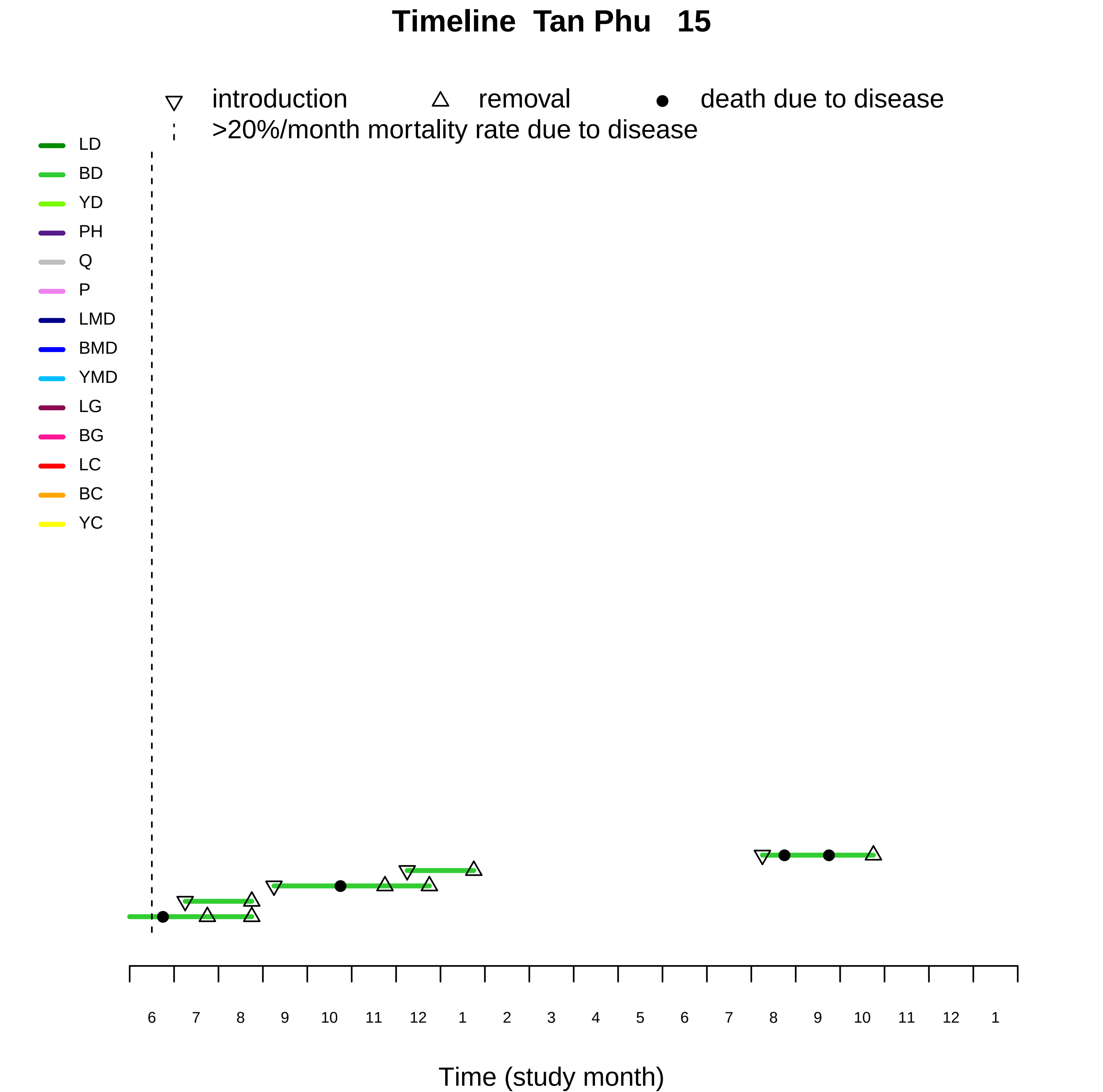
Timeline Tan Phu 15
introduction
removal
death due to disease
>20%/month mortality rate due to disease
LD
BD
YD
PH
Q
P
LMD
BMD
YMD
LG
BG
LC
BC
YC
6 7 8 9 10 11 12 1 2 3 4 5 6 7 8 9 10 11 12 1
Time (study month)

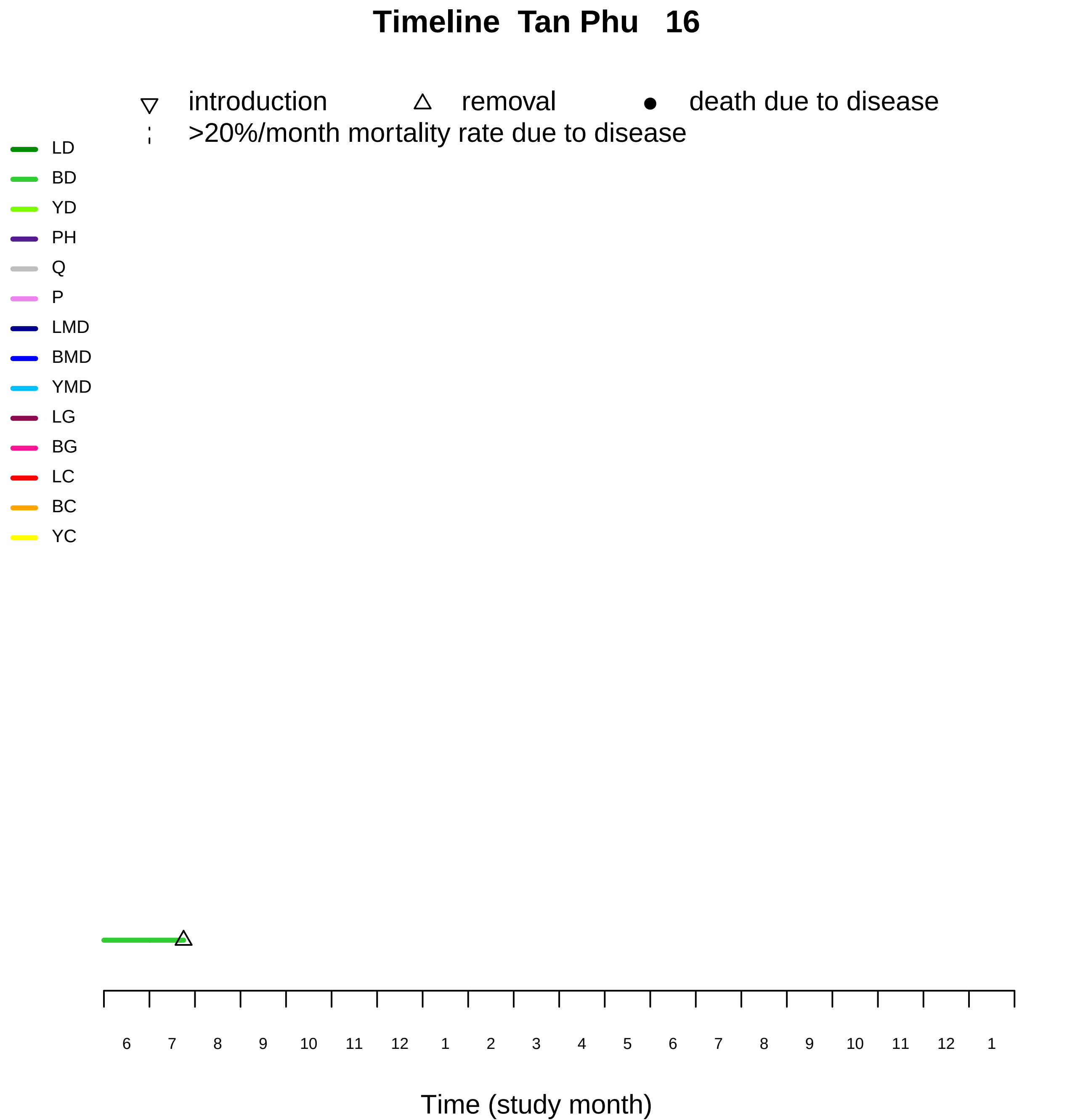

Timeline Tan Phu 16
introduction
removal
death due to disease
>20%/month mortality rate due to disease
LD
BD
YD
PH
Q
P
LMD
BMD
YMD
LG
BG
LC
BC
YC
6 7 8 9 10 11 12 1 2 3 4 5 6 7 8 9 10 11 12 1
Time (study month)

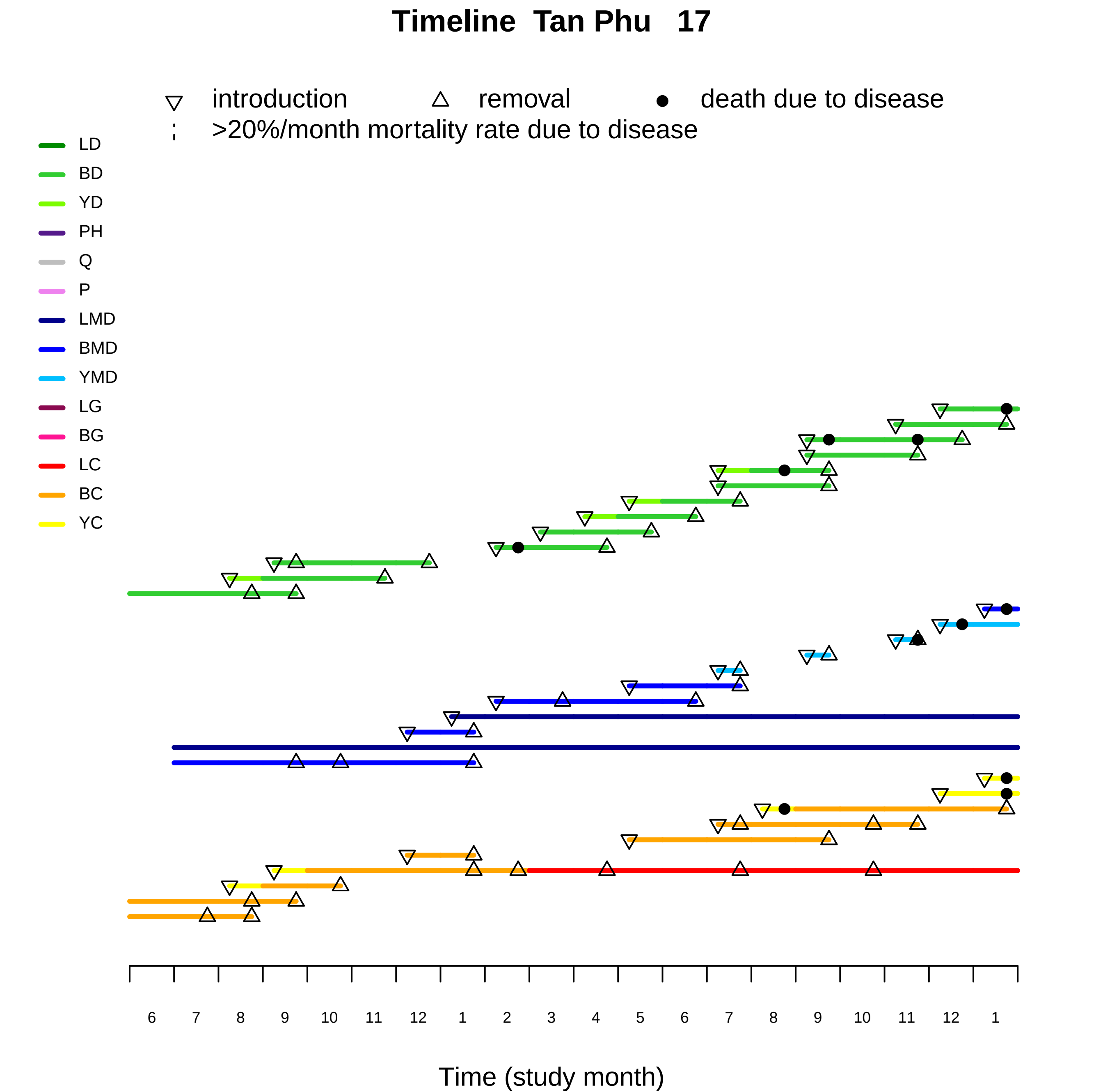
Timeline Tan Phu 17
introduction
removal
death due to disease
>20%/month mortality rate due to disease
LD
BD
YD
PH
Q
P
LMD
BMD
YMD
LG
BG
LC
BC
YC
6 7 8 9 10 11 12 1 2 3 4 5 6 7 8 9 10 11 12 1
Time (study month)

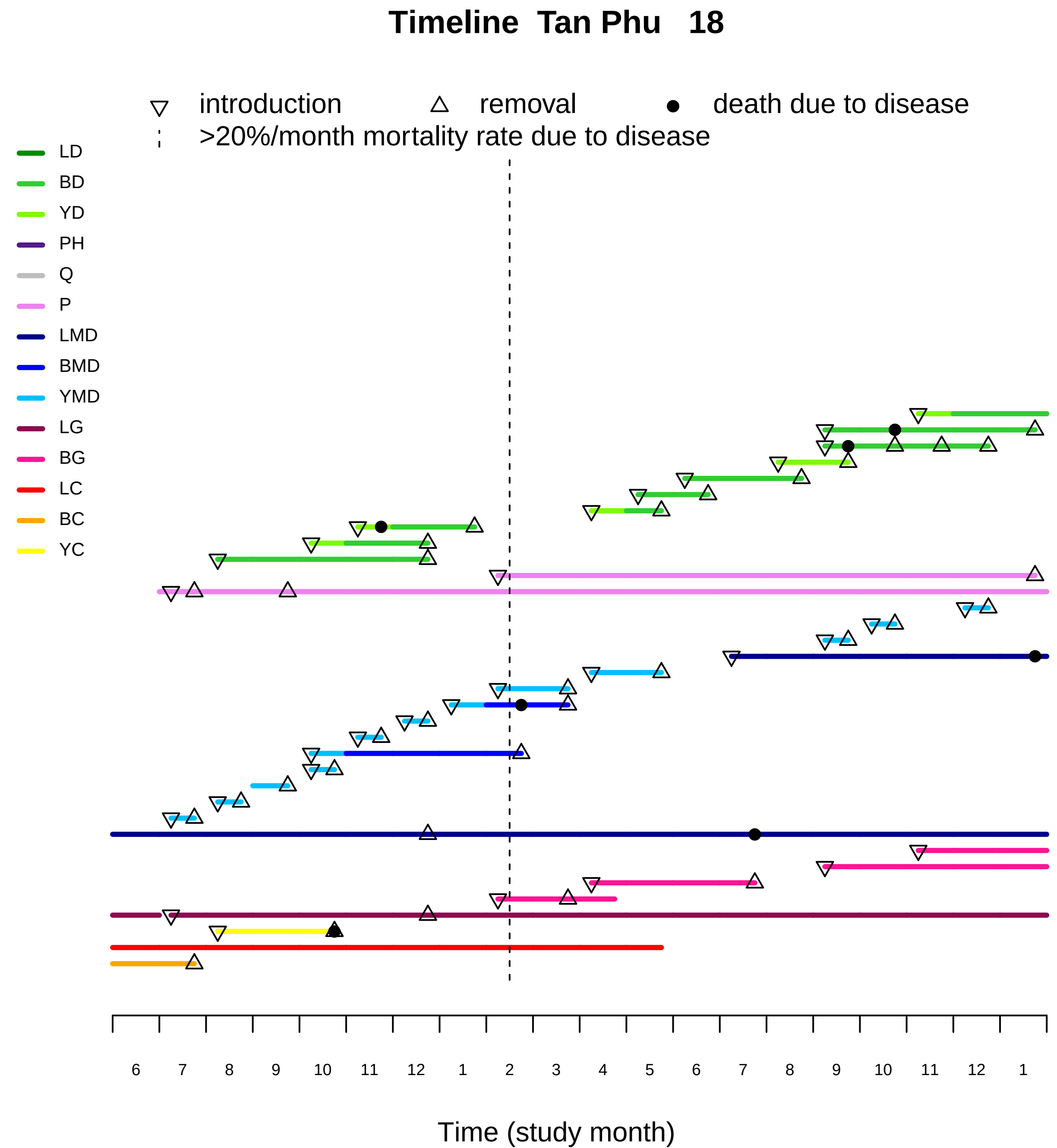

Timeline Tan Phu 18
introduction
removal
death due to disease
>20%/month mortality rate due to disease
LD
BD
YD
PH
Q
P
LMD
BMD
YMD
LG
BG
LC
BC
YC
6
7
8
9
10
11
12
1
2
3
4
5
6
7
8
9
10
11
12
1
Time (study month)

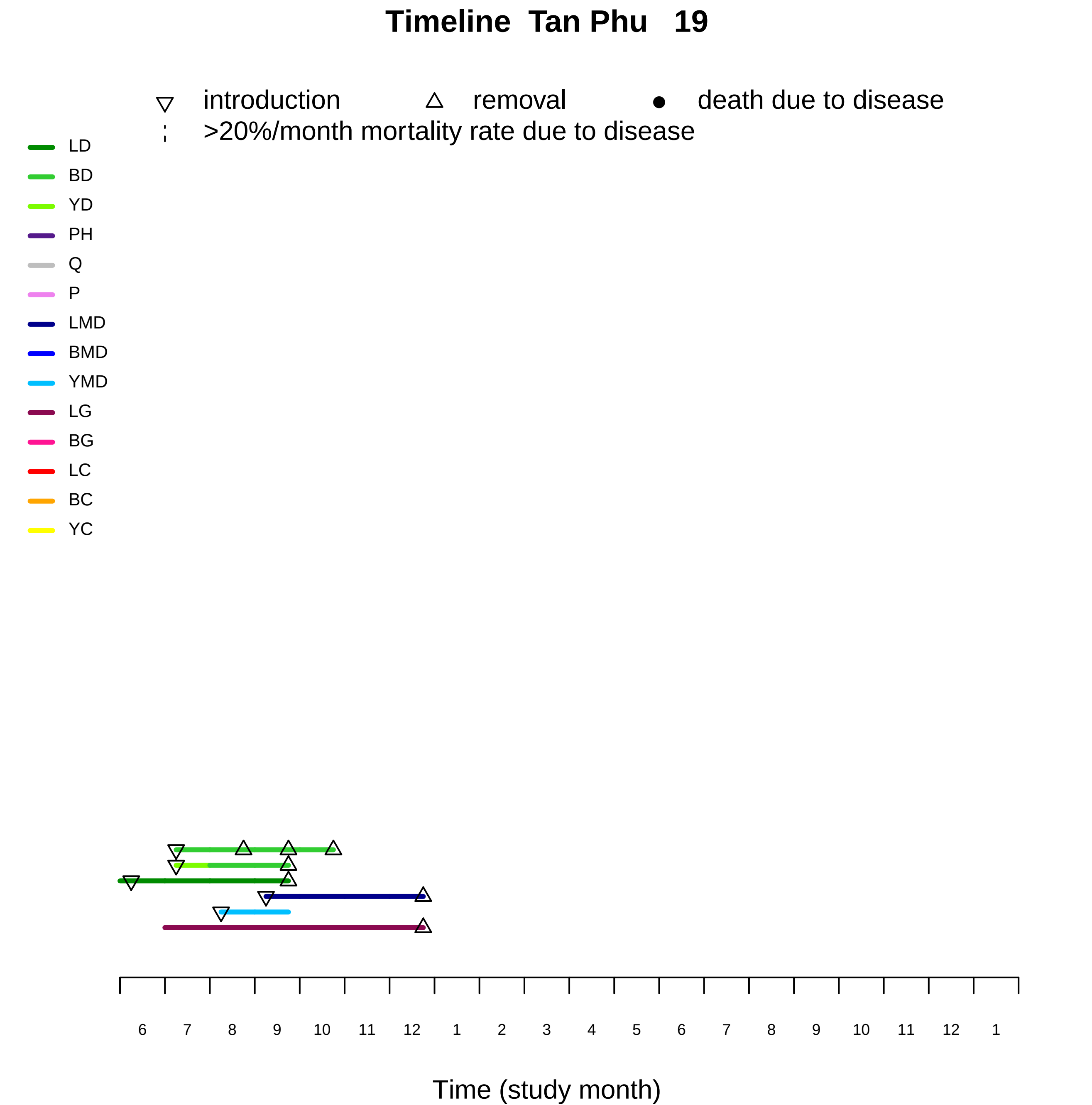

Timeline Tan Phu 19
introduction
removal
death due to disease
>20%/month mortality rate due to disease
LD
BD
YD
PH
Q
P
LMD
BMD
YMD
LG
BG
LC
BC
YC
6
7
8
9
10
11
12
1
2
3
4
5
6
7
8
9
10
11
12
1
Time (study month)

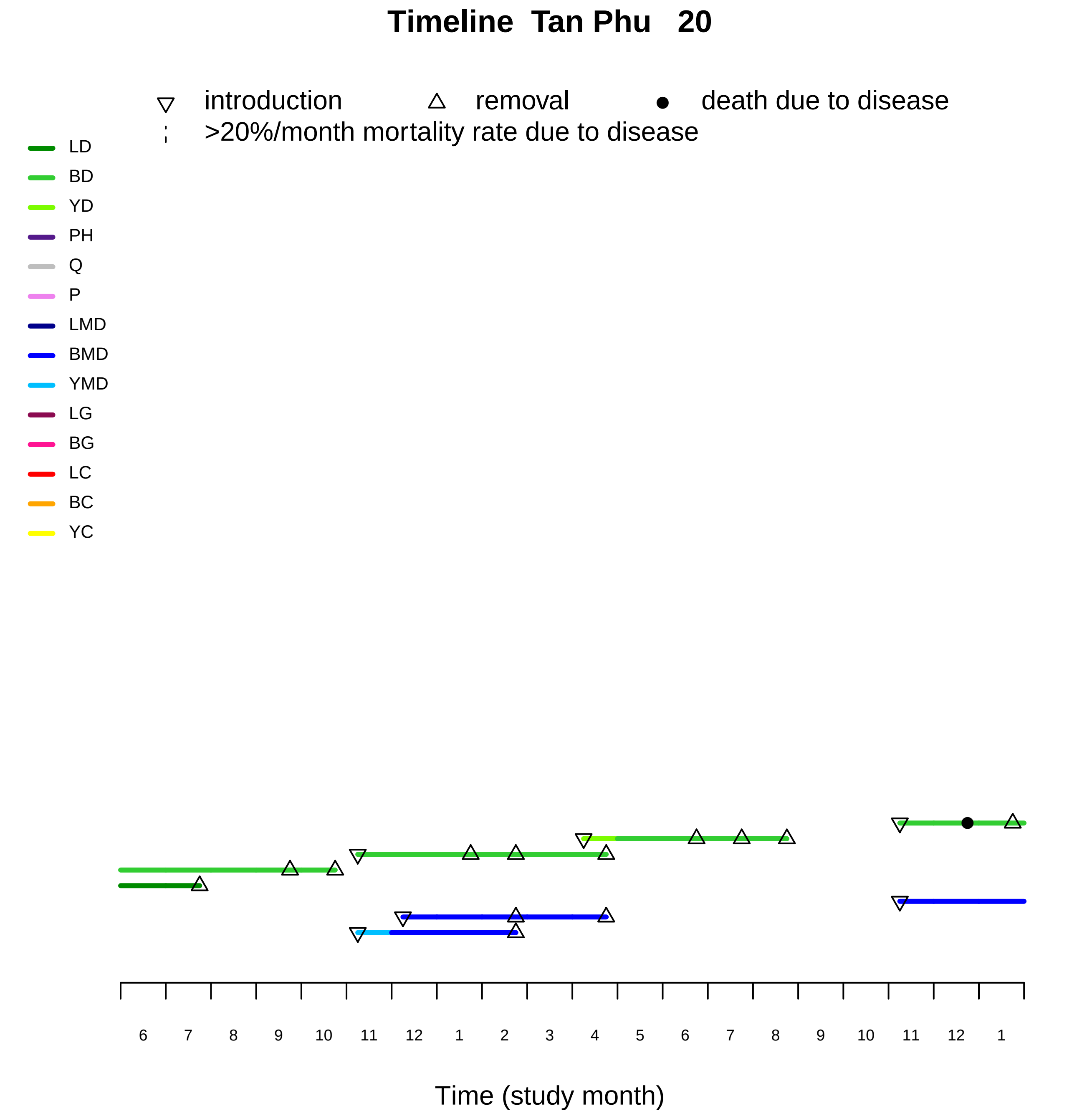
Timeline Tan Phu 20
introduction
removal
death due to disease
>20%/month mortality rate due to disease
LD
BD
YD
PH
Q
P
LMD
BMD
YMD
LG
BG
LC
BC
YC
6 7 8 9 10 11 12 1 2 3 4 5 6 7 8 9 10 11 12 1
Time (study month)

Timeline Tan Phu 21

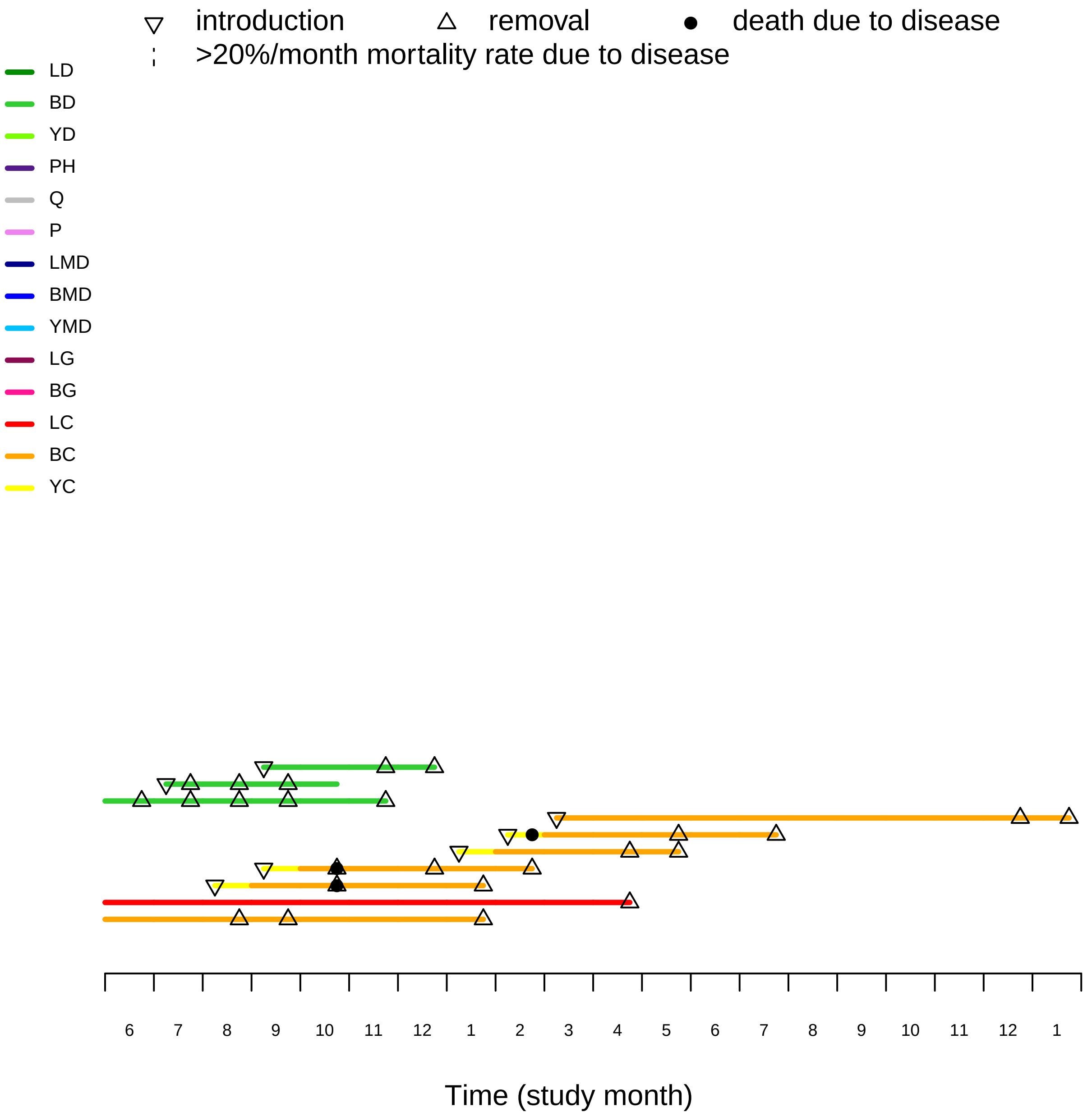

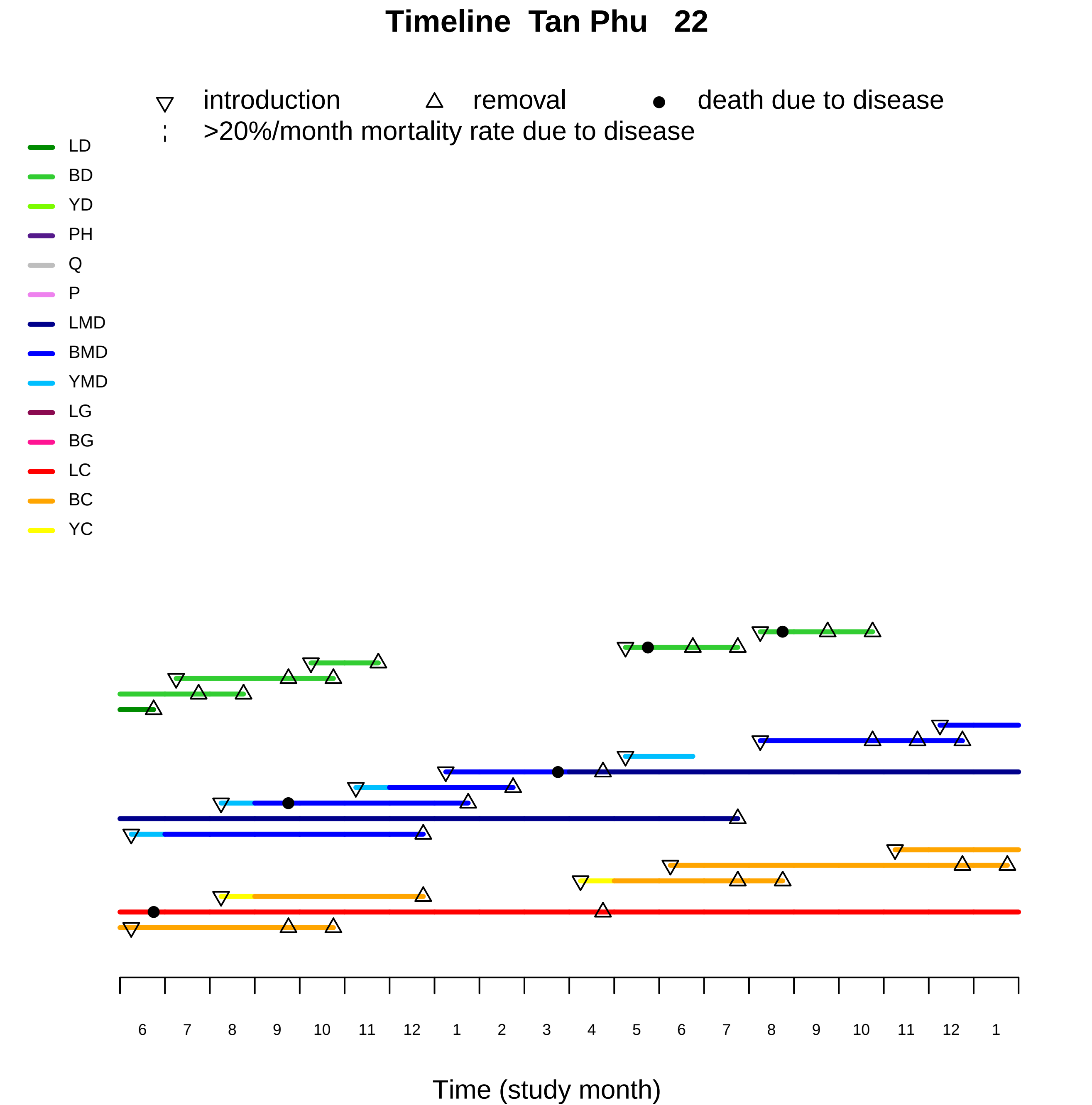

Timeline Tan Phu 22
introduction
removal
death due to disease
>20%/month mortality rate due to disease
LD
BD
YD
PH
Q
P
LMD
BMD
YMD
LG
BG
LC
BC
YC
6
7
8
9
10
11
12
1
2
3
4
5
6
7
8
9
10
11
12
1
Time (study month)

Timeline Tan Phu 23

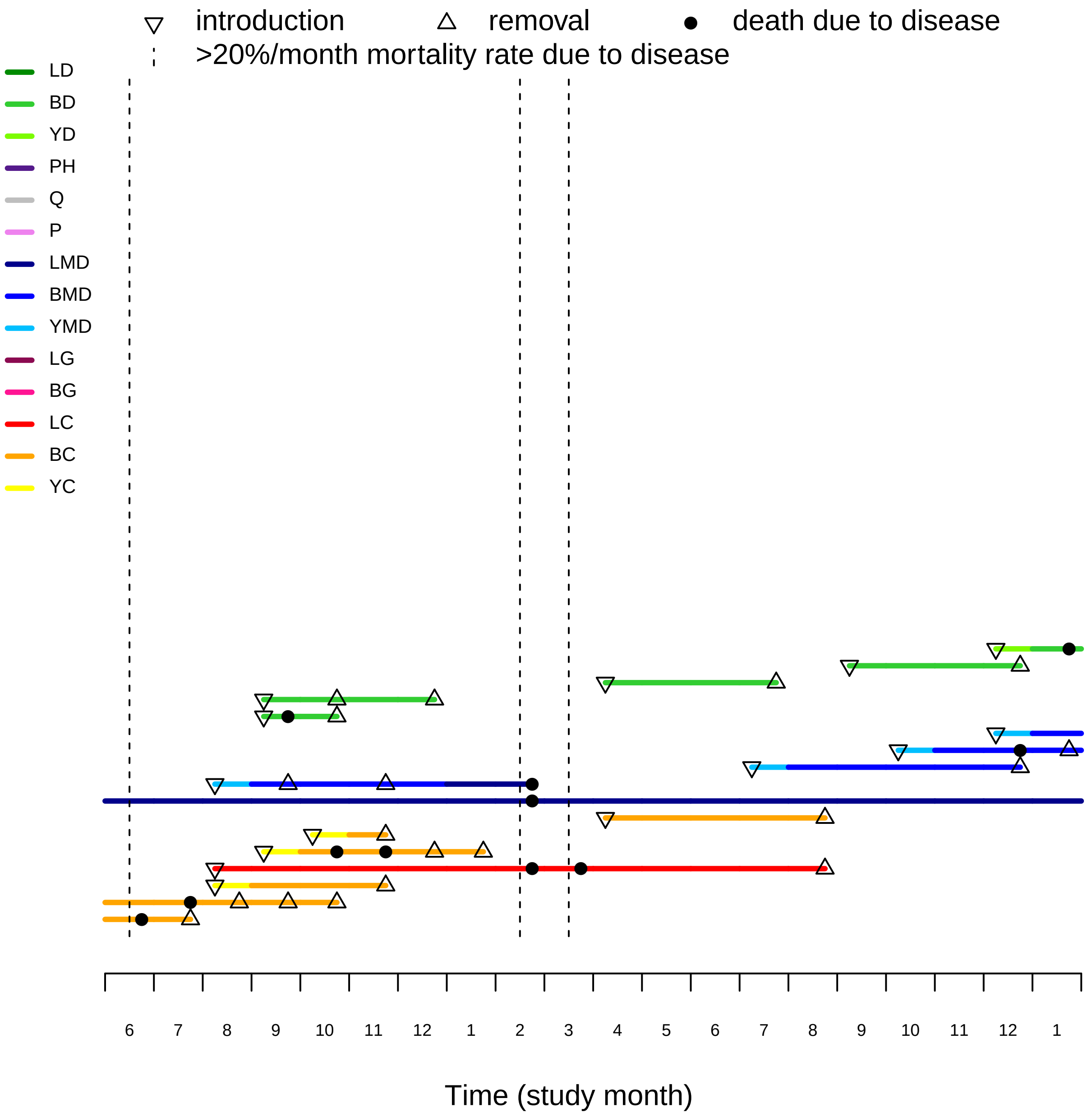

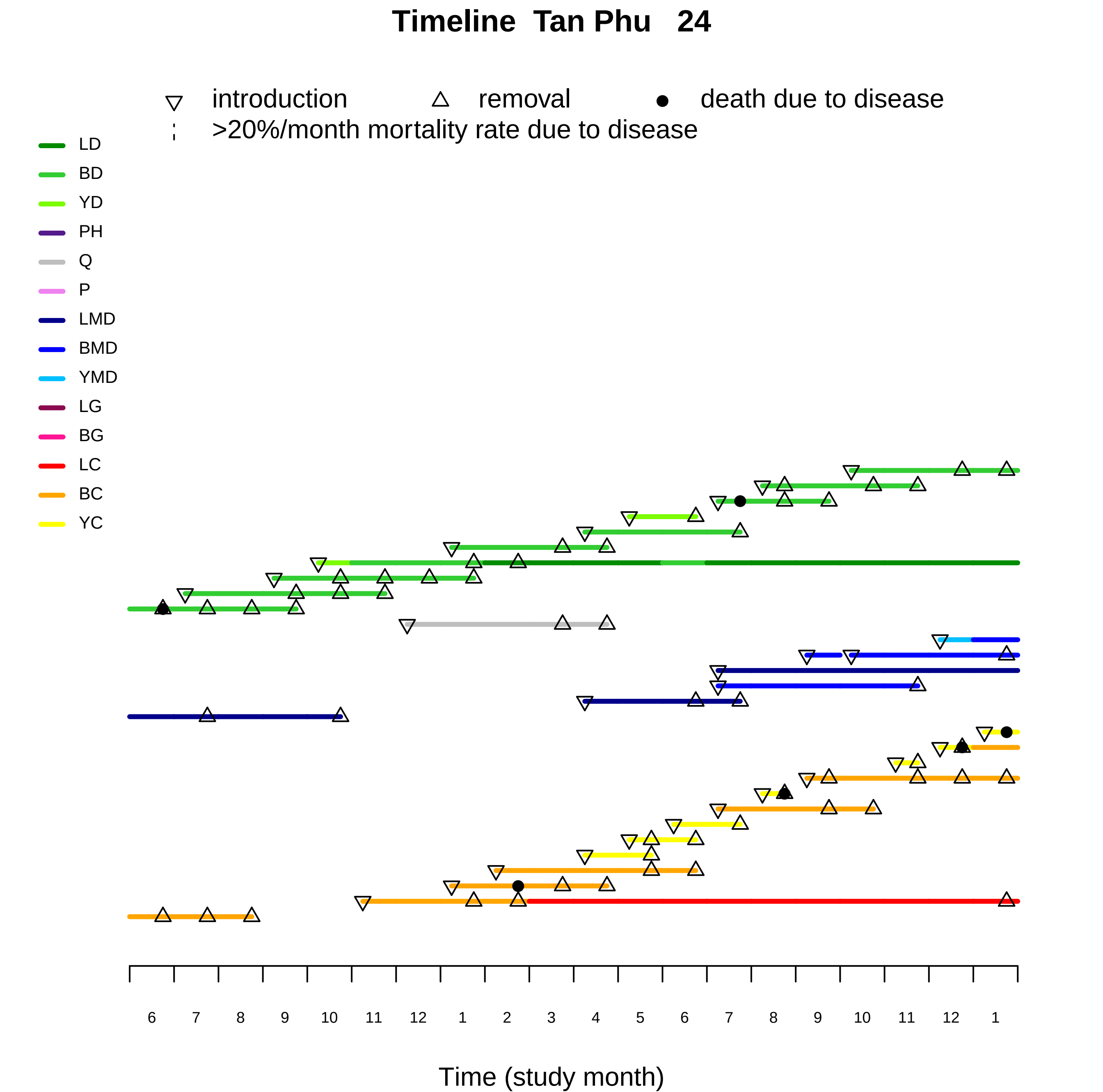
Timeline Tan Phu 24
introduction
removal
death due to disease
>20%/month mortality rate due to disease
LD
BD
YD
PH
Q
P
LMD
BMD
YMD
LG
BG
LC
BC
YC
6 7 8 9 10 11 12 1 2 3 4 5 6 7 8 9 10 11 12 1
Time (study month)

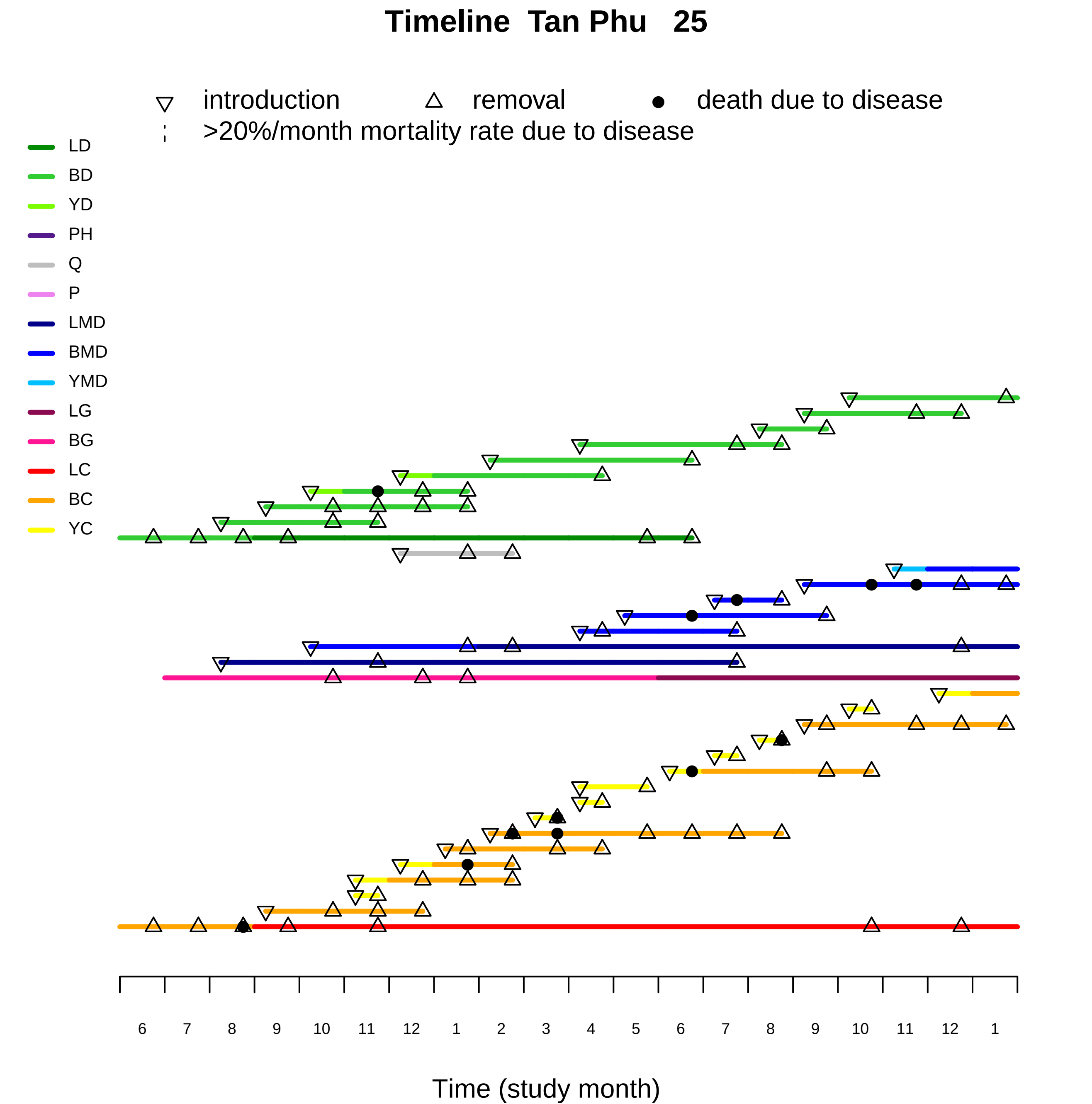

Timeline Tan Phu 25
introduction
removal
death due to disease
>20%/month mortality rate due to disease
LD
BD
YD
PH
Q
P
LMD
BMD
YMD
LG
BG
LC
BC
YC
6
7
8
9
10
11
12
1
2
3
4
5
6
7
8
9
10
11
12
1
Time (study month)

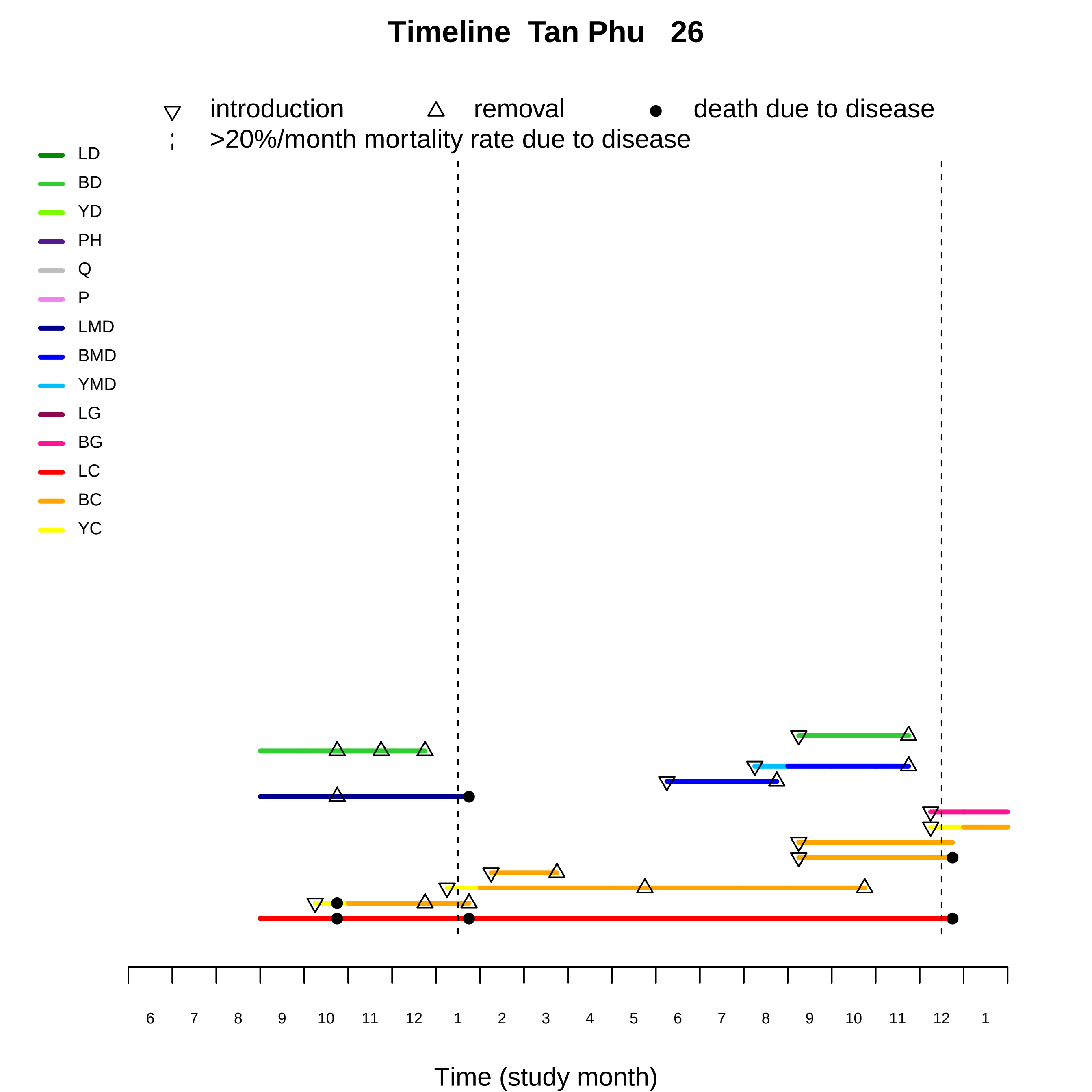
Timeline Tan Phu 26
introduction
removal
death due to disease
>20%/month mortality rate due to disease
LD
BD
YD
PH
Q
P
LMD
BMD
YMD
LG
BG
LC
BC
YC
6
7
8
9
10
11
12
1
2
3
4
5
6
7
8
9
10
11
12
1
Time (study month)

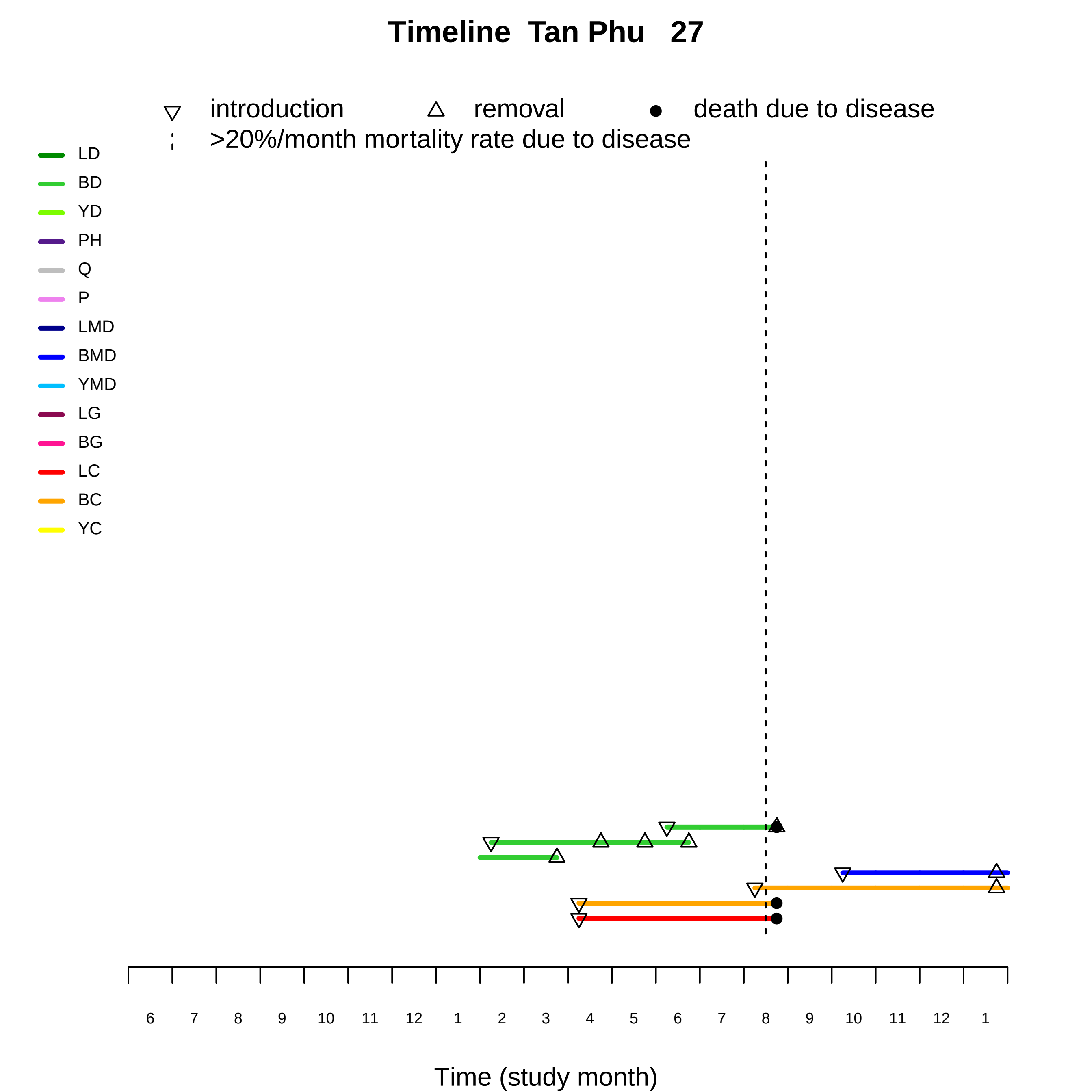
Timeline Tan Phu 27
introduction
removal
death due to disease
>20%/month mortality rate due to disease
LD
BD
YD
PH
Q
P
LMD
BMD
YMD
LG
BG
LC
BC
YC
6
7
8
9
10
11
12
1
2
3
4
5
6
7
8
9
10
11
12
1
Time (study month)
