# Supplementary material for: Poultry population dynamics and mortality risks in smallholder farms of the Mekong river delta region
Source: BMC Vet Res. 2019 Jun 17;15:205. doi: 10.1186/s12917-019-1949-y (PMC6580564; doi:10.1186/s12917-019-1949-y)

# Additional file 4

# Timeline of poultry flocks in each study commune

# (Tan Loc and Tan Phu)

Flocks are represented by straight lines starting on the month the first birds are introduced and finishing on the month the last birds are sold. Specific events (introduction, removal and deaths attributable to diseases) are displayed on each flock on the month they occurred.

**Code of poultry type and specie**

LD: layer duck

BD: broiler duck

YD: duckling

PH: pheasant

Q: quail

P: pigeon

LMD: layer Muscovy duck

NMD: broiler Muscovy duck

YMD: young Muscovy duck

LG: layer geese

BG: broiler geese

LC: layer chicken

BC: broiler chicken

YC: chicks

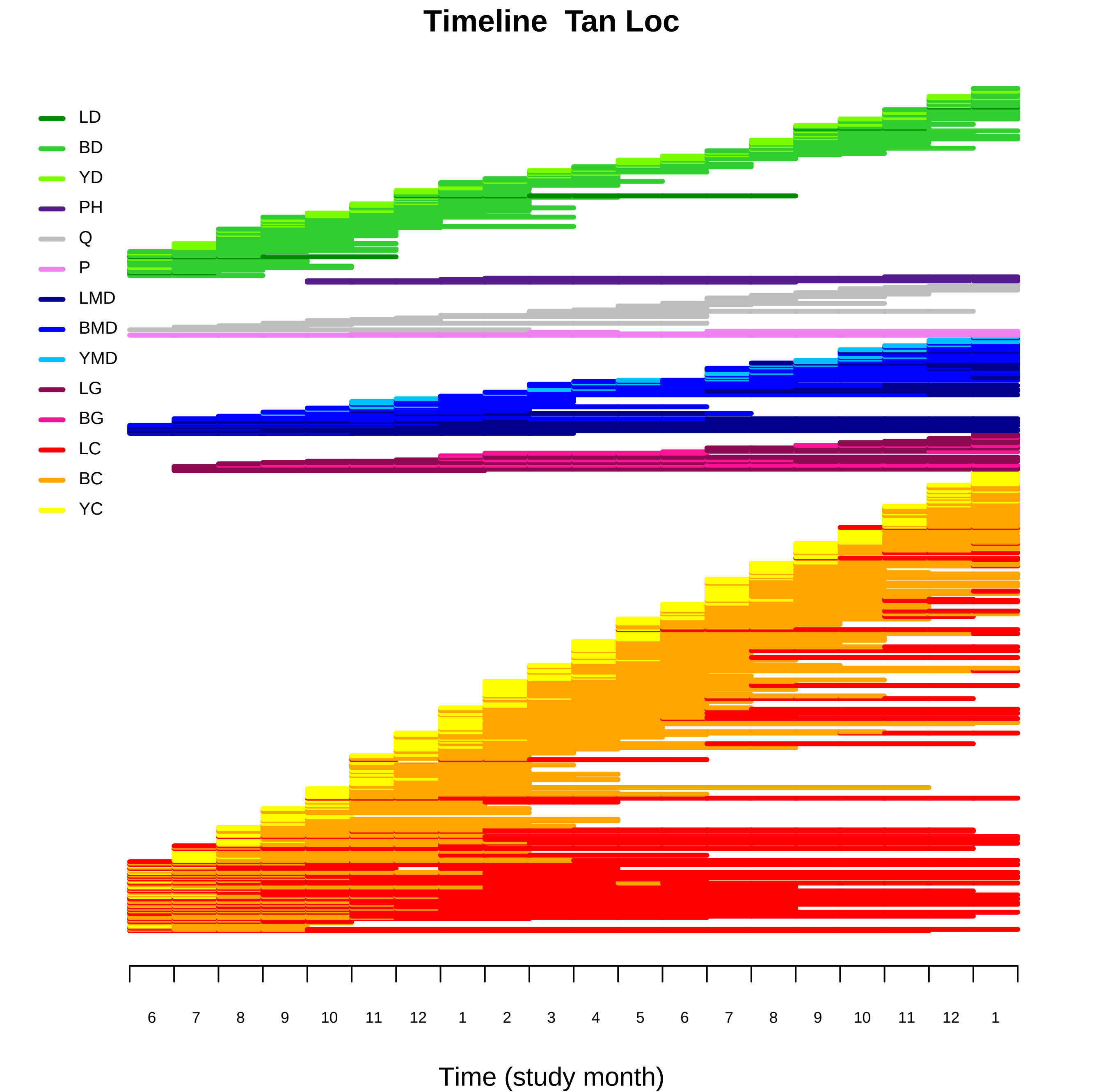

Timeline Tan Loc
LD
BD
YD
PH
Q
P
LMD
BMD
YMD
LG
BG
LC
BC
YC
6
7
8
9
10
11
12
1
2
3
4
5
6
7
8
9
10
11
12
1
Time (study month)

# Timeline  Tan Phu

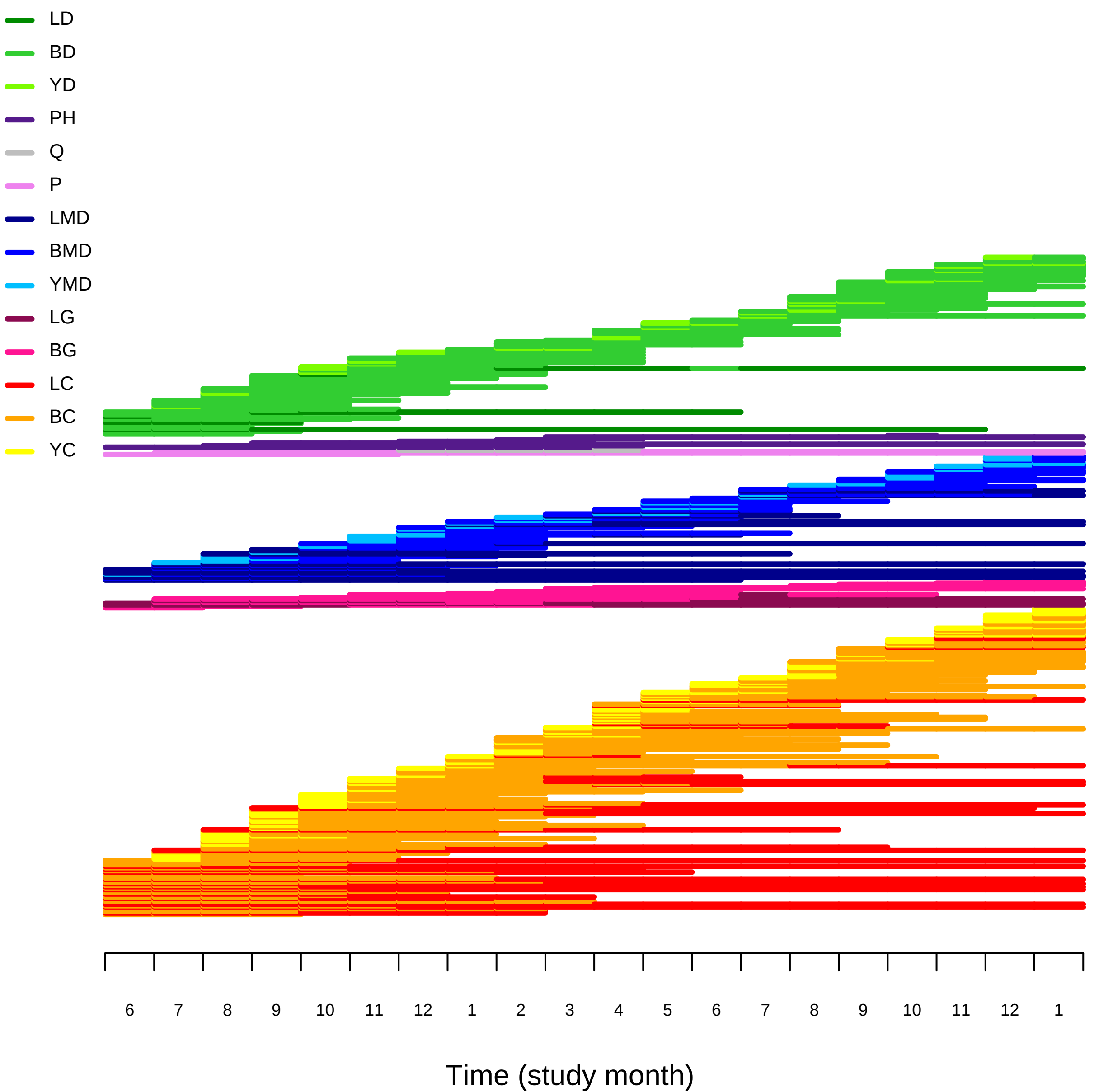

Supplement: Supplementary file 4 — Timeline of poultry flocks in each study commune. (PDF 276 kb) [file 12917_2019_1949_MOESM4_ESM.pdf]
