# Supplementary material for: Poultry population dynamics and mortality risks in smallholder farms of the Mekong river delta region
Source: BMC Vet Res. 2019 Jun 17;15:205. doi: 10.1186/s12917-019-1949-y (PMC6580564; doi:10.1186/s12917-019-1949-y)
Supplement: Supplementary file 5 — Characteristics of fitted probability distributions of flock sizes of the three main poultry species. (PDF 176 kb) [file 12917_2019_1949_MOESM5_ESM.pdf]

Additional file 5. Characteristics of fitted probability distributions of flock sizes of the three main poultry species

		**Chicken**	**Duck**	**Muscovy duck**
Real data	Mean	40	81	14
	Variance	4195	10642	108
Exponential	Variance	1628	6567	191
	**AIC***	**4690**	**2808**	**1068**
Gamma distribution	Variance	1939	7857	98
	**AIC***	**4682**	**2805**	**1038**
Mixture of two gamma distributions	Variance	4275	10483	113
	**AIC***	**4835**	**2753**	**1037**
Mixture of three gamma distributions	Variance	4861	9919	107
	**AIC***	**4544**	**2754**	**1031**

*AIC: Aikaike Information Criterion
